# Carved in stone: Experimental criteria for identifying Paleolithic *bas-relief* production techniques and sculptors’ expertise

**DOI:** 10.1371/journal.pone.0346099

**Published:** 2026-04-01

**Authors:** Émilie Brochard, Luc Doyon, Lloyd A. Courtenay, Gilles Tosello, Lila Geis, Francesco d’Errico

**Affiliations:** 1 Université de Bordeaux, UMR PACEA, CNRS, MCC, Pessac, France; 2 Universitat Rovira i Virgili, Department d’Història I Història de l’Art, Tarragona, Spain; 3 Université de Toulouse, UMR TRACES, CNRS, MCC, Toulouse, France; 4 University of Bergen, Centre for Early Sapiens Behaviour (SapienCE), Department of Archaeology, History, Cultural Studies and Religion, Norway; Sapienza University of Rome: Universita degli Studi di Roma La Sapienza, ITALY

## Abstract

Paleolithic *bas-relief* is a rare yet technically demanding form of parietal art whose production methods and skill requirements remain poorly understood. Investigating their production is essential because carving methods and required skills reveal the degree of technical investment, cognitive planning, and craftsmanship mobilized by prehistoric artists. This study presents the first integrated experimental, qualitative, and quantitative investigation of their manufacture. Using Coniacian limestone blocks, we replicated 19 carving modalities, covering pecking, scraping, polishing, engraving, and sequential combinations, executed by participants ranging from novices to a professional sculptor. Each surface was documented through high-resolution photography, Reflectance Transformation Imaging, and 3D scanning, then analyzed via standardized descriptive criteria, roughness measurements, and elliptical Fourier analysis of engraving profiles. Results show that some techniques, such as pecking and engraving, leave distinctive traces, while scraping and polishing often produce overlapping surface signatures, especially when techniques are superimposed. Quantitative data confirm a continuum of surface textures rather than discrete categories and reveal how the sequence of actions can partially obscure earlier marks. Expertise strongly influences mark regularity, efficiency, and isotropy, with the expert producing more controlled and less complex surfaces. The combined qualitative–quantitative approach proved essential: numerical parameters objectively discriminate patterns, but visual assessment remains critical for interpreting surface reliefs in archaeological contexts. By establishing experimentally validated diagnostic criteria for techniques and skill levels, this work provides a robust reference framework for identifying carving methods on archaeological *bas-reliefs*. The findings open new perspectives for reconstructing *chaînes opératoires*, assessing knowledge transmission, and exploring the role of specialization in Upper Paleolithic hunter-gatherer societies.

## 1. Introduction

**Fig 1:**
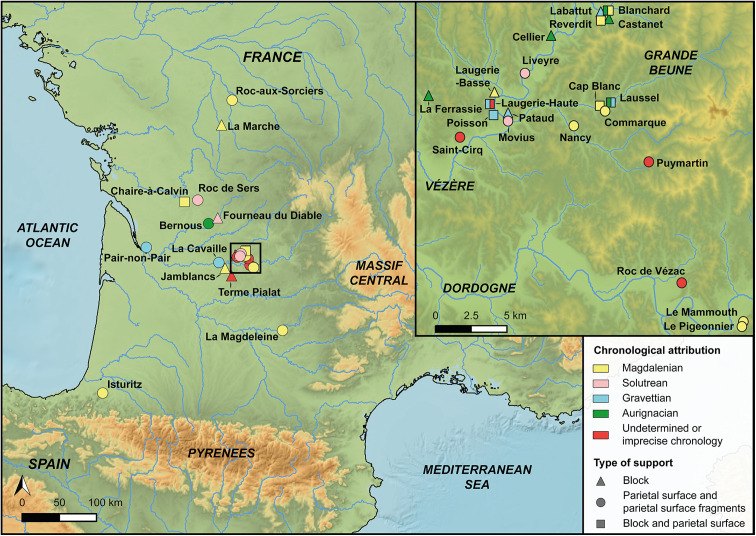
Geographic distribution of sites with parietal, boulder, or block sculptures in France. Note that portable sculptures are excluded from this overview. Map created with Qgis 3.36.3 “Maidenhead”. Background maps reprinted under a CC BY or equivalent license from: NOAA National Centers for Environmental Information. ETOPO 2022 15 Arc-Second Global Relief Model. 2022. doi: 10.25921/fd45-gt74; **M.** Zickel, **D.** Becker, **J.** Verheul, **Y.** Yener, **C.** Willmes. Paleocoastlines GIS dataset. CRC806-Database. 2016. doi: 10.5880/SFB806.19; European Environment Agency (EEA). European catchments and Rivers network system (Ecrins), natural sub basins of Europe – version 0, Dec. 2011. 2011; Institut national de l’information géographique et forestière (IGN). BD TOPO. 2017.

Often carved into limestone walls, Paleolithic *bas-reliefs* (low relief) still catch light and shadow much as they did tens of thousands of years ago, revealing the skill, vision, and patience of their makers. These figures, slightly projecting from the surface yet remaining part of it [1], form a striking but often overlooked facet of Paleolithic art. Found mainly in southwestern France, *bas-reliefs* appear at about thirty known sites (Fig 1; Table 1), from the earliest Aurignacian (ca. 41,500–33,000 cal BP [2,3]) examples to the more numerous middle Magdalenian works [4] dated to roughly 19,500–16,000 cal BP [5]. Among the most celebrated are Cap Blanc, Chaire-à-Calvin, Commarque, Laussel, Roc-aux-Sorciers, and Roc-de-Sers.

**Table 1:** Synthesis of French Upper Paleolithic sites with parietal or block sculptures: location, chronological attribution, type of site, support, and sculpture, along with iconography.

Site name	Location	Chronological attribution	Site type	Type of support	Type of sculpture^*^	Iconography	References
Blanchard	Sergeac, Dordogne	Aurignacian Cliff face: Magdalenian?	Rock shelter	Blocks Parietal surface	Incised relief Shallow modeled relief *Bas-relief*	Vulvae Indeterminate markings Cliff face: Indeterminate animal	[6–10]
Castanet	Sergeac, Dordogne	Aurignacian	Rock shelter	Blocks	Incised relief Shallow modeled relief	Vulvae, Phalli Indeterminate markings	[6–9,11]
Cellier	Tursac, Dordogne	Aurignacian	Rock shelter	Blocks	Incised relief Shallow modeled relie	Vulvae	[6,8,11]
La Ferrassie (Grand abri)	Savignac-de-Miremont Dordogne	Aurignacian	Rock shelter	Blocks	Incised relief Shallow modeled relief *Bas-relief*	Vulvae, Phalli Indeterminate markings	[6–9,11]
Les Bernous (or Les Bernoux)	Bourdeilles, Dordogne	Aurignacian?	Cave	Parietal surface	*Bas-relief*	Mammoth	[7–9]
Roc de Vézac	Vézac, Dordogne	Aurignacian or Gravettian?	Cave	Parietal surface	*Bas-relief*	Reniform signs	[9]
Terme Pialat (or Termo-Pialat)	Saint-Avit-Sénieur, Dordogne	Aurignacian or Gravettian?	Open-air	Block	Shallow modeled relief	Female figure, figure of indeterminate sex	[6–8,12,13]
Laussel	Marquay, Dordogne	Aurignacian and Gravettian	Rock shelter	Parietal surface fragment Blocks	Incised relief Shallow modeled relief *Bas-relief* (Sculpture in the round)	Aurignacian levels: vulvae, phalli, indeterminate markings (phallus?) Gravettian levels: female figures, figures of indeterminate sex Indeterminate levels: male figure, horse, indeterminate animals (In the round: phallus)	[6–9,14–25]
La Cavaille	Couze et-Saint-Front, Dordogne	Gravettian	Cave	Parietal surface	Shallow modeled relief	Mammoths	[8,11,26]
Labattut	Sergeac, Dordogne	Gravettian	Rock shelter	Block	Shallow modeled relief *Bas-relief*	Horse	[7–9,11,27]
Poisson	Les Eyzies, Dordogne	Gravettian	Rock shelter	Parietal surface Blocks	*Bas-relief*	Salmonid Indeterminate markings	[7–9,11,22,27]
Pair-non-Pair	Prignac-et-Marcamps, Gironde	Gravettian	Cave	Parietal surface	Shallow modeled relief *Bas-relief*	Horse, cervid	[7–9,11,28,29]
Abri Pataud	Les Eyzies, Dordogne	Gravettian	Rock shelter	Block	*Bas-relief*	Female figure	[30]
Laugerie-Haute	Les Eyzies, Dordogne	Blocks: Gravettian Sculpture in the round: undetermined Parietal surface: Solutrean?	Rock shelter	Parietal surface Small blocks (portable)	Incised relief Shallow modeled relief (Sculpture in the round)	Parietal surface: mammoth, indeterminate animals Small blocks: vulva, phallus (Sculpture in the round: musk ox)	[7–9]
Liveyre	Tursac, Dordogne	Solutrean?	Cave	Parietal surface	*Bas-relief*	Indeterminate animal	[31,32]
Fourneau du Diable	Bourdeilles, Dordogne	Solutrean	Rock shelter	Block	*Bas-relief* *Haut-relief*	Vulva Aurochs, horse, indeterminate ungulate indeterminate animals	[7–9,11]
Roc de Sers	Sers, Charente	Solutrean	Rock shelter	Parietal surface Parietal surface fragments	Incised relief Shallow modeled relief *Bas-relief* *Haut-relief*	Figures of indeterminate sex Bison, indeterminate bovine, ovibos-bison, bison-wild boar, horses, ibex, reindeer, bird, indeterminate animals Indeterminate markings	[7–9,33,34]
Abri Movius (or Cave troglodytique Pataud)	Les Eyzies, Dordogne	Solutrean	Rock shelter	Parietal surface	*Bas-relief*	Ibex	[8,11]
Puymartin	Marquay, Dordogne	Solutrean or Magdalenian?	Cave	Parietal surface	*Bas-relief*	Horses	[31,35]
Saint-Cirq	Saint-Cirq-du-Bugue, Dordogne	Solutrean or Magdalenian?	Cave	Parietal surface	*Bas-relief* (Sculpture in the round in Roc-Saint-Cirq)	Bison, horse, indeterminate animals (Sculpture in the round: turtle)	[4,8,9,36]
Nancy	Les Eyzies, Dordogne	Magdalenian?	Cave	Parietal surface	*Bas-relief*	Horse	[4,7–9,37,38]
Laugerie-Basse (Abri des Marseilles)	Les Eyzies, Dordogne	Magdalenian	Rock shelter	Block	*Bas-relief*	Horse	[7,9,39]
Cap Blanc	Marquay, Dordogne	Magdalenian	Rock shelter	Parietal surface Parietal surface fragment Block	Shallow modeled relief *Bas-relief* *Haut-relief*	Parietal surface: bison, indeterminate bovines, horses, indeterminate animal Parietal surface fragment: horse Block: bison	[4,7,8,21,22,31,40,41]
Commarque (or Comarque)	Les Eyzies, Dordogne	Magdalenian	Cave	Parietal surface	Incised relief Shallow modeled relief *Bas-relief*	Female representation, vulvae Horses, ibex, indeterminate animal	[4,7–9,37,42]
Reverdit	Sergeac, Dordogne	Magdalenian	Rock shelter	Parietal surface Blocks	*Bas-relief*	Parietal surface: indeterminate ungulate, horse, bison Blocks: bison, indeterminate ungulate, indeterminate animal	[4,7–9,31,43]
Le Mammouth (or Grande Grotte de Saint-Front)	Domme, Dordogne	Magdalenian	Cave	Parietal surface	Shallow modeled relief *Bas-relief*	Mammoths, indeterminate ungulates, indeterminate animals Circular sign	[4,8,9,32,44]
Le Pigeonnier	Domme, Dordogne	Magdalenian	Cave	Parietal surface	Incised relief Shallow modeled relief *Bas-relief*	Horse, herbivores, mammoth	[4,8,9,32,44]
Roc-aux-Sorciers	Angles-sur-l’Anglin, Vienne	Magdalenian	Rock shelter	Parietal surface Parietal surface fragment	Incised relief Shallow modeled relief *Bas-relief* *Haut-relief* (Sculpture in the round)	Abri Bourdois: Female figures, human heads, bison, horses, ibex, indeterminate caprines, felines Cave Taillebourg: Vulva, human head, bison, horses, ibex, felines, indeterminate animals (Sculpture in the round: inderteminate animals)	[7–9,24,31,34,45–48]
La Marche	Lussac-les-Châteaux, Vienne	Magdalenian	Rock shelter	Block Small blocks (portable)	Incised relief Shallow modeled relief *Bas-relief*	Block: feline Small blocks: mammoth	[47,49–52]
Chaire-à-Calvin	Mouthiers-sur-Boëme, Charente	Magdalenian	Rock shelter	Parietal surface Block	Shallow modeled relief *Bas-relief*	Parietal surface: horse, horse-bison, ibex Block: indeterminate animal	[4,7–9,31,34,53–57]
Isturitz	Saint-Martin d’Arberoue, Pyrénées-Atlantiques	Magdalenian	Cave	Parietal surface	Incised relief Shallow modeled relief *Bas-relief*	Horse, cervids, wolverine, bovines, reindeer, fish, indeterminate animals Sign, lines	[4,7–9,58]
La Magdeleine (or Magdeleine des Albis ou Magdelaine)	Penne, Tarn	Magdalenian	Cave	Parietal surface	*Bas-relief*	Female figures Horse, bison	[8,9,24,59]
Jamblancs (or Jean-Blancs or Champs-Blancs)	Bourniquel, Dordogne	Magdalenian	Rock shelter	Blocks	*Bas-relief*	Bison	[7,60]

Table adapted and expanded from Tymula [33].

* The definitions of the sculpture types are adapted from Baudry [1], Iakovleva and Pinçon [45], Tymula [33], and Bourdier [31]. **Incised relief**: the volume is suggested by linear engraved lines rather than by true modeling or projection. The figure remains within the plane of the support. **Shallow modeled relief**: The subject’s contours are incised and gently shaped, remaining within the same plane as the support. The figure is suggested through subtle modeling rather than projection. ***Bas-relief*** (low relief**):** A form of relief in which the projecting shapes, whether attached to a flat, convex, or concave background, represent less than half the actual volume of the depicted body or object. ***Haut-relief*** (high relief): A form of relief in which the projecting shapes, whether attached to a flat, convex, or concave background, represent more than half but less than three-quarters of the actual volume of the depicted body or object. **Sculpture in the round**: A sculpture that represents at least three-quarters of the actual volume of a body or object. It may be fully worked on all sides (front, sides, and back), or only on three aspects (front and sides). Unlike most reliefs, it is not attached to a background.

Although far less common than paintings or engravings, *bas-reliefs* stand out for their technical complexity and artistic sophistication. Sculptors shaped their subjects with precise control over depth, perspective, and surface detail, often engaging with the natural contours of the rock to create dynamic effects under shifting light [61]. More durable than paintings, these carvings have nonetheless suffered from weathering and other erosive agents in open-air contexts, providing a valuable record of the taphonomic processes [62,63] that affect prehistoric art.

The very existence of *bas-reliefs* raises a fundamental anthropological question: why would highly mobile terrestrial hunter-gatherers invest so much time, energy, and skill in creating large, immovable works of art? In other contexts, such undertakings are linked to relatively sedentary societies with predictable resource bases. Among the many possible examples, the fishing communities of the Northwest Coast of North America [64] stand out, as their seasonal surpluses and prey preservation practices supported the creation of monumental wooden sculptures and totem poles [65,66]. In contrast, Upper Paleolithic sculptors were terrestrial foragers who often moved across large territories. Did they organize work in ways that allowed for specialized artisans? Did such carvings play roles in social negotiation, identity marking, or symbolic communication [31] that outweighed the costs of their production? Addressing these questions could fundamentally reshape our understanding of labor organization, social complexity, and the role of art in mobile societies [67,68].

At present, the analytical tools available for exploring and studying these issues remain limited. Initial research relied on iconographic and stylistic analyses [e.g., 7, 8, 14, 24, 40, 46, 69], while more recent studies have explored possible carving techniques inferred from morphological features [e.g., 33, 11, 25, 41, 45, 70]. As illustrated by analyses of tools and gestures in engraving, drawing, and painting, it is possible to reconstruct the sequence of techniques used in graphic production [e.g., 71–73]. The interpretations proposed for sculpture, however, remain speculative without experimental validation. Advances in 3D recording [74–77] have greatly improved documentation, but the potential of these data to provide insight into production methods has not been fully explored. Some experimental studies have investigated the tools used to produce sculptures [78,79] and the marks produced by specific carving techniques [6,11,33,80–84]. However, despite these studies, experimental archaeology, which has yielded breakthroughs for understanding engravings and percussion-based petroglyphs [79,85–93], has yet to develop a systematic, reproducible framework for studying parietal sculpture. Nor has it been used to evaluate the skill levels of prehistoric artists, an area where engraving studies have proposed criteria to distinguish novices from experts [86,92,94–99].

Here, we address these gaps through a standardized experimental protocol. Twelve carving modalities (one technique with one tool each), plus seven selected combinations, were reproduced under controlled conditions by individuals with different levels of experience. The resulting traces were analyzed both qualitatively, using systematic descriptive criteria, and quantitatively, through surface roughness measurements and elliptical Fourier analysis of micro-topographic profiles. The variables identified through these experiments provide an empirical reference for exploring differences between techniques and evaluating carving proficiency. Our methodological approach offers new perspectives for studying Paleolithic *bas-reliefs*, enabling archaeologists to interpret them with greater precision, reconstruct their *chaîne opératoire*, and explore broader questions about the technical knowledge, skill transmission, and social organization of Upper Paleolithic hunter-gatherers.

## 2. Materials and methods

### 2.1. Experimental protocol

To investigate the traces observed on Paleolithic sculptures, we designed a controlled experimental protocol to test different carving techniques and tools on limestone. We used ten standardized blocks (30 × 30 × 6 cm) of coarse Coniacian bioclastic limestone [100,101] sourced from Carrières Vèze, Les Eyzies, a quarry located less than 3 km from the Laussel site, the main archaeological context of our research. Each block featured a flat surface, which was subsequently divided into nine 10 × 10 cm squares in preparation for the experiments, while retaining only the sawing marks from extraction (S1 Fig in S1 File). Although Paleolithic bas-reliefs were carved on irregular natural surfaces, industrially cut limestone blocks were selected to provide standardized, comparable substrates. This controlled material minimizes variability in surface morphology and physical properties, enabling rigorous comparisons of carving techniques and gestures, and allows assessment of how their application to standardized surfaces affects the resulting textural data.

Experiments were carried out in two phases at the Pôle Mixte de Recherche Archéologique in Campagne (Dordogne) on September 7, 2022, and the PACEA laboratory, University of Bordeaux on October 2 and 7, 2024. Each phase involved five right-handed participants (two women, three men), recruited based on availability and prior experience in sculpting and/or engraving, with the aim of covering a broad range of experience rather than producing a statistically representative sample of sculptors. Participants were assigned to three experience categories (Table 2), novice (no prior experience), intermediate (some prior sculpting and/or engraving experience), and expert (a professional stone sculptor with more than 15 years of experience)—resulting in one expert, two intermediates, and two novices per phase. These categories were intended as broad experiential groupings rather than formal evaluative measures of technical skill. Participants worked seated or crouched with blocks inclined at 60° on sandbag supports. One intermediate participant worked standing but with the same block orientation. All participants except the expert and one novice wore gloves. To focus on technical traces rather than iconography, the protocol deliberately excluded figurative representations, limiting variability related to iconography and compositional choices and allowing robust comparisons between techniques.

**Table 2:** Information on participants involved in the experimental protocol, including assigned skill level, sex, age, handedness and participation phase.

Experimenter	Sex	Laterality	Age	Participation
Novice 1	Female	Right-handed	35	Phase 1
Novice 2	Female	Right-handed	31	Phases 1 and 2
Novice 3	Male	Right-handed	45	Phase 2
Novice 4	Female	Right-handed	27	Phase 2
Intermediate 1	Male	Right-handed	68	Phase 1
Intermediate 2	Male	Right-handed	67	Phases 1 and 2
Expert 1	Male	Right-handed	38	Phases 1 and 2

Each experimental modality, defined as a specific combination of technique(s) and tool(s), was applied to a 10 × 10 cm square. With the exception of the engravings, participants were asked to work systematically across the entire allocated surface of each experimental square. A total of 19 modalities (Table 3) were tested, each performed by five participants across the two phases, yielding 95 trials. Participants were assigned personal tool set, prepared in advance and reused throughout, with no sharing between individuals. The participants were allotted five minutes to apply each technique, an arbitrary duration chosen to standardize practice time across individuals, followed by an additional five minutes for each superimposed technique. The five-minute duration was selected based on preliminary trials, which showed that continuous work for five minutes was sufficient to substantially and visibly modify the entire 10 × 10 cm working surface of the limestone blocks. This duration thus ensured that each technique could be meaningfully expressed while maintaining comparability across individuals and modalities. Although the chosen duration is necessarily arbitrary, this approach allows robust comparisons between techniques under standardized conditions and within the practical constraints of the experimental design (e.g., participant availability). Engraving trials could be stopped earlier at the participant’s discretion.

**Table 3:** Summary of experimental modalities, showing combinations of techniques and the tools used.

Technique Code	Technique Description	Tool(s) Used (Active Part)
PDP	Pecking (direct percussion)	Pick (point)
		Cobble
PIP	Pecking (indirect percussion)	Pick (point)
		Broken blade (fracture)
Sc	Scraping	Blade (unretouched edge)
		Endscraper (retouched edge)
Ems	Engraving (multiple strokes)	Burin (point)
		Flake (unretouched edge)
		Blade (unretouched edge)
Po	Polishing	Cobble
		Tanned skin
		Wet sand
PDP + Ems	Pecking + Engraving (multiple strokes)	Broken blade (fracture) + Burin (point)
		Broken blade (fracture) + Pick (point)
PDP + Sc	Pecking + Scraping	Pick (point) + Endscraper (retouched edge)
PDP + Sc + Po + Ess	Pecking + Scraping + Polishing + Engraving (single stroke)	Pick (point) + Endscraper (retouched edge) + Tanned skin + Blade (unretouched edge)
PDP + Sc + Po + Ems	Pecking + Scraping + Polishing + Engraving (multiple strokes)	[Same as above]
PDP + Sc + Ess + Po	Pecking + Scraping + Engraving (single stroke) + Polishing	Pick (point) + Endscraper (retouched edge) + Blade (unretouched edge) + Tanned skin
PDP + Sc + Ems + Po	Pecking + Scraping + Engraving (multiple strokes) + Polishing	[Same as above]

Unless otherwise specified, all tools employed in the experiments, across both phases, were made of flint.

**Fig 2:**
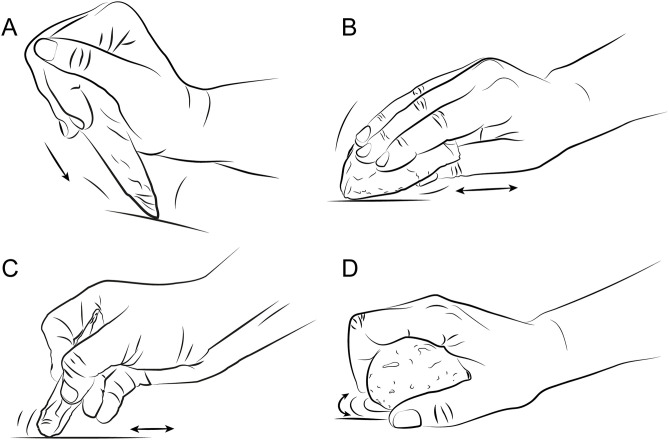
Techniques tested during the experiments. **(A)** Pecking, **(B)** Scraping, **(C)** Engraving and **(D)** Polishing.

Phase 1 (S1 Fig in S1 File) examined pecking, which consists of striking the rock surface perpendicularly or obliquely with a pointed implement to detach small fragments [1] (Fig 2A). Three implements were employed:

- (i) a pick in direct percussion,
- (ii) the same pick in indirect percussion with an organic soft hammer (wooden billet)
- (iii) a broken blade used as a point.

Picks were selected based on archaeological parallels from sites with *bas-reliefs* [e.g., 102–110]. Scraping, defined as removing surface irregularities by rubbing the edge of a tool over the stone [1] (Fig 2B) was tested with both (i) an unretouched blade edge and (ii) an endscraper. Engraving — incising the surface to produce linear marks [1] (Fig 2C) — was explored in:

- Superficial engravings: using (i) the tip of a simple or dihedral burin, (ii) an unretouched flake tip, and (iii) an unretouched blade edge (burin and flake tested on the same square).
- Deep engravings: the right edges of two squares were first lowered by pecking with a broken blade, then incised, with (i) a burin and (ii) a pick (the active parts were larger than for superficial engravings).

Phase 2 (S1 Fig in S1 File) introduced pecking with a quartz cobble in direct percussion, reflecting the abundance of cobbles at sculpted sites [e.g., 54, 78, 111]. Polishing, aimed at reducing surface irregularities by rubbing the surface to leave minimal visible marks [1] (Fig 2D), was carried out with tanned skin, quartz pebbles or wet sand. For the latter two materials, this process corresponds to abrasive polishing. Before each test, one of us (ÉB) scraped the relevant surfaces with a blade for five minutes to produce a rough surface with which to polish. Participants also tested sequential combinations of techniques including:

1) pecking with a pick followed by scraping with an endscraper,
2) pecking and scraping followed by engraving with a blade and then, polishing with tanned skin,
3) pecking and scraping followed by polishing and then, engraving.

Engravings were executed using both single and multiple strokes, in straight and curvilinear forms.

The sequence and combination of techniques tested in this experiment were designed to follow a technically and operationally coherent workflow consistent with bas-relief production. Pecking was considered a roughing technique, scraping an intermediate shaping stage, and polishing a finishing process. Consequently, polishing was not applied directly to surfaces subjected only to pecking, as such a sequence does not correspond to the operative logic of sculptural production, in which finishing does not immediately follow roughing but is typically preceded by surface regularization. From an experimental standpoint, applying polishing directly to a pecked surface under the standardized time constraints of the protocol would likely result either in the persistence of pecking traces if applied briefly, or in surface states comparable to those obtained after a pecking–scraping–polishing sequence if applied for longer durations. Such a combination was therefore considered methodologically redundant and was not included in the experimental design.

Throughout the experiment, observations on tool performance, gestures (orientation, movement, hand use), and participant feedback were documented. All trials were video-recorded using a Sony α6500 camera with a Sony E 30-mm F3.5 macro lens. Post-experiment, surfaces were vacuum-cleaned to remove loose particles that might obscure the traces.

Because the present study involves human subjects to carry out experimental tasks, an agreement with the ERC ethics guidelines of the ERC SyG QUANTA was obtained. All participants were provided with a written consent form which they signed.

### 2.2. Qualitative analysis

Experimental marks were examined with the naked eye under various raking light conditions. We also applied the Reflectance Transformation Imaging (RTI) method described by Looten [112] to enhance surface detail. Photographs were captured using a Canon EOS 750D with a Canon EF 50 mm lens, compiled using *Relight* (Visual Computing Lab), and analyzed with *RTIViewer* (Cultural Heritage Imaging).

For each worked area, we documented its dimensions, surface homogeneity, and the orientation, directionality, and parallelism of the marks produced by each technique. For pecking, we recorded the qualitative characteristics of individual impact marks (cross-section, overall contour) and also noted basic measurements (length and width using manual callipers, depth categorized as shallow, medium, deep) to support the visual analysis. For scraping and polishing, we documented the characteristics of the striations and recorded simple measurements such as their average length and spacing whenever possible. For engraving*s*, we noted the depth (shallow, medium, deep), width (narrow, medium, wide), cross-sectional shape (“V”, “U”), and symmetry or asymmetry (right, left) as well as start and end features of the incisions [93]. When visible, internal features such as Hertzian cones and striations were also documented.

### 2.3. Quantitative analysis

The experimental surfaces were scanned using a Space Spider (Artec, Luxembourg), a portable 3D scanner employing structured-light technology. The resulting models reached a resolution of up to 100 μm. The files were saved in STL format.

All statistical analyses were performed using the R programming language. Throughout the entire study, hypotheses were tested using a significance threshold of *p* < 0.003 to reject the null hypothesis (*H₀*) in favor of the alternative (*Hₐ*) [113,114], rather than the conventional *p* < 0.05, whose reliability has been increasingly challenged [115].

#### 2.3.1. Roughness analysis.

Post-acquisition surface treatment was performed with MountainsMap premium 8.1.9286 software (Digital Surf, Besançon) and followed a procedure adapted from Mazzucco et al. and Doyon et al. [116,117].Within each 10 × 10 cm experimental modality square, the surface was digitally subdivided a second time into nine non-overlapping 10 × 10 mm sub-areas (S2 Fig in S1 File). The dimensions of these sub-areas were chosen to make our protocol applicable to archaeological surfaces, which can sometimes be relatively small. For this analysis, only sub-areas containing the worked surface were considered, thereby excluding untouched areas. Using integrated operators, each sub-area was processed following a standard protocol that entails levelling the surface (least square method) and removing form (polynomial of third order; see S1 Text in S1 File for justification). Fourteen roughness parameters (ISO 25178), eight fractal parameters (SSFA), four furrow analysis parameters, and three texture direction parameters, were extracted for each sub-area (Table 4 for definitions). Parameters expressed as percentages or ratios were transformed and centered around zero to remove the constraints imposed by their hard bounded nature, making them more suitable for certain univariate and multivariate statistical analyses. For this purpose, we use the logit transformation [118,119], where ratios (*p*) and percentages (rescaled to the [0, 1] interval where necessary) are used to calculate (eq. 1);

**Table 4:** Surface roughness parameters: codes, types of analyses, descriptions, units, and variables retained based on technique or expertise analysis.

Parameter code	Analysis	Description	Technique		Expertise	
			Significant parameters	Uncorrelated parameters	Significant parameters	Uncorrelated parameters
Sq	ISO 25178 (height parameter)	Root mean square height (mm)	X		X	
Smc	ISO 25178 (functional parameter)	Inverse areal material ratio (mm)	X		X	
Sal	ISO 25178 (spatial parameter)	Autocorrelation length (mm)	X	X	X	X
Str	ISO 25178 (spatial parameter)	Texture aspect ratio (unitless)	X	X	X	
Std	ISO 25178 (spatial parameter)	Texture direction (°)				
Sdr	ISO 25178 (hybrid parameter)	Developed interfacial area ratio (%)	X		X	
Vvv	ISO 25178 (volume parameter)	Dale void volume (mm³/mm²)	X		X	
Spd	ISO 25178 (feature parameter)	Density ok peaks (1/mm²)	X		X	
Spc	ISO 25178 (feature parameter)	Arithmetic mean peak curvature (1/mm)	X	X		
Svd	ISO 25178 (feature parameter)	Density of pits (1/mm²)	X		X	
Shrn	ISO 25178 (shape parameter)	Mean hill roundness (unitless)				
Shrnq	ISO 25178 (shape parameter)	Standard deviation of hill roundness (unitless)	X	X		
Sdrn	ISO 25178 (shape parameter)	Mean dale roundness (unitless)				
Sdrnq	ISO 25178 (shape parameter)	Standard deviation of dale roundness (unitless)	X	X		
Y-max	Scale-sensitive fractal	Maximum Y-value, relative length or area (unitless)	X		X	
Asfc	Scale-sensitive fractal	Fractal complexity (unitless)	X		X	
Das	Scale-sensitive fractal	Fractal dimension (unitless)	X		X	
Smfc	Scale-sensitive fractal	Scale of max complexity (µm²)	X	X	X	X
HAsfc	Scale-sensitive fractal	Heterogeneity of Asfc (3x3; unitless)	X	X	X	X
Median Asfc	Scale-sensitive fractal	Median of Asfc (3x3; unitless)	X	X	X	X
epLsar	Scale-sensitive fractal	Length-scale anisotropy (40 µm, 5°; unitless)	X			
NewEplsar	Scale-sensitive fractal	New length-scale anisotropy (40 µm, 5°; unitless)	X	X	X	X
Isotropy	Texture direction	Texture isotropy (%)	X		X	X
First direction	Texture direction	Dominant direction of surface structure (°)			X	X
Second direction	Texture direction	Secondary orientation of surface structure (°)				
Third direction	Texture direction	Tertiary orientation of surface structure (°)				
Maximum depth of furrows	Furrows	(mm)	X		X	
Mean depth of furrows	Furrows	(mm)	X		X	
Mean density of furrows	Furrows	(cm/cm^2^)	X	X	X	X

“X” in the columns ‘Significant parameters’ and ‘Uncorrelated parameters’ indicates variables retained based on the analysis of technique or expertise.


$$\text{logit}\left(p\right)=log\left(\frac{p}{1-p}\right)$$ (1)

The statistical analyses of roughness data followed a procedure adapted from Doyon et al. [117]. The same methodology was applied to the study of techniques and skill levels. Statistical methods were selected based on the nature of each variable (linear or circular) and the characteristics of their distributions. For linear variables, distribution normality was first assessed using the Shapiro-Wilk test. Depending on whether the data conformed to a Gaussian distribution or not, appropriate statistical methods were selected: parametric tests (ANOVA) [120,121] and traditional descriptive metrics for normally distributed variables, and non-parametric tests (Kruskal-Wallis) [122] with robust statistics in the case of non-normal distributions. For circular variables, the Mardia–Watson–Wheeler test [123,124] and Fisher’s non-parametric test [125] were used to evaluate distributional properties of the samples.

To conduct the multivariate analyses, only the relevant roughness parameters were included. Variables that show evidence of group differences in the Kruskal-Wallis, ANOVA or Mardia-Watson-Wheeler tests were selected. Among these, variables showing high correlation (*R²* ≥ 0.7) were also excluded based on a correlation matrix, ensuring that only one variable from each correlated pair was retained. For the correlation analyses, circular-to-circular correlations were calculated using the Jammalamadaka-Sarma method [126,127], and linear-to-circular correlations were calculated using the Johnson-Wehrly-Mardia method [128,129]. P-values were determined using randomized permutations. To integrate the circular data into the multivariate analysis, the variables were projected into a linear space. This was done by first converting the angle measurements from degrees to radians, then applying a linear transformation using the sum of the cosine and sine of each radian angle (θ), as follows [130]: $\theta_{lin}=cos\theta +sin\theta$. A Principal Component Analysis (PCA; confidence level = 0.95) was subsequently conducted.

For the purpose of testing whether the quantitative variables are sufficient for the discrimination between different groups, a *k*-fold (*k* = 10) cross-validated Linear Discriminant Analysis (LDA) was performed [131–134]. The first ten principal components (for the technique analysis) and the first seven principal components (for the expertise analysis) from the preceding PCA were retained as input variables for this purpose. The number of PC scores was selected as they collectively explained more than 95% of the total variance [135]. The ordination counterpart of LDA, Canonical Variate Analysis (CVA), was then used for visualisation purposes, using jack-knifed cross-validation for the computation of CV scores. For model evaluation, the dataset was split into a training set (80%) and a test set (20%) to evaluate the model’s predictive performance on independent data. Classification results were quantified using a confusion matrix and supplemented by overall statistics, including accuracy, the No Information Rate (NIR) and the Kappa coefficient, which was used because the dataset was balanced (0.92–0.98) [136–138]. Accuracy measures the proportion of correctly classified samples. The Kappa coefficient quantifies the agreement between predicted and actual classifications, taking into account the likelihood of chance agreement, with 0.8 considered an acceptable threshold for near-perfect classification [137]. The NIR represents the proportion of samples in the largest class and serves as a baseline; an accuracy below the NIR indicates performance no better than random classification by majority class. Class-specific metrics were also calculated: sensitivity (true positive rate), specificity (true negative rate), and precision (positive predictive value). These metrics provide a detailed assessment of the model’s ability to correctly identify each class and avoid misclassification.

#### 2.3.2. Engraving analysis.

The experimental engravings were organized into nine sets, each corresponding to a modality (see Table 3). To facilitate reading, these sets were renamed more concisely as Set A, Set B, etc. (Table 5).

**Table 5:** Original engraving modalities and their renamed sets.

Set	Engraving type	Technique description	Engraving stroke count	Engraving tool (active part)
Set A	Superficial	Engraving	Multiple strokes	Burin (point)
Set B				Flake (unretouched edge)
Set C				Blade (unretouched edge)
Set D		Pecking + Scraping + Polishing + Engraving	Single stroke	Blade (unretouched edge)
Set E			Multiple strokes	
Set F		Pecking + Scraping + Engraving + Polishing	Single stroke	
Set G			Multiple strokes	
Set H	Deep	Pecking + Engraving	Multiple strokes	Burin (point)
Set I				Pick (point)

Analysis of engravings, conducted on morphological variables extracted from profiles, followed a procedure adapted from Courtenay et al. [139]. Profile extraction was performed with MountainsMap premium 8.1.9286 software (Digital Surf, Besançon). The surfaces were levelled using the least squares method. For each engraving modality (Tables 3 and 5), two profiles were taken in areas displaying regular incisions without tool chatter marks, as close to the midpoint of the mark as possible. When the experimenters made multiple engravings within the square modality, the sections were taken from two different incisions. Conversely, when only one engraving was made, the profiles were taken from the same incision. Due to the limited dataset (only two measurements per engraving modality for the expert), the quantitative analysis was conducted for the techniques only, and not for the skill levels.

**Fig 3:**
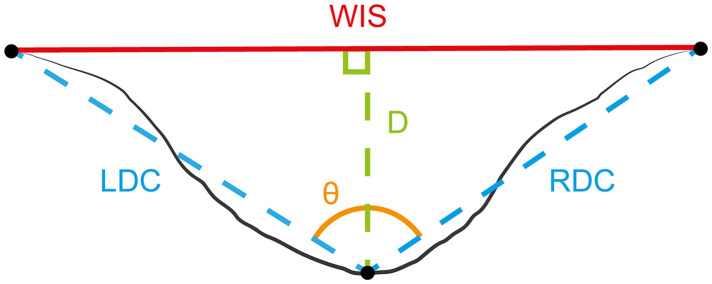
Morphometric measurements taken from the profile of the engravings. WIS: Width of the Incision at the Surface, LDC: Left Depth Convergent, RDC: Right Depth Convergent. Diagram redrawn from Courtenay et al [139].

For each profile, four morphometric measurements were recorded (Fig 3): profile depth (D), width at the incision surface (WIS), profile asymmetry (A) and opening angle (θ). Profile asymmetry, was quantified by reflecting the left side of the incision onto the right and measuring the distance between the two sides, following the method described in Courtenay et al. [139]. Data normality was assessed with the Shapiro-Wilk test. For normally distributed variables, we used standard descriptive statistics (mean, standard deviation) and conducted analysis of variance (ANOVA). For non-Gaussian distributions, we applied robust statistics (median, square root of biweight midvariance) [140–144] and the Kruskal-Wallis test.

The opening angles of the engravings were treated as circular data. A descriptive analysis was carried out to characterize the central tendency as well as the concentration of values around this direction. The presence of a preferential orientation was assessed using Rayleigh’s test [145], which determines whether the angles are uniformly distributed around the circle, or exhibit a dominant direction. To compare mean directions between groups, a generalized Watson-Williams test was used. This test assesses whether several sets of circular data differ significantly in their mean directions. PCA was then performed on metric data after transforming circular variables (see above). Given the relatively small sample sizes for engravings, which can make the calculation of reliable confidence intervals problematic, convex hulls were used to visually represent the spread and boundaries of the data in multivariate space for all engravings PCA.

Elliptic Fourier Analysis (EFA) was then used to analyze the morphological features of different profiles [146–149]. EFA decomposes an outline into a series of trigonometric functions, known as harmonics (*h*), which are described by 4 coefficients, labeled *a, b, c* and *d*. The summation of these harmonics allows for the reconstruction of the original shape or form of profiles [139]. In our study, six harmonics were sufficient to capture more than 98% of the information. Harmonics can then be directly analyzed using multivariate statistical techniques to assess variations and patterns in form (shape + size) or shape (excluding size). For the former, all 4 coefficients of each harmonic are used. However, to remove the impact size has on the coefficients, we use coefficients *a, b* and *c* of the first harmonic to normalize the remaining variables, thus yielding a total of (h × 4) – 3 variables for shape analyses.

Prior to the main analyses, we assessed whether notable allometric relationships existed between the size-normalized harmonic coefficients and engraving size (measured as a function of centroid size). These analyses indicated that shape-size patterns are isometric. Therefore, all subsequent analyses were conducted on shape alone, as described by EFA.

PCA was first performed for the purpose of pattern recognition. For PCA, a univariate analysis was conducted on the first principal component (PC1). The normality of its distribution was assessed using the Shapiro-Wilk test. Based on the results, either an ANOVA or a Kruskal-Wallis test was performed to evaluate the effect of group labels on the distribution of PC1. Finally, a multivariate analysis of variance (MANOVA) [150,151] was conducted on the first two principal components because it accounts for over 95% of the variation. For this purpose, Wilk’s Λ was used as the test statistic, combined with a permutation procedure (n = 999), to assess whether multivariate differences exist between groups.

## 3. Results

### 3.1. Analysis of techniques

#### 3.1.1. Qualitative analysis.

The experimentally worked surfaces (S3 Fig in S1 File) can be grouped into four categories based on their macroscopic characteristics: (1) incisions, (2) impact marks, (3) striations, and (4) those without any visible macroscopic traces.

**Fig 4:**
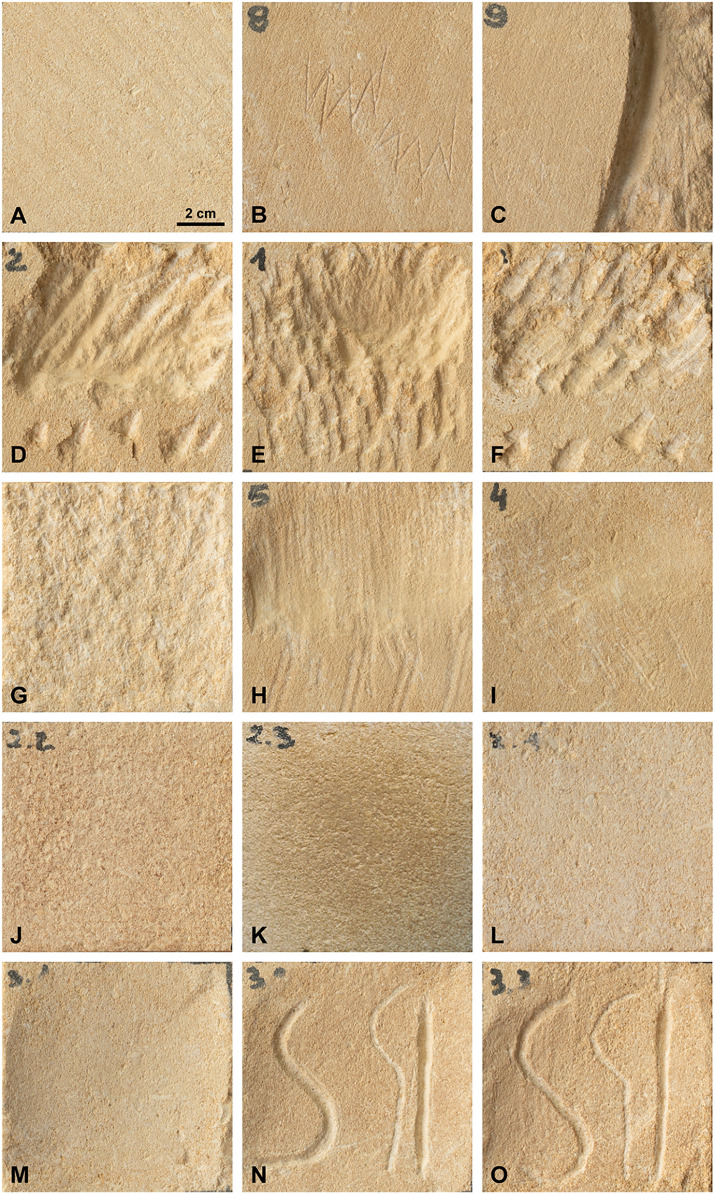
Surfaces resulting from the various techniques tested. **(A)** CS: Control surfaces correspond to unworked areas of the blocks; **(B)** Ems: Engraving with multiple strokes using a blade (Set **C)**; **(C)** PDPB: Pecking in Direct Percussion with a broken Blade + Ems: Engraving with multiple strokes with a pick (Set **I)**; **(D)** PIPP: Pecking in Indirect Percussion with a Pick; **(E)** PDPP: Pecking in Direct Percussion with a Pick; **(F)** PIPB: Pecking in Indirect Percussion with a Blade; **(G)** PDPC: Pecking in Direct Percussion with a Cobble; **(H)** ScS: Scraping with an endScraper; **(I)** ScB: Scraping with a Blade; **(J)** PoS: Polishing with Skin; **(K)** PoHS: Polishing with Humid Sand; **(L)** PoC: Polishing with a Cobble; **(M)** PSc: Pecking in direct percussion with a broken blade and Scraping with an endScraper; **(N)** PScPoE: Pecking in direct percussion with a broken blade, Scraping with an endscraper, Polishing with Skin and Engraving with a blade (Sets D, **E)**; **(O)** PScEPo: Pecking in direct percussion with a broken blade, Scraping with an endscraper, Engraving with a blade (Sets F, G) and Polishing with Skin.

Engraving is the most easily identifiable technique, as it refers both to a specific gesture and to the resulting mark. Both straight and curvilinear engravings display similar morphological characteristics when produced with the same tool and by the same experimenter. Superficial incisions (Fig 4B), produced with single or multiple strokes, are narrow and typically exhibit a V-shaped cross-section, often asymmetrical. Burins tend to produce slightly wider incisions than flakes or blades. Additionally, the preliminary lowering of an edge through pecking facilitated the production of deeper engravings (Fig 4C), characterized by U-shaped or asymmetrical V-shaped cross-sections. This asymmetry is less pronounced when using a pick, as experimenters naturally adopted a (sub-) perpendicular working angle to the surface. Moreover, the material appears crushed along the edges of these deep engravings, giving them a smoothed appearance. Nonetheless, micro-striations can still be observed inside them.

Impact marks are diagnostic of pecking (Fig 4D, E, F, G). Most are sub-centimetric in size, with variable shapes — circular, ellipsoidal, triangular, rectangular — depending on the tool used and the individual’s gesture. Indirect percussion (Fig 4D, F) generally produces larger and more elongated impact marks than direct percussion (Fig 4E, G), particularly when using picks, which was tested with both methods. Within the same percussion mode, however, our dataset does not clearly differentiate between tools based on mark morphology alone. One exception is the broken blade (Fig 4F), which occasionally leaves micro-striations within the impact marks. Of all techniques, pecking removes the most material, creating rough surfaces with alternating peaks and pits. Repeated blows tend to gradually mask these marks and homogenize the surface.

Scraping produces straight, sub-parallel striations (Fig 4H, 4I), the length of which depends on the amplitude of the sculptor’s movement. Although all striations are superficial, those produced with endscrapers (Fig 4H) are more pronounced than those made with unretouched blades (Fig 4I). Worked areas sometimes exhibit slight undulations, caused by the limestone’s natural irregularities, such as embedded small fossils, which cause the tool to skip. However, repeated scraping gradually smooths these irregularities. As with pecking, the cumulative effect of repeated gestures tends to homogenize the surface, often to the extent that striations become no longer visible and the surface appears uniformly smoothed.

The same phenomenon occurs with polishing (Fig 4J,4K,4L). Aside from very superficial circular or straight fine striations, most polished surfaces show no other visible marks, particularly when using tanned skin (Fig 4J), but also with wet sand (Fig 4K). However, these striations appear slightly deeper when a pebble is used (Fig 4L). In addition, detached particles are crushed onto the surface, clogging superficial pores. In our experiments difference in surface coloration were observed depending on the polishing material. Use of a quartz pebble resulted in a whitish hue, probably due to crushing of limestone (or quartz) particles; polishing with wet sand caused sand grains to become embedded in the limestone pores, giving the surface a beige tint. Tanned hide also imparted a beige coloration to the bloc. However, such color variations are unlikely to be reliable indicators in archeological contexts, as original surface residues would most likely no longer be preserved. As for the material used in polishing, tanned hides tend to disintegrate during use, while wet sand proved difficult to handle on a surface inclined at 60°. While these issues posed challenges under our experimental conditions, they do not necessarily reflect prehistoric constraints. Prehistoric artisans may have considered the degradation of certain tools (such as hide) acceptable for such activities and sand could well have been used on both inclined and horizontal surfaces, despite the handling difficulties we observed.

**Fig 5:**
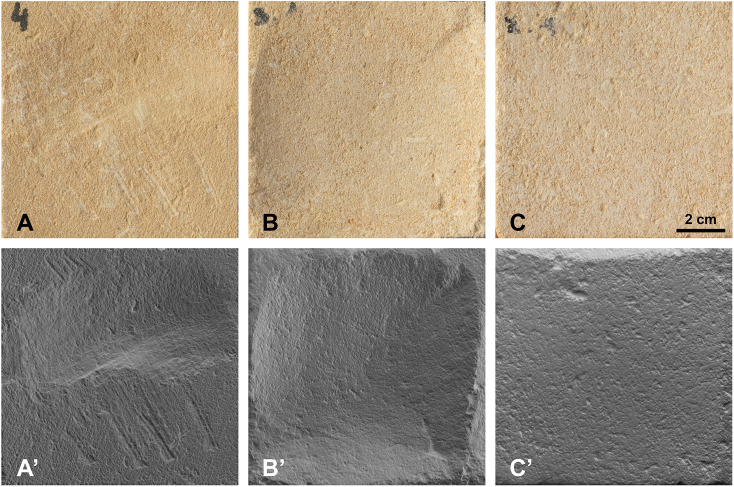
Photographs (A-C) and RTI images (A’-C’) comparing surface modifications produced by (A, A’) scraping with a blade; (B, B’) scraping with an endscraper on a surface previously pecked with a pick; (C, C’) polishing with a cobble. Textural differences between B and C may be difficult to distinguish with the naked eye. RTI images, however, show that larger pores and depressions are preserved on surfaces subjected to polishing alone.

Taken together, the results show that for pecking, scraping, and polishing, repeated gestures can partially obscure or eliminate the characteristic marks of each technique. Pecking traces, however, remain consistently recognizable. As with accumulation of repeated gestures, the superimposition of different techniques often leads to the partial obliteration of earlier marks. For most participants, surfaces that were initially pecked and then scraped retain visible impact marks only at the margins of the worked area, where scraping intensity was lower (Fig 4M). When polishing follows scraping, the already faint scraping striations became barely perceptible or disappeared entirely, again, with residual traces visible only at the margins. In some cases, surfaces that were pecked, then scraped and finally polished become difficult to distinguish visually from surfaces that were only scraped or polished (Fig 5B,5C). In these cases, protruding micro-relief elements are flattened. RTI imaging, however, reveals that more pronounced pores and depressions are preserved on surfaces that we polished only (Fig 5B′,5C′).”

The superimposition of polishing and engraving yielded different outcomes depending on the sequence. Engravings made on a polished surface are visually sharper in part due to the lighter, whitish background of the grooves that increased contrast (Fig 4N). The edges of these engravings are clean and smooth. In contrast, engravings that were subsequently polished appear less distinct (Fig 4O). Polishing stained not only the surface but also the background of the incisions, which visually reduces their contrast with the surrounding surface. Additionally, the edges of these incisions were less uniform, and the engraving themselves appeared shallower. It is likely that the polishing process slightly abraded the raised edges of the incisions, reducing their overall relief.

#### 3.1.2. Quantitative analysis.

**Fig 6:**
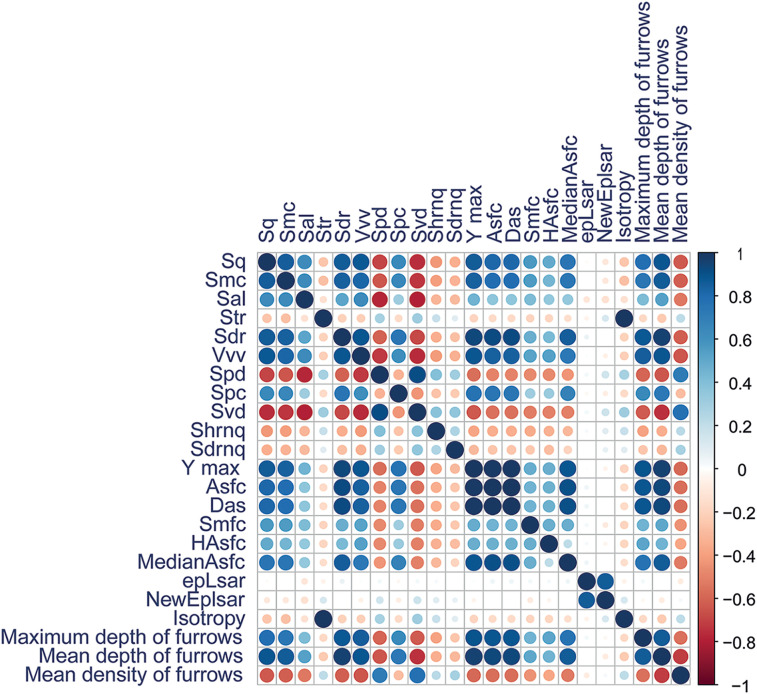
Correlation matrix of roughness parameters retained for the technique analysis.

Among the 29 surface parameters measured, 23 showed statistical differences (Table 4; S1 and S2 Tables in S2 File). The remaining variables are presented in S4 and S5 Figs in S1 File. Among the 23 notable parameters, 10 were not highly correlated and were selected for multivariate analyses (Table 4; Fig 6; S3 Table in S2 File; highly correlated parameters in S4 Fig in S1 File).

**Fig 7:**
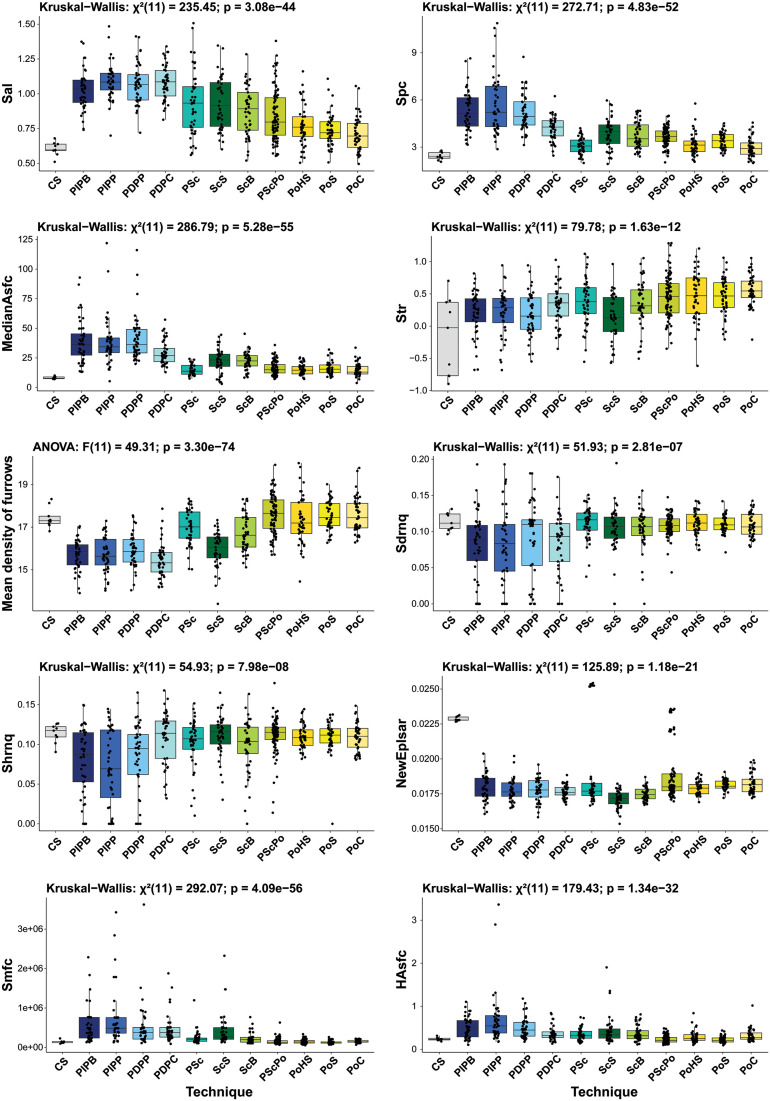
Distribution of selected parameters for the technique analysis. CS: Control Surface; PIPB: Pecking in Indirect Percussion with a Blade; PIPP: Pecking in Indirect Percussion with a Pick; PDPP: Pecking in Direct Percussion with a Pick; PDPC: Pecking in Direct Percussion with a Cobble; PSc: Pecking in direct percussion with a broken blade and Scraping with an endScraper; ScS: Scraping with an endScraper; ScB: Scraping with a Blade; PScPo: Pecking in direct percussion with a broken blade, Scraping with an endscraper and Polishing with Skin; PoHS: Polishing with Humid Sand; PoS: Polishing with Skin; PoC: Polishing with a Cobble.

Control surfaces (CS) (e.g., for Spc, CS: 2.38 ± [2.09, 2.77] 1/mm), exhibit smoothed textures similar to polished surfaces (e.g., for Spc, PoC: 2.90 ± [2.04, 4.28] 1/mm; Fig 7; S4 Table in S2 File). However, isotropy metrics show contrasting trends. CS values for Str are highly dispersed (IQR = 1.14) relative to the tested techniques (mean IQR of other techniques = 0.41), while for NewEplsar, CS data values are tightly clustered (IQR = 0.0002), and display higher median anisotropy (med = 0.0228) than the other techniques (mean med = 0.0178). This greater anisotropy is likely due to uniform sawing of the blocks, which produced uniformly oriented marks.

Three parameters, Sal, Spc, and MedianAsfc, exhibit a decreasing trend from pecking to scraping, and from the latter to polishing (Fig 7; S4 Table in S2 File). This pattern reflects the broader, more spaced impact marks resulting from pecking compared to the finer, more frequent striations produced when scraping or polishing (Sal). Correspondingly, pecking produces more pronounced surface peaks (higher Spc) and greater complexity in surface geometry (higher MedianAsfc).

An increasing gradient, from pecking to polishing, with scraping in between, is observed for the parameters Str, Mean density of furrows, Sdrnq, and Shrnq (Fig 7). This is likely due to more multidirectional movements during polishing, producing more isotropic surfaces (higher Str) and denser furrow networks. Polished and scraped surfaces also show higher micro-relief complexity (Sdrnq, Shrnq), likely due to numerous fine striations captured by high-resolution measurements, whereas pecking creates fewer but deeper marks.

**Fig 8:**
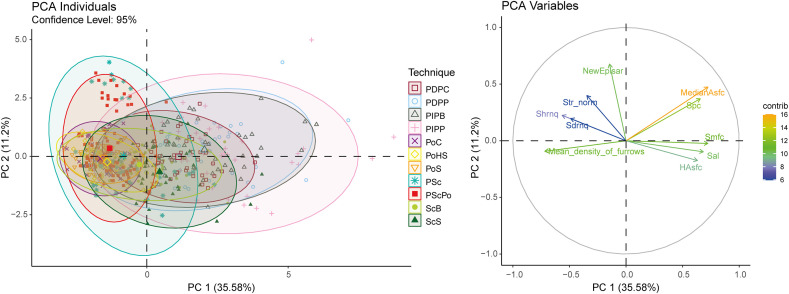
PCA based on selected roughness parameters. CS: Control Surface; PIPB: Pecking in Indirect Percussion with a Blade; PIPP: Pecking in Indirect Percussion with a Pic; PDPP: Pecking in Direct Percussion with a Pick; PDPC: Pecking in Direct Percussion with a Cobble; PSc: Pecking in direct percussion with a broken blade and Scraping with an endScraper; ScS: Scraping with an endScraper; ScB: Scraping with a Blade; PScPo: Pecking in direct percussion with a broken blade, Scraping with an endscraper and Polishing with Skin; PoHS: Polishing with Humid Sand; PoS: Polishing with Skin; PoC: Polishing with a Cobble.

PCA (Fig 8) further support these gradients. While a general separation exists from pecking to scraping to polishing, there is considerable overlap, particularly between scraping and polishing. For example, ScB overlaps nearly the entire polishing data range. Pecking data, regardless of tool, are widely dispersed across both PC1 and PC2 and intersect with scraping and polishing clusters.

Composite techniques also show interesting patterns. Mean density of furrows is higher for combined techniques like PSc (16.97 ± [15.09, 18.21] cm/cm^2^) and PScPo (17.60 ± [15.92, 19.20] cm/cm^2^) than for the individual techniques they contain. For parameters such as Sal, Spc, and MedianAsfc (Fig 7), PSc values (e.g., MedianAsfc: 13.80 ± [9.06, 24.04]) are lower than for scraping alone (e.g., for ScS, MedianAsfc: 23.22 ± [4.39, 42.43]) and more like polishing (e.g., for PoC, MedianAsfc: 13.02 ± [7.13, 25.92]). PSc follows a similar trend (Fig 8), with tighter clustering near polishing data. These results align with qualitative observations: superimposed techniques tend to smooth previous traces and visually resemble polishing.

Dispersion patterns vary considerably across techniques and parameters. For parameters like Spc and MedianAsfc (Fig 7), dispersion is narrower for polished surfaces and combined techniques (e.g., for Spc, PoC: IQR = 0.72 1/mm), and broader for pecking and scraping (e.g., for Spc, PIPP: IQR = 2.57 1/mm). Similarly, pecking exhibits greater variability than other techniques in parameters such as Sdrnq, Shrnq, HAsfc, and Smfc (e.g., for Sdrnq, PIPP: IQR = 0.06; PoS: IQR = 0.02). These patterns are also reflected in the PCA (Fig 8). Superimposed techniques show high variability along PC2 (Fig 8), which is particularly influenced by NewEplsar. This heightened dispersion is largely driven by high values associated with a single novice participant (e.g. PSc max = 0.0254; PScPo max = 0.0236).

Overall, distinguishing the specific tools used remains challenging, as surface parameter values tend to cluster by technique and often show substantial overlap (Fig 7). Tool discrimination is not possible for pecking, where different implements (e.g., PIPB, PDPP) produce largely overlapping results; only PIPP exhibits greater dispersion along both PCA axes (Fig 8).

In contrast, tool-specific differentiation is possible in some cases for scraping and polishing (Fig 8). For scraping, PCA reveals differences between surfaces produced with a blade (ScB) and those produced with a retouched scraper (ScS). Variation along the second PCA axis, structured mainly by NewEplsar and Str parameters related to surface isotropy, indicates greater textural heterogeneity for ScS. This variability is attributable to the irregular morphology and shorter contact length of the retouched edge, which necessitates a higher number of strokes and results in less uniform surface organization. Conversely, ScB produces more anisotropic surfaces, consistent with the linear contact of an unretouched blade edge that promotes parallel striation patterns. Differentiation along the first PCA axis further reflects a denser network of fine striations (Mean density of furrows) for ScB, whereas ScS is characterized by broader surface structures (higher Smfc), more widely spaced patterns (higher Sal), and more pronounced reliefs (higher Hasfc), in agreement with qualitative observations. For polishing, PoC can be distinguished from other conditions by its greater dispersion along the second PCA axis (Fig 8), reflecting the production of both isotropic and anisotropic textures. PoHS and PoS cluster more closely, consistent with qualitative observations that striations are difficult to detect under these conditions. However, PoHS shows greater dispersion along the first PCA axis, particularly in Shrnq and Sdrnq parameters, indicating higher surface texture variability and more pronounced micro-relief compared to polishing with skin. This pattern accords with expectations, given the coarser nature of the sand used in PoHS. While some of these differences are subtle at the macroscopic scale, they become more clearly identifiable through quantitative texture analysis, and in certain cases can be supported by qualitative observations. We therefore emphasize that tool discrimination is technique-dependent and probabilistic, rather than systematic, and is best approached through the combined use of qualitative and quantitative criteria.

The LDA (Table 6; S6 Fig in S1 File; S5 Table in S2 File) yielded an overall classification accuracy of 32.8% ± [28.9–37.0%] with a Kappa coefficient of 0.25, indicating only fair agreement between predicted and observed classes. Although the model performed significantly above chance (*p* < 0.001), discriminative power was generally low. Examination of the confusion matrix (S6 Table in S2 File) shows frequent confusions. While the model correctly distinguishes between broad technique types (pecking, scraping, polishing), it struggles to identify the specific tools used within each category. Within the pecking group, where PDPC, PDPP, PIPB, and PIPP were often misclassified with one another (e.g., PDPP was correctly classified in only 6 instances, while 12 were misclassified as PDPC and 11 as PIPP). Similarly, for PScPo, misclassifications mainly involved other polishing-related classes, likely reflecting its position as the final applied technique. However, given the small and imbalanced sample sizes, these patterns should be interpreted with caution. The limited classification performance reflects the constraints of the dataset rather than shortcomings of the LDA method.

**Table 6:** Summary of LDA statistics for the technique analysis: overall data and by skill level.

	All data	Novice	Intermediate	Expert
Accuracy	0.328	0.405	0.375	0.556
Kappa	0.250	0.331	0.305	0.507
Accuracy Lower	0.289	0.347	0.298	0.457
Accuracy Upper	0.370	0.465	0.457	0.651
Accuracy Null (NIR)	0.247	0.244	0.171	0.176
Accuracy *p*-value	1.23 × 10^−5^	2.12 × 10^−09^	1.66 × 10^−09^	7.47 × 10^-19^

When examined by participant skill level (Table 6; S6 Fig in S1 File; S5 and S6 Tables in S2 File), classification accuracy increased from novices (40.5%) to experts (55.6%). However, the corresponding Kappa coefficients (0.33–0.51) indicate only modest agreement, showing that classification remains weak despite being above chance. In particular, the apparent improvement for experts should be interpreted with caution, as this “group” consists of a single individual, despite multiple acquisitions (n = 108).

**Fig 9:**
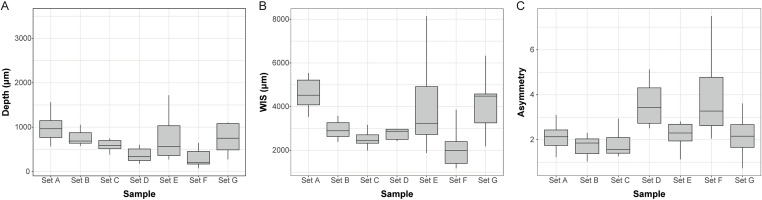
Distribution of Depth, WIS, and A in the superficial engraving analysis. Set A: Engravings produced with a burin using multiple strokes, Set B: Engravings produced with a flake using multiple strokes, Set C: Engravings produced with a blade using multiple strokes, Set D: Pecking + Scraping + Polishing + Engravings produced with a blade using single stroke, Set E: Pecking + Scraping + Polishing + Engravings produced with a blade using multiple stroke, Set F: Pecking + Scraping + Engravings produced with a blade using single stroke + Polishing, Set G: Pecking + Scraping + Engravings produced with a blade using multiple strokes + Polishing.

In the analysis of superficial engravings (S7 Fig in S1 File), three parameters, depth, width in surface (WIS), and asymmetry were found to present differences between groups (S7 Table in S2 File). As expected, Sets D and F are shallower than those produced with multiple strokes (e.g., Set F: 300.24 ± [185.63, 422.28] µm; Set A: 978.72 ± [815.52, 1146.57] µm; Fig 9A; S8 Table in S2 File). The single stroke engravings are also more asymmetric (e.g., Set F: 3.91 ± [2.95, 4.95]; Set A: 2.14 ± [1.78, 2.45]; Fig 9B). Repeating the engraving gestures tend to reduce asymmetry regardless of the tool used.

Regarding WIS (Fig 9C), the narrower engravings are those in Set F, where single stroke incisions were made on a pecked and scraped surface, and then polished (Set F: 2213.85 ± [1638.25, 2890.60] µm). In contrast when polishing precedes the engraving, WIS values are comparable to those of other sets (e.g., Set D: 2909.04 ± [2340.73, 3510.03] µm; Set B: 2953.58 ± [2697.36, 3209.79] µm).

Tool type also influences depth and width. Burin (Set A) produced deeper incisions than flakes (Set B) and blades (Sets C to G), which yielded comparable results (e.g., Set A: 978.72 ± [815.52, 1146.57] µm; Set B: 797.64 ± [658.47, 968.82] µm; Set C: 635.48 ± [506.39, 790.35] µm). Surface preparation (pecked, scraped and eventually polished surface) appears to have little impact on depth. However, burins also created notably wider incisions on initially flat, non-modified surfaces (e.g., Set A: 4604.93 ± [4178.25, 5005.68] µm; Set B: 2953.58 ± [2697.35, 3209.79] µm). When multiple-stroke incisions were made on prepared surfaces (Sets E and G), their width approached that of engravings made with burins. For example, Set G (polished after engraving) and Set E (no polishing) yielded similarly broad incisions (Set G: 4316.31 ± [3410.08, 5252.48] µm; Set E: 3985.78 ± [2931.07, 5107.79] µm). Notably, polishing after engraving (Set G) produced wider incisions than when no polishing followed (Set E), likely because polishing removed loosely attached limestone particles and slightly widened the incision edges

The opening angles of the engravings show a statistically preferred orientation (Rayleigh *p* < 0.003; S8 Fig in S1 File; S9 Table in S2 File) both within individual set and across the dataset. While the angles are relatively close overall, two distinct groups emerge: the multiple-stroke engravings (Sets A, B, C, E, and G) tend to be oriented between 121° and 137°, whereas single-stroke engravings (Sets D and F) cluster around 150°.

**Fig 10:**
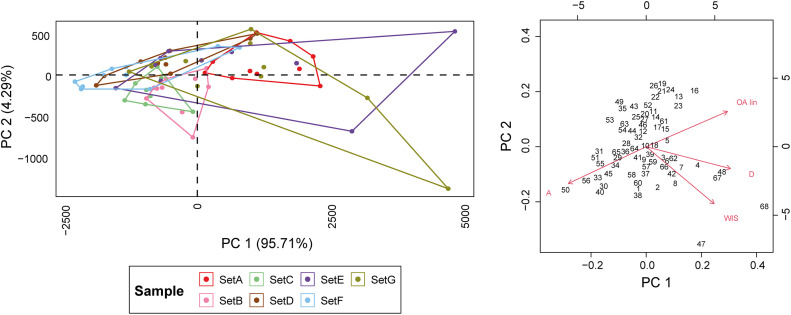
PCA based on metric statistics for the superficial engraving analysis.

**Fig 11:**
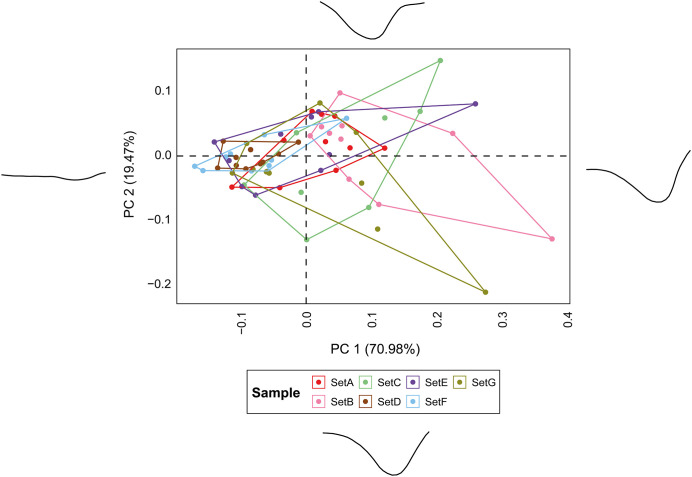
PCA based on EFA of superficial engravings. Extreme shape changes are visualised across each of the corresponding PC scores. Set A: Engravings produced with a burin using multiple strokes, Set B: Engravings produced with a flake using multiple strokes, Set C: Engravings produced with a blade using multiple strokes, Set D: Pecking + Scraping + Polishing + Engravings produced with a blade using single stroke, Set E: Pecking + Scraping + Polishing + Engravings produced with a blade using multiple stroke, Set F: Pecking + Scraping + Engravings produced with a blade using single stroke + Polishing, Set G: Pecking + Scraping + Engravings produced with a blade using multiple stroke + Polishing.

The PCA performed on the metric measurements reveal substantial overlap between the different sets (Fig 10; S10 Table in S2 File), although the first principal component (PC1) reveals statistical differences across groups (Kruskal-Wallis: χ² = 29.569, *p* = 4.74 × 10^−5^). Among the sets, E and G are the most distinguishable, showing broader dispersions along both PC1 and PC2, reflecting their greater WIS and depth values. Sets D and F are nearly indistinguishable due to their close overlap. Engravings made with a burin (Set A) stand apart from those produced using a flake or a blade, except when surface preparation is involved, as in Sets E and G. Shape variation, assessed through PCA of EFA descriptors, does not improve discrimination among sets (Fig 11; S11 Table in S2 File). However, it is worth noting that Sets B, C, E, and G exhibit higher morphological variability, ranging from open to closed cross-sections, often with a plateau on either the left or right edge.

For the widest engravings, depth, WIS, and asymmetry do not differ considerably between those produced by pecking followed by incision with either a burin (Set H) or a pick (Set I; S7 and S8 Tables in S2 File; S9 Fig in S1 File). Similarly, the PCA based on EFA descriptors shows no notable separation between the two sets (ANOVA: *F* = 1.18, *p* = 0.29). A preferred orientation is detected in the opening angles of both sets (Rayleigh: *p* < 0.003; S8 Fig in S1 File; S9 Table in S2 File), yet no notable differences emerge between them (Watson-Williams test: *P*_*g*_ = 3.2, *p* = 0.074). Engravings in Set H are oriented around 91°, and those in Set I around 98°, in both cases with opening angles lower than those observed for the superficial engravings (136°; S9 Table in S2 File).

### 3.2. Analysis of expertise

#### 3.2.1. Qualitative analysis.

Observation of the experimentally modified surfaces (S3 Fig in S1 File) reveals few consistent differences between skill levels. Inter-individual variability, combined with the limited number of experimenters per group (one or two per level), outweigh any clear effect of prior expertise under the present experimental conditions. Because experience categories were not based on performance-based criteria and because group sizes were small (including only one expert), observations related to ‘skill level’ should be regarded as exploratory and sample-dependent rather than generalizable.

**Fig 12:**
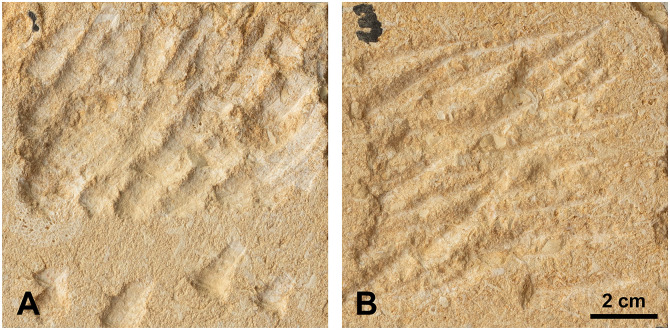
Comparison of indirect percussion pecking marks produced with a broken blade by (A) the expert and (B) a novice. The two experimenters used different active parts: the novice struck with one of the two angles of the break, while the expert used the entire fractured edge.

For pecking, some distinctions related to skill level do emerge. The expert produced highly regular, homogeneous, and controlled marks, whereas some novices left disorganized traces with inconsistent orientations and directions. For example, with the broken blade, the shape of the impact marks varied markedly (Fig 12), reflecting unconventional tool handling by a beginner (striking with one of the angles of the break rather than the entire fractured edge). Tool efficiency appears linked to individual proficiency. For instance, the novices and one of the intermediate participants removed more material using a pebble for direct percussion on a flat surface than when using a pick. In contrast, the expert sculptor achieved greater efficiency with a pick, likely due to its resemblance to modern tools that he regularly uses such as chisels. Surface regularization also varies substantially between individuals, showing no consistent pattern based on tool type.

For scraping, although the expert generally produced smoother surfaces with faint or no striations, there was no clear gradient among novices and intermediates on the one hand, and the professional sculptor on the other hand. Some gestures such as placing the left hand over the right to apply more pressure with the endscraper, or switching hands when fatigue, were observed across different skill levels.

Similarly, polishing differences were minimal. Some of the most finely polished surfaces were created both by expert and novices.

For engraving, differences are more evident. The expert executed single-stroke curvilinear engravings with greater force and tool control. For multiple-stroke engravings, the engraver first incised a shallow guiding line which was retraced multiple times. This resulted in deeper, wider engravings with smooth, regular edges, visibly distinct from those produced by the other participants, whose engravings were generally shallower and more irregular. The ability to follow a previous incision accurately also varied: some experimenters succeeded in retracing along the same line, while others struggled, producing sequences of closely spaced, superficial marks or engravings with uneven cross-sections. These difficulties were not sporadic or spatially clustered on specific blocks, but instead were consistently observed for the same experimenters across different working areas and experimental conditions.

Regarding the superimposition of techniques, edges where not all techniques were applied uniformly provide valuable information, particularly when traces of pecking can be observed. During the pecking–scraping sequence, the expert produces a depression with a smooth surface, whereas the other experimenters create less pronounced depressions, whose surfaces exhibit undulations resulting from the scraping following the volumes imposed by the pecking marks. The expert is thus able to completely level the surface. Furthermore, the successive application of polishing is not sufficient to smooth out these undulations in the novices and intermediate participants.

#### 3.2.1. Quantitative analysis.

**Fig 13:**
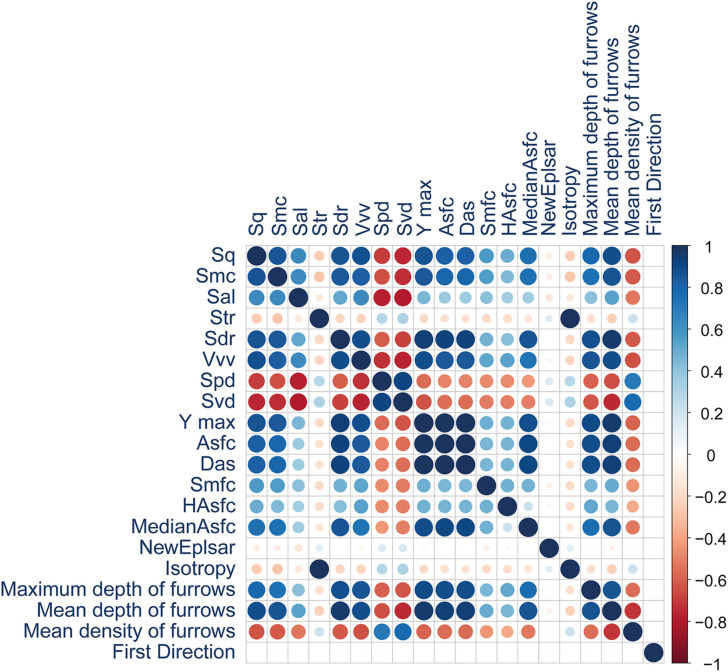
Correlation matrix of roughness parameters obtained for the expertise analysis.

Among the 29 measured parameters, 20 showed statistical differences (Table 4; S12 and S13 Tables in S2 File; non-significant parameters in S10 and S11 Figs in S1 File). Among these, 8 were not highly correlated (Table 4; Fig 13; S14 Table in S2 File; highly correlated parameters in S10 Fig in S1 File) and were retained for the remainder of the study.

**Fig 14:**
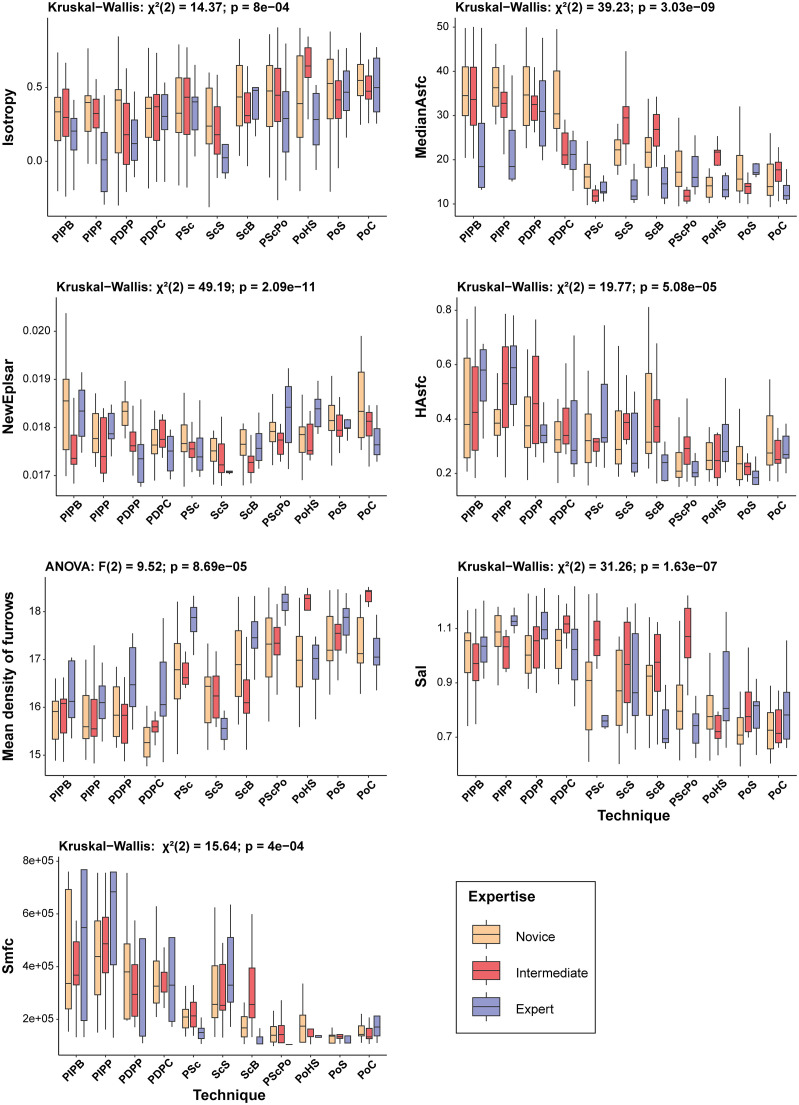
Distribution of retained linear parameters for the expertise analysis. PIPB: Pecking in Indirect Percussion with a Blade; PIPP: Pecking in Indirect Percussion with a Pick; PDPP: Pecking in Direct Percussion with a Pick; PDPC: Pecking in Direct Percussion with a Cobble; PSc: Pecking in direct percussion with a broken blade and Scraping with an endScraper; ScS: Scraping with an endScraper; ScB: Scraping with a Blade; PScPo: Pecking in direct percussion with a broken blade, Scraping with an endscraper and Polishing with Skin; PoHS: Polishing with Humid Sand; PoS: Polishing with Skin; PoC: Polishing with a Cobble.

Depending on the parameters studied, different patterns can be observed for a given technique (Fig 14; S15 Table in S2 File). Isotropy generally decreases with increasing expertise (e.g., for PIPP, Novice: 0.35 ± [−0.33, 0.75], Intermediate: 0.32 ± [−0.46, 0.79], Expert: −0.07 ± [−0.60, 0.43]). The lower isotropy in the expert’s data reflects minimal variation in movement directions, confirming the consistent gestures observed on the pecked surfaces. MedianAsfc also exhibits a decreasing trend for pecking, from novices to expert (e.g., for PIPP, Novice: 39.53 ± [28.78, 81.05], Intermediate: 35.78 ± [23.52, 98.46], Expert: 17.20 ± [7.27, 38.76]). For scraping, the trend is less clear, with intermediates showing higher values (e.g., for ScS, Novice: 22.25 ± [12.63, 32.56], Intermediate: 29.46 ± [19.05, 43.82], Expert: 6.82 ± [3.32, 17.62]). Polishing data reveal no clear skill-level distinctions (e.g., for PoC, Novice: 12.76 ± [5.87, 28.67], Intermediate: 17.71 ± [11.06, 22.74], Expert: 11.87 ± [10.18, 17.34]). MedianAsfc values are generally lower for the expert, indicating less complex surfaces. This, again, reflects better gesture control, resulting in more regular surfaces compared to the other participants, who inadvertently increase surface complexity.

Mean density of furrows shows an increasing trend from novices to the expert (e.g., for PDPC, Novice: 15.00 ± [14.19, 15.89] cm/cm^2^, Intermediate: 15.63 ± [15.13, 16.34] cm/cm^2^, Expert: 16.35 ± [15.04, 17.75] cm/cm^2^), except for ScS (Fig 14). Higher values in the expert likely indicate more closely spaced furrows, reflecting a more refined surface treatment.

For Smfc, the expert shows a wide range of values in pecking (e.g., for PIBP, IQR = 562,409.33 µm²; Fig 14), while values are more consistent for other techniques and superimpositions (e.g., for PScPo, IQR = 123.50 µm²). Again, ScS stands out with much higher dispersion (e.g., Expert: IQR = 245,466.23 µm²) than that of ScB, the other scraping technique (e.g., Expert: IQR = 26,252.55 µm²). For Sal, the pattern is reversed: the expert’s values are more tightly clustered for pecking (e.g., for PIPP, IQR = 0.03 mm) than for other techniques (e.g., for PoHS, IQR = 0.25 mm). Compared to other skill levels, the expert shows higher Sal values for pecking and lower ones for scraping and superimpositions. This indicates that the expert produces broader and more regular pecking marks and finer and more tightly spaced scraping striations than other participants.

**Fig 15:**
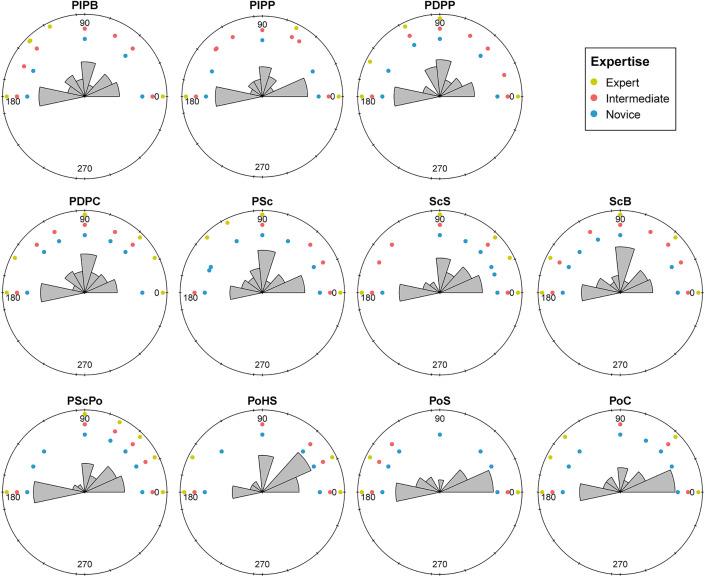
Distribution of retained circular parameters for the expertise analysis. PIPB: Pecking in Indirect Percussion with a Blade; PIPP: Pecking in Indirect Percussion with a Pick; PDPP: Pecking in Direct Percussion with a Pick; PDPC: Pecking in Direct Percussion with a Cobble; PSc: Pecking in direct percussion with a broken blade and Scraping with an endScraper; ScS: Scraping with an endScraper; ScB: Scraping with a Blade; PScPo: Pecking in direct percussion with a broken blade, Scraping with an endscraper and Polishing with Skin; PoHS: Polishing with Humid Sand; PoS: Polishing with Skin; PoC: Polishing with a Cobble.

Although isotropy values are generally high (mostly above 30%; S15 Table in S2 File) and do not indicate a strongly marked texture direction [152], the expert’s surfaces still exhibit a more preferred orientation (Fig 15; S16 Table in S2 File), implying lower angular dispersion than those of novices and intermediates. This suggests that despite generally high isotropy, the expert tends to produce slightly more directional textures, likely due to more repetitive or controlled gestures.

Regarding technique superimpositions, the expert again stands out with much more tightly clustered values for Sal and Smfc, respectively with PSc (e.g., Novice: IQR = 0.27 mm, Expert: IQR = 0.12 mm) and PScPo (e.g., Novice: IQR = 61,879.72 µm², Expert: IQR = 123.50 µm²; Fig 14).

The cross-validated LDA classifications for the expertise analysis show varying performance depending on the technique tested (Table 7; S12 Fig in S1 File; S17 and S18 Tables in S2 File). PScPo reaches the highest agreement with a Kappa of 0.648, approaching substantial agreement. PDPP (κ = 0.542), PoHS (κ = 0.516), ScS (κ = 0.507), and PDPC (κ = 0.414) show moderate agreement. ScB (κ = 0.483), PoC (κ = 0.405), PIPB (κ = 0.349), and PSc (κ = 0.344) achieve fair agreement. PIPP (κ = 0.031) and PoS (κ = 0.125) indicate weak discrimination. Confusion matrices (S18 Table in S2 File) confirm these results: PScPo and PDPP show higher rates of correct classification across expertise levels, whereas PIPP and PoS are frequently misclassified. These results indicate that the model’s ability to distinguish skill levels varies across techniques, with for example pecking appearing among both the best (PDPP, PScPo) and worst (PIPP) performing classifications.

**Table 7:** Summary of LDA statistics by technique for the expertise analysis.

	PDPC	PDPP	PIPB	PIPP	PoC	PoHS	PoS	PSc	PScPo	ScB	ScS
Accuracy	0.667	0.711	0.578	0.386	0.667	0.733	0.533	0.644	0.800	0.667	0.689
Kappa	0.414	0.542	0.349	0.031	0.405	0.516	0.125	0.344	0.648	0.483	0.507
Accuracy Lower	0.510	0.557	0.422	0.244	0.510	0.581	0.379	0.488	0.702	0.510	0.534
Accuracy Upper	0.800	0.836	0.723	0.545	0.800	0.854	0.683	0.781	0.877	0.800	0.818
Accuracy Null	0.578	0.467	0.511	0.432	0.600	0.622	0.667	0.644	0.578	0.422	0.489
Accuracy *p*-value	0.145	0.001	0.228	0.776	0.225	0.081	0.978	0.568	7 × 10^−06^	0.001	0.005

## 4. Discussion

This study provides the first integrated experimental framework for investigating the production of Paleolithic *bas-reliefs*. Our results show that these works must be understood not only as symbolic creations but also as technical productions that emerged from complex operational sequences. These sequences (surface preparation, removal of material to obtain a preform, silhouette outlining, smoothing, and detailing [78]) connect *bas-reliefs* to other domains of prehistoric technology, particularly lithic production, since stone tools were essential to carving.

Producing a *bas-relief* required technical knowledge, cognitive planning, and sustained, often intense engagement with the worked surface, particularly when multiple techniques were combined. Although individual operations are technically accessible even to novices, our experiments show that the cumulative time required to apply successive techniques increases rapidly, even on small standardized surfaces (10 × 10 cm). Extending such sequences over the much larger surfaces observed in archaeological contexts would therefore have entailed a substantial time investment. Nevertheless, the assumption that bas-relief production necessarily involved higher labor costs than other graphic practices deserves critical re-examination. A coarse sculpture executed in soft limestone could, in some cases, be completed more rapidly than a polychrome painting, which requires pigment acquisition and preparation, surface preparation, and time devoted to complex shading [e.g., 72]. From this perspective, the importance of bas-reliefs may lie less in absolute labor costs than in the conceptualization and embodiment of complex visual conventions and symbolic intentions, potentially corresponding to what Marcel Mauss described as ‘religious efficacy’ [153].

A further dimension concerns the level of expertise involved in these productions. The identification of novices and experts in the archaeological record implies systems of knowledge transmission and potentially individuals dedicated wholly or partly to these activities. This raises questions about the division of labor within the group and the social status attributed to individuals engaged in these practices, potentially reflecting a certain social hierarchy [67,68,99,154].

The acquisition of both technical and graphic skills relied on the transfer of knowledge, gestures, and shared conventions from experienced practitioners to novices [e.g., 86,92,94,97,98,154]. Such learning could have occurred formally, through structured training, or informally, via observation and imitation, as seen in other primates. Investigating the materials and supports used by apprentices, documented to differ from those of expert engravers [96,97], may reveal traces of this learning process. A better understanding of these learning dynamics could thus clarify the variety of marks observed in parietal sculpture and, importantly, enable the differentiation of their makers.

Until now, the determination of techniques and tools for sculpture had relied solely on visual observations, and the role of expertise had not yet been investigated. Here, for the first time, we present a combined qualitative and quantitative traceological documentation of experimental parietal sculptures, designed to test hypotheses previously formulated in the literature. From a methodological standpoint, the approach developed in this study allows the extraction of quantitative data from 3D scanned surfaces, enabling detailed analyses of texture roughness and engraving profiles, and thereby reducing some of the subjective biases inherent in purely qualitative descriptions [155–157].

Although the experimental series focuses on controlled conditions and relatively simple forms, the technical gestures documented here correspond to those involved in the production of more complex figurative compositions and decorative motifs. The figurative dimension of Paleolithic parietal sculpture is therefore not solely an iconographic issue, but also a technical one, as the creation of complex images requires the coordinated deployment of multiple gestures, tools, and operational steps. Our results highlight how these gestures leave measurable traces on worked surfaces and structure the organization of labor during the carving process. However, future research will need to extend this framework to experimentally produced figurative compositions in order to evaluate how increasing compositional complexity affects gesture sequencing, trace variability, and the overall investment of time and effort involved in the production of parietal sculptures.

The experimental corpus generated in this study provides a comprehensive reference for the gestures, techniques, and tools involved in Paleolithic sculpture. By systematically documenting both the technical sequences and the resulting traces on standardized limestone surfaces, this corpus establishes a controlled reference framework. Such a dataset enables direct comparisons with archaeological examples, facilitating the identification of specific techniques, patterns of tool use, and the potential level of expertise exhibited by prehistoric artisans. While environmental and taphonomic processes may have altered archaeological marks over time, and the properties of irregular limestone surfaces (e.g., porosity, fossil density) can vary even over very short distances [e.g., 62, 63, 85, 157–165], this experimental framework serves as a valuable benchmark for testing hypotheses and interpreting the prehistoric record.

Focusing on the technical analysis, our results demonstrate that qualitative analysis allows for the identification of marks specific to certain techniques. For instance, percussion marks are systematically associated with pecking, while incisions are only observed in the context of engraving. Conversely, other traces are shared across multiple techniques and therefore cannot be considered discriminative. This is the case for striations, which appear in both scraping and polishing. While it can sometimes be difficult to distinguish between scraping and polishing by visual inspection alone, tools such as RTI or quantitative surface analyses enable more precise identification. However, quantitative analysis emphasizes that a clear-cut separation between techniques is unrealistic, as a continuum of surface modifications is observed. A gradual increase in several surface parameters is apparent from polishing to pecking, with the latter showing broader impact marks (Sal) and more pronounced surface reliefs (Spc), resulting in a more complex overall texture (MedianAsfc). Conversely, isotropy (Str), striations (Mean density of furrows), and micro-reliefs (Sdrnq, Shrnq) increase from pecking to scraping and then to polishing. Thus, the contribution of the quantitative approach here lies less about identifying employed techniques and more about documenting intra-technique variability related to the substrate and the sculptors, whereas qualitative analysis alone risks oversimplifying and reducing the diversity of techniques.

Furthermore, the repetition of gestures, regardless of the technique, appears to have nonlinear effects in both qualitative and quantitative analyses: beyond a certain threshold, the surface microtopography becomes homogeneous while altering previous traces. The same applies when techniques are superimposed, as this modifies the visual appearance of the surfaces by erasing or obscuring previous marks, and it also affects the measured surface parameters, whose values tend to converge toward those typical of polishing, even when polishing itself was not applied. While qualitative observations do not always allow such successions to be detected, quantitative analyses, through the combined use of surface parameters, present a more promising means of identifying such technical sequences. For engravings, quantitative measurements can further complement qualitative observations by indicating the number of strokes used: shallow depth combined with high asymmetry is indeed characteristic of a single-stroke engraving. These results shed light on the complexity of the sculpting process and open perspectives for the study of archaeological material.

In addition, qualitatively, some marks can securely be attributed to specific tools. For instance, pecking with a pick tends to leave elongated, comet-shaped marks (especially in indirect percussion), whereas the use of a pebble produces more rounded impact marks. In other cases, within the same technique, marks produced by a given tool exhibit significant variability between experimenters (e.g., use of a broken blade for pecking), making it difficult to make reliable inferences. From a quantitative perspective, the substantial overlap observed between modalities further highlights the challenge inherent to inferring the tool used from surface roughness data alone.

Our results also demonstrate that inferring the sculptor’s expertise is possible for some–but not all– modalities, while based on a limited number of experimenters. From a qualitative standpoint, identifying different skill levels is easier for pecking and engraving than any other technique. For pecking, while some novices produce irregular and disorganized marks, the expert, by contrast, generates highly regular and controlled traces, resulting in homogeneous surfaces. The expert may even carry out preliminary preparations, akin to a guide directing the engraving. For engravings made with multiple strokes, some novices struggled to retrace the same line, resulting in several closely spaced incisions. This pattern has also been observed in previous studies [96]. The expert’s engravings also appear visually deeper and wider, with smooth and regular edges, compared to those of the other participants.

Similar observations can be drawn from quantitative analyses, which can further be extended to include scraping and polishing. Overall, gradients from novices to the expert indicate improvements in gesture consistency and orientation (decreased Isotropy and First direction dispersion), a decrease in surface complexity (MedianAsfc) associated with increased motor control as well as finer texture processing (increased Mean density of furrows). For pecking, the expert also produces broader and more regular impact marks than other experimenters (higher Sal), highlighting his dexterity.

These results demonstrate that expertise is perceptible to some degree both in visible morphology and in measured microtopography. Moreover, certain techniques are more informative than others for assessing skill levels. Pecking and engraving, which require greater mastery and control, clearly differentiate skill levels, whereas scraping and polishing are easier to execute and show little variation according to operator experience.

Building on these observations, future research could explore whether our findings hold with a larger number of experts and whether inter-individual differences can help identify a given artisan’s technical signature. Nonetheless, strictly attributing and differentiating skill levels remains challenging [e.g., 166–171]. While some theories suggest that excellence, here represented by the “expert” level, can only be reached after approximately ten years of daily practice [172,173], as is the case for the expert in our experiment, it is clear that sculptors’ abilities exist along a spectrum from baseline to expert proficiency. Technical mastery develops along a continuum that is not necessarily linear or uniform, meaning that skill level classifications may appear somewhat arbitrary and do not fully capture the complexity of reality.

What’s more, in our experiments, some novices, although inexperienced in sculpting, had prior experience with lithic tools and showed results closer to intermediates or even the expert. Similarly, intermediate participants may vary depending on their current practice: some continue to train regularly, while others have occasional or discontinued practice. A lack of consistent practice can affect gestural precision and technical mastery. These considerations highlight the need to refine skill level classifications by considering the complexity and variability of individual experience and practice.

Robust evaluation of expertise effects would require larger participant samples and explicit performance-based criteria (or blind classification tests) specifically designed to quantify technical proficiency and efficiency.

A productive avenue for future research will be to move beyond general estimates of labor investment and investigate how combinations of technical gestures are organized across sculpted surfaces. This could be achieved by identifying distinct technical zones within archaeological bas-reliefs—potentially reflecting different moments of execution, compositional strategies, or degrees of technical control—recording their surface textures, and comparing them with experimentally derived reference datasets. Such an approach would make it possible to evaluate how technical proficiency, gesture sequencing, and compositional complexity interact in the production of parietal sculptures. Expanding the experimental framework to include figurative compositions will be essential to assess how the increasing complexity of motifs affects the traces produced by tools and the labor investment required. In turn, this combined approach may also provide a framework for evaluating whether differences in technical proficiency and levels of expertise can be identified in archaeological contexts.

## Conclusion

This work provides new insights into the technical and cognitive complexity of Paleolithic parietal sculpture through the combined use of experimentation, qualitative observation, and quantitative surface analysis. By systematically documenting the traces left by different tools, techniques, and levels of expertise, we established diagnostic criteria that can be applied to archaeological *bas-reliefs*. Our results confirm that certain techniques, such as pecking and engraving, leave clear and distinctive traces, whereas others, such as scraping and polishing, often produce overlapping signatures, particularly when gestures are repeated or superimposed. Engraving proved especially informative: measurable differences in depth, symmetry, and width reflect tool choice, stroke number, and sequence of operations. Expertise is often visible in both qualitative and quantitative data, with experts producing more regular, efficient, and isotropic surfaces. These findings suggest that technical mastery is best understood as a continuum rather than a series of discrete categories. The complementarity of qualitative and quantitative approaches is essential. Visual assessment remains indispensable for recognizing and interpreting reliefs, while quantitative parameters provide objective measures that support or refine qualitatively-derived hypotheses. Together, they make it possible to help reconstruct operational sequences and detect subtle evidence of planning, skill, and learning. Beyond methodological advances, these results have wider anthropological implications. They highlight the role of apprenticeship, specialization, and open the perspective of investigating division of labor in Upper Paleolithic groups, while underscoring the substantial investment of time and expertise required to produce large sculptural works. Such practices imply that art was not a marginal activity but a socially embedded one, contributing to the circulation of knowledge, the negotiation of identity, and the symbolic life of hunter-gatherer societies. Applying this experimental framework to archaeological contexts will allow us to move beyond stylistic or iconographic analyses and toward a more comprehensive understanding of Paleolithic *bas-reliefs* as technical, social, and symbolic productions integrated into the fabric of prehistoric lifeways.

## Supporting information

S1 File Text and figures. (PDF)

S2 File Tables. (PDF)

S3 File R scripts. R scripts used for statistical analyses and figure generation are available online at the GitHub repository: https://github.com/EmBrochard/Sculpture-techniques-expertise_Statistics
(PDF)
