# Supplementary material for: Carved in stone: Experimental criteria for identifying Paleolithic bas-relief production techniques and sculptors’ expertise
Source: PLoS One. 2026 Apr 1;21(4):e0346099. doi: 10.1371/journal.pone.0346099 (PMC13042861; doi:10.1371/journal.pone.0346099)
Supplement: S1 File — (PDF) [file pone.0346099.s001.pdf]

Supplementary File 1: Text and Figures

Contents

1. Supplementary Text 2

S1 Text. Multiscale Surface Roughness Analysis. 2

2. Supplementary Figures 4

S1 Fig. Experimental blocks before and after experimentation. 4

S2 Fig. Experimental modality square digitally subdivided into nine non-overlapping 10 × 10 mm sub-areas. 5

S3 Fig. Experimental blocks of phases 1 and 2 according to levels of expertise. 6

S4 Fig. Boxplots of non-significant and highly correlated parameters for technique analysis. 8

S5 Fig. Rose diagram of non-significant angular parameters in the technique analysis. 10

S6 Fig. CVA plots for the technique analysis, with overall data and by skill level. 11

S7 Fig. Representative engraving profiles for each set. 12

S8 Fig. Distribution of opening angles by engraved set. 13

S9 Fig. Distribution of Depth, WIS, and A in the deep engraving analysis. 14

S10 Fig. Boxplots of non-significant and highly correlated linear parameters in the expertise analysis. 15

S11 Fig. Rose diagram of non-significant circular parameters in the expertise analysis. 17

S12 Fig. CVA plots by technique for the expertise analysis. 20

S1 Text. Multiscale Surface Roughness Analysis.

A single surface can be studied at three levels, equivalent to different wavelength ranges [1]. The primary form of the object corresponds to the lower frequencies. Surface waviness has intermediate wavelengths and documents the surface reliefs carried by the object. Finally, the highest frequencies are associated with micro-roughness present precisely on these reliefs. Depending on the purpose of the study, the scale of resolution required will not be the same, and *a fortiori* the surface treatment. For instance, the primary shape of the surface can be removed by applying a polynomial, the order of which depends on the object or material being studied: 2nd degree [e.g., 2–5], 3rd degree [e.g.,6–8], 5th degree [e.g., 9,10], etc. When surface micro-roughness is the point of interest, then waviness is also removed by applying a Gaussian filter, the value of which fluctuates from study to study [e.g., 1,11–13].

Here, we are interested in the traces produced by technical gestures. The focus is on surface waviness, which the simple primary form does not reveal and which the microscopic scale obliterates. As the pecking and scraping gestures modified the object's primary form, a polynomial of third order was applied to remove the overall shape of each sub-area. This choice followed several polynomial tests: visual inspection showed that a 3rd degree polynomial minimized surface deformation.

References

1. Le Goïc G, Bigerelle M, Samper S, Favrelière H, Pillet M. Multiscale roughness analysis of engineering surfaces: A comparison of methods for the investigation of functional correlations. Mech Syst Signal Process. 2016;66–67: 437–457. doi:10.1016/j.ymssp.2015.05.029.

2. Calandra I, Schulz E, Pinnow M, Krohn S, Kaiser TM. Teasing apart the contributions of hard dietary items on 3D dental microtextures in primates. J Hum Evol. 2012;63: 85–98. doi:10.1016/j.jhevol.2012.05.001.

3. Schulz E, Calandra I, Kaiser TM. Feeding ecology and chewing mechanics in hoofed mammals: 3D tribology of enamel wear. Wear. 2013;300: 169–179. doi:10.1016/j.wear.2013.01.115.

4. Martisius NL, Sidéra I, Grote MN, Steele TE, McPherron SP, Schulz-Kornas E. Time wears on: Assessing how bone wears using 3D surface texture analysis. PLOS ONE. 2018;13: e0206078. doi:10.1371/journal.pone.0206078.

5. Zupancich A, Cristiani E. Functional analysis of sandstone ground stone tools: arguments for a qualitative and quantitative synergetic approach. Sci Rep. 2020;10: 15740. doi:10.1038/s41598-020-72276-0.

6. d’Errico F, Backwell LR, Wadley L, Geis L, Queffelec A, Banks WE, et al. Technological and functional analysis of 80-60 ka bone wedges from Sibudu (KwaZulu-Natal, South Africa). Sci Rep. 2022;12, 16270. doi:10.1038/s41598-022-20680-z.

7. Geis L, d’Errico F, Jordan FM, Brenet M, Queffelec A. Multiproxy analysis of Upper Palaeolithic lustrous gravels supports their anthropogenic use. PLOS ONE. 2023;18: e0291552. doi:10.1371/journal.pone.0291552.

8. Rigaud S, Evgeny R, Khatsenovich A, Queffelec A, Paine C, Gunchinsuren B, et al. Symbolic innovation at the onset of the Upper Paleolithic in Eurasia shown by the personal ornaments from Tolbor-21 (Mongolia). Sci Rep. 2023;13. doi:10.1038/s41598-023-36140-1.

9. d’Errico F, David S, Coqueugniot H, Meister C, Dutkiewicz E, Pigeaud R, et al. A 36,200-year-old carving from Grotte des Gorge, Amange, Jura, France. Sci Rep. 2023;13 : 12895. doi: 10.1038/s41598-023-39897-7.

10. Ma S, Doyon L, Zhang Y, Li Z. Disentangling carcass processing activities and the state of worked hide from use-wear patterns on expedient bone tools: A preliminary experiment. J Archaeol Sci Rep. 2023;49: 104027. doi:10.1016/j.jasrep.2023.104027.

11. Schulz E, Piotrowski V, Clauss M, Mau M, Merceron G, Kaiser TM. Dietary abrasiveness is associated with variability of microwear and dental surface texture in rabbits. PLOS ONE. 2013;8: e56167. doi:10.1371/journal.pone.0056167.

12. Caux S, Galland A, Queffelec A, Bordes J-G. Aspects and characterization of chert alteration in an archaeological context: A qualitative to quantitative pilot study. J Archaeol Sci Rep. 2018;20: 210–219. doi:10.1016/j.jasrep.2018.04.027.

13. Galland A, Queffelec A, Caux S, Bordes J-G. Quantifying lithic surface alterations using confocal microscopy and its relevance for exploring the Châtelperronian at La Roche-à-Pierrot (Saint-Césaire, France). J Archaeol Sci Rep. 2019;104: 45–55. doi:10.1016/j.jas.2019.01.009.

S1 Fig. Experimental blocks before and after experimentation.

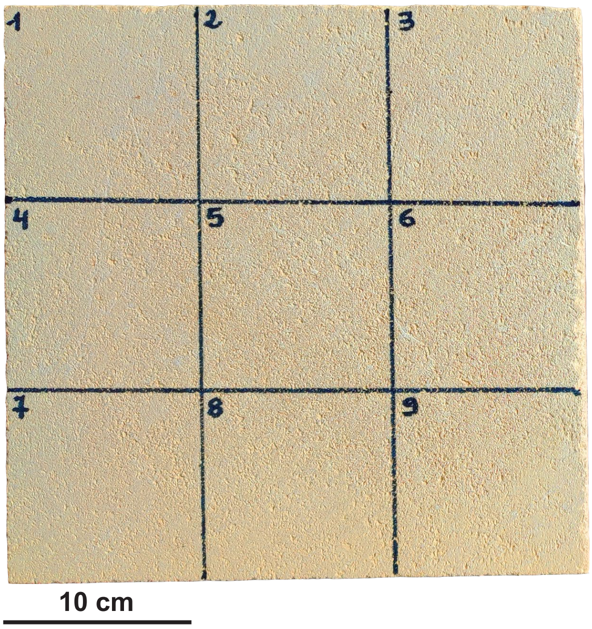

Legend
CS: Control Surface;
PIPB: Pecking in Indirect Percussion with a Blade;
PIPP: Pecking in Indirect Percussion with a Pick;
PDPP: Pecking in Direct Percussion with a Pick;
PDPC: Pecking in Direct Percussion with a Cobble;
PSc: Pecking in direct percussion with a broken blade and Scraping with an endScraper;
ScS: Scraping with an endScraper;
ScB: Scraping with a Blade;
PScPo: Pecking in direct percussion with a broken blade, Scraping with an endscraper and Polishing with Skin;
PoHS: Polishing with Humid Sand;
PoS: Polishing with Skin;
PoC: Polishing with a Cobble;
Sets: Engravings (see Table 5 for more details).

A. Block divided into nine 10 × 10 cm squares

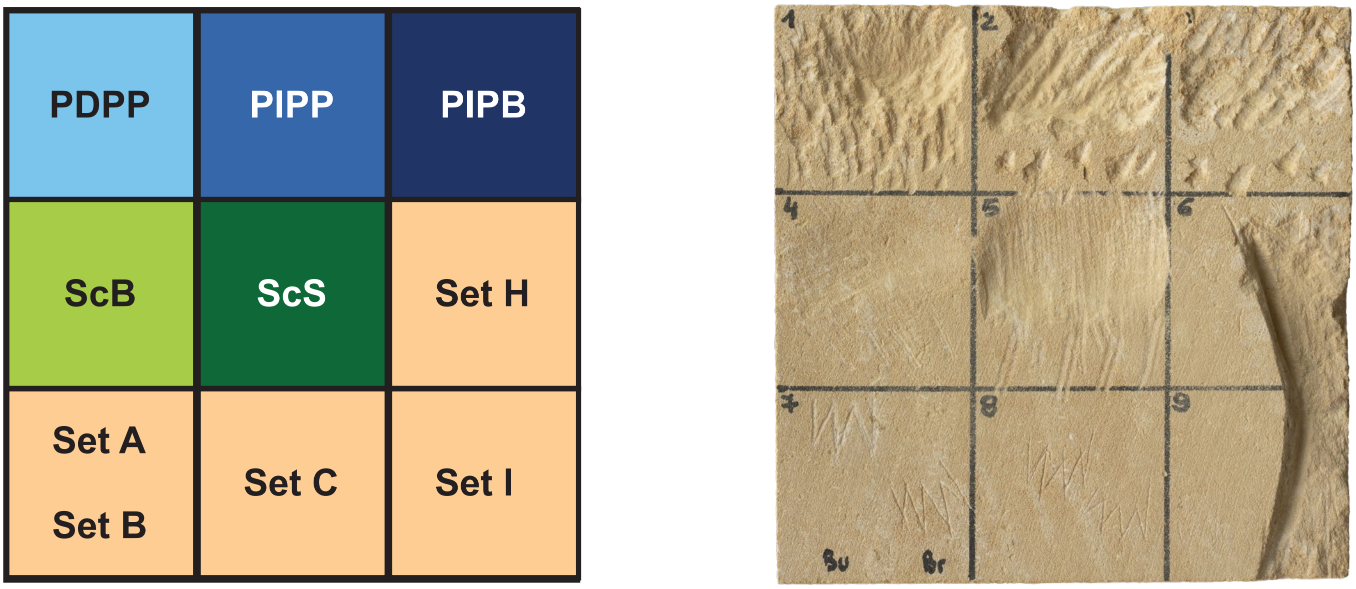

B. Block after completion of experimental Phase 1

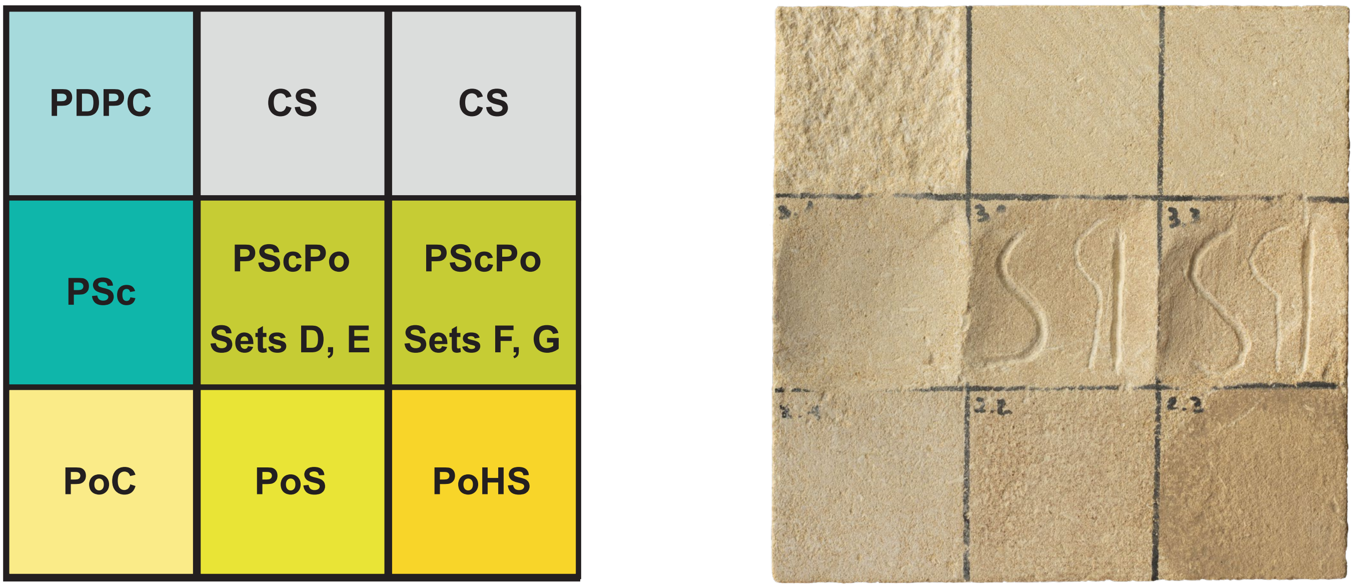

C. Block after completion of experimental Phase 2

S2 Fig. Experimental modality square digitally subdivided into nine non-overlapping 10 × 10 mm sub-areas.

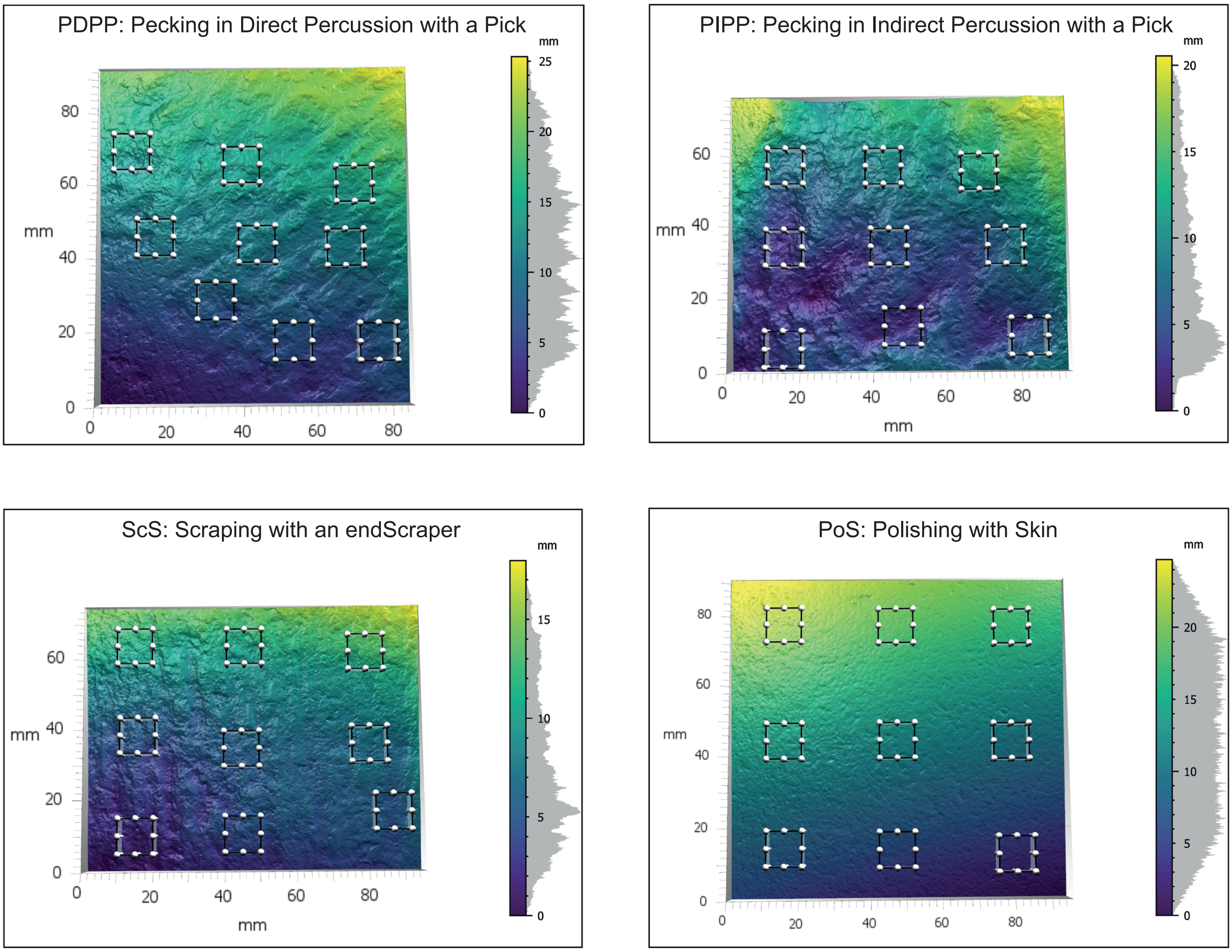

The nine areas were manually adjusted to capture as much variation as possible, without overlapping.

S3 Fig. Experimental blocks of phases 1 and 2 according to levels of expertise.

Experimental phase 1

Novice 1

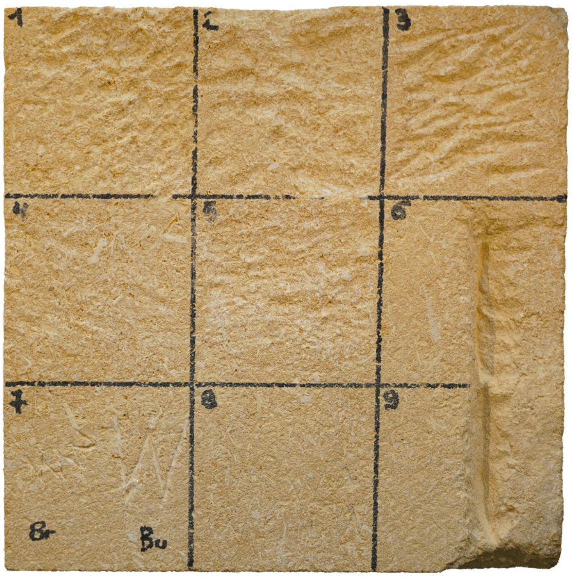

Novice 2

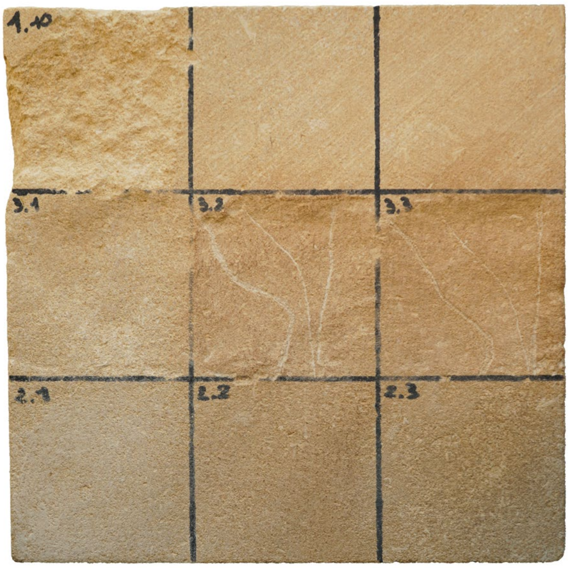

Intermediate 1

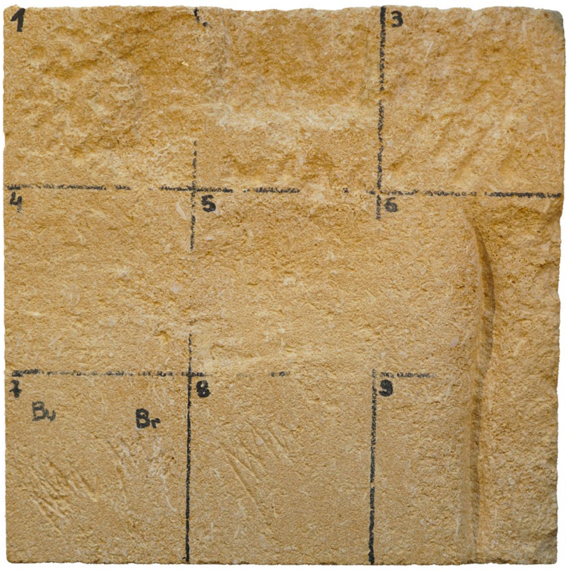

Intermediate 2

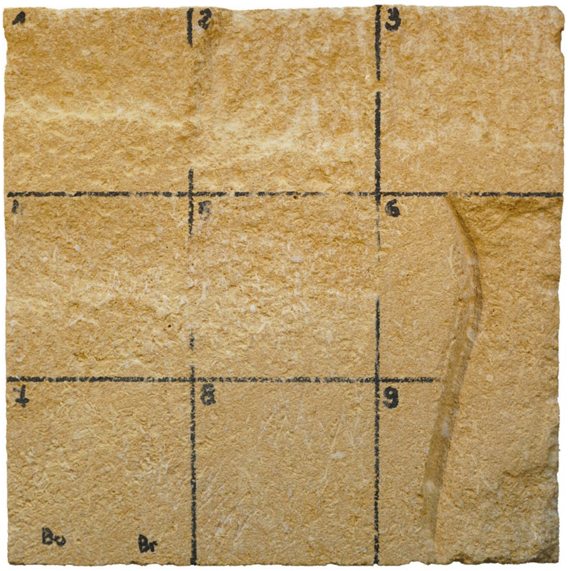

Expert 1

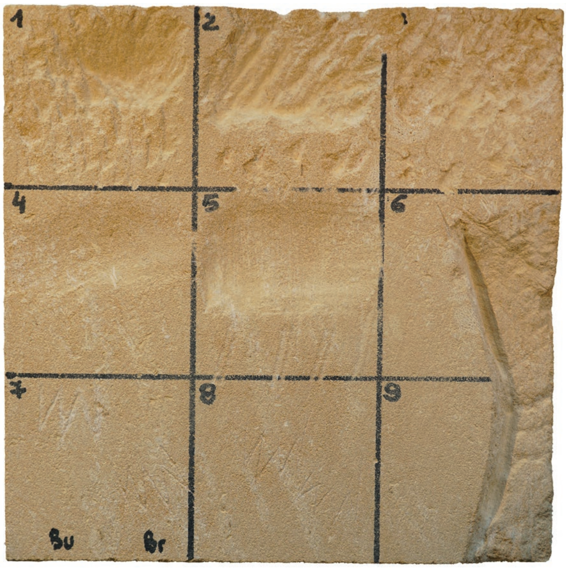

10 cm

Expert 1-RTI (Reflectance Transformation Imaging)

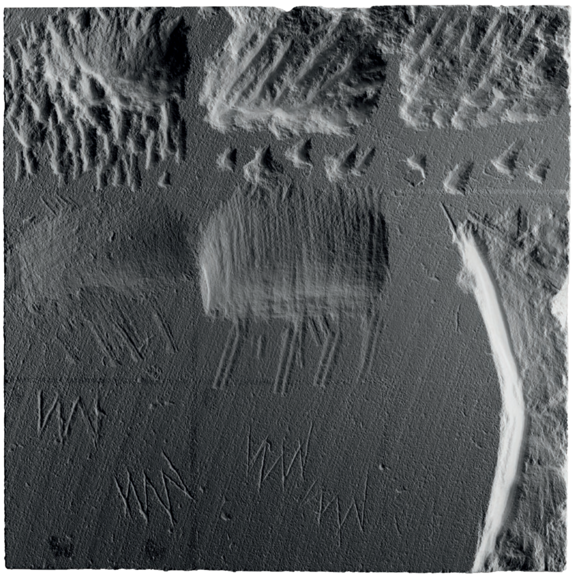

Experimental phase 2

Novice 2

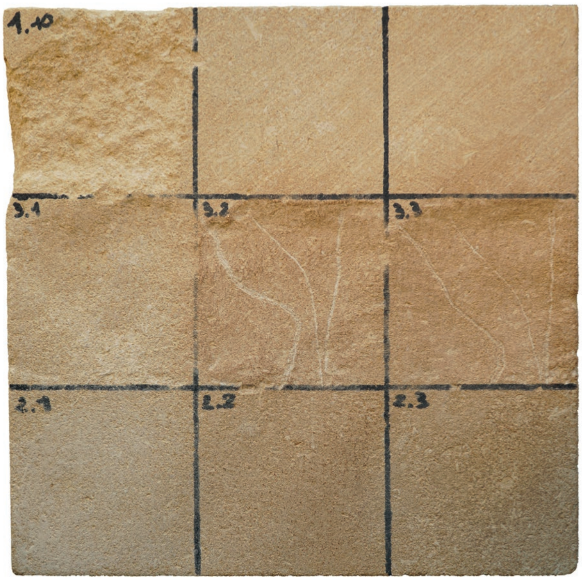

Novice 3

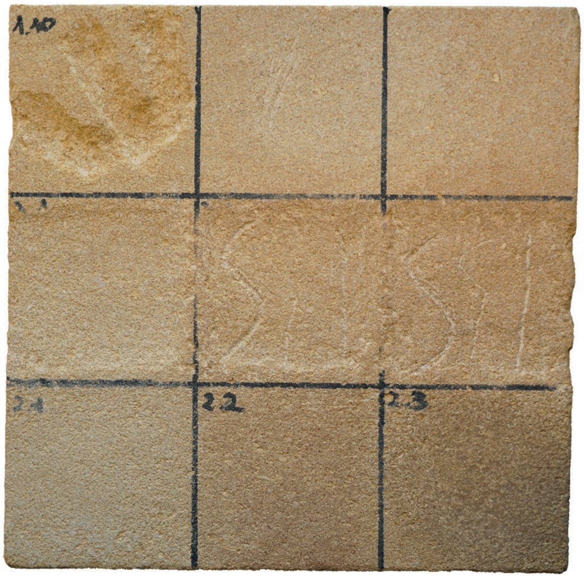

Novice 4

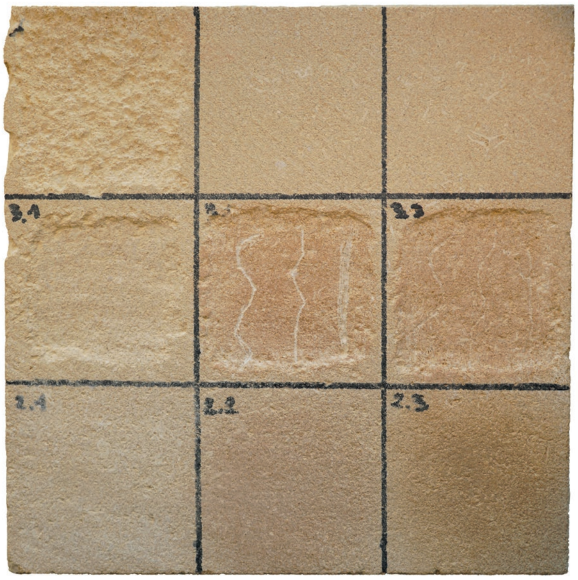

Intermediate 2

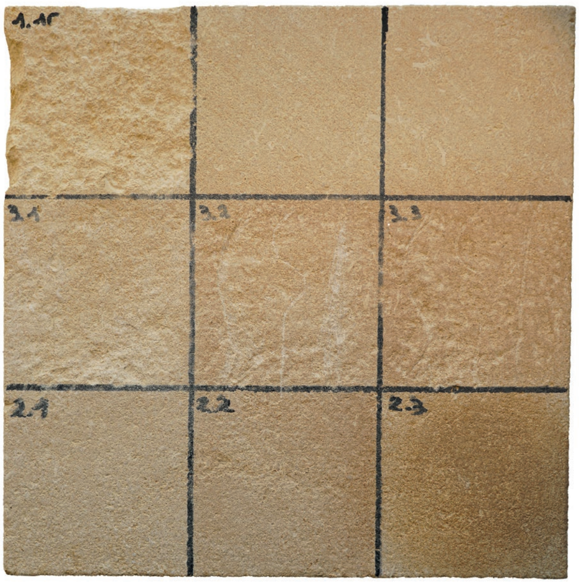

Expert 1

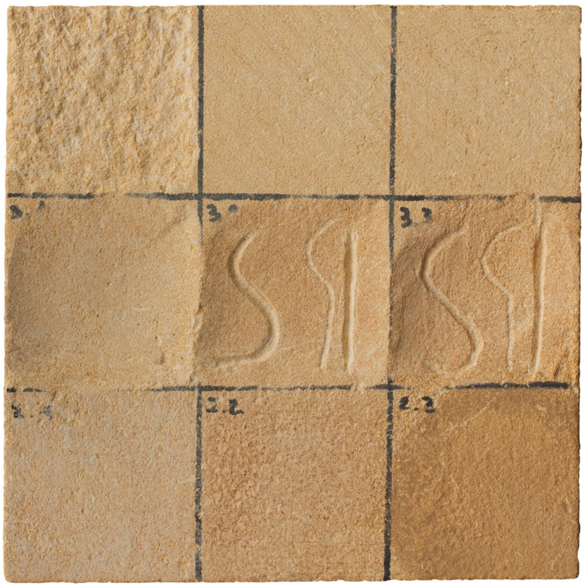

Expert 1-RTI (Reflectance Transformation Imaging)

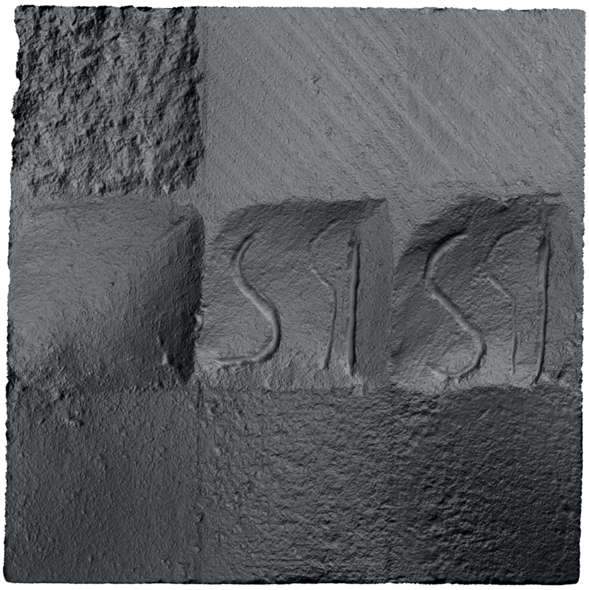

10 cm

S4 Fig. Boxplots of non-significant and highly correlated linear parameters for the technique analysis.

Non-significant parameters

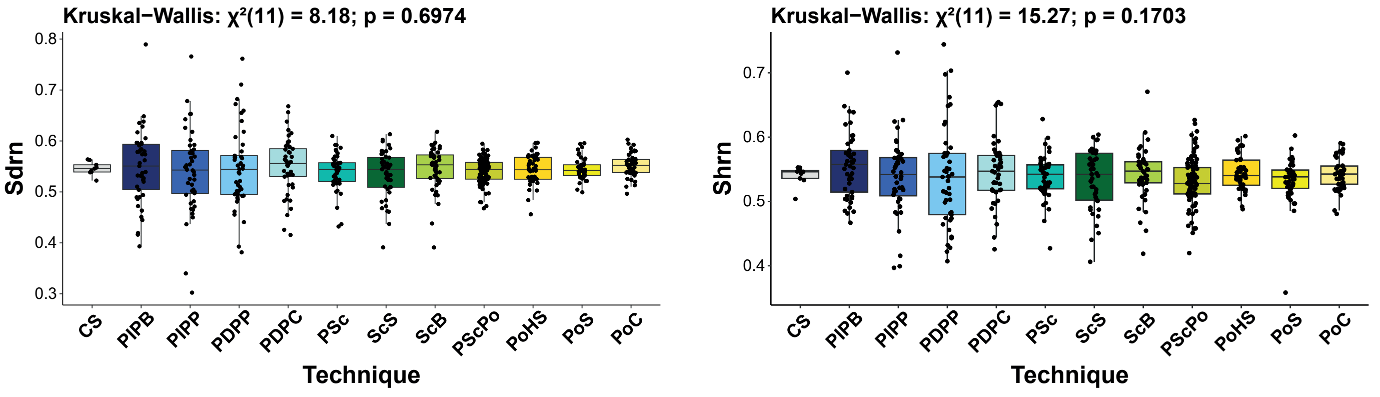

Highly correlated parameters

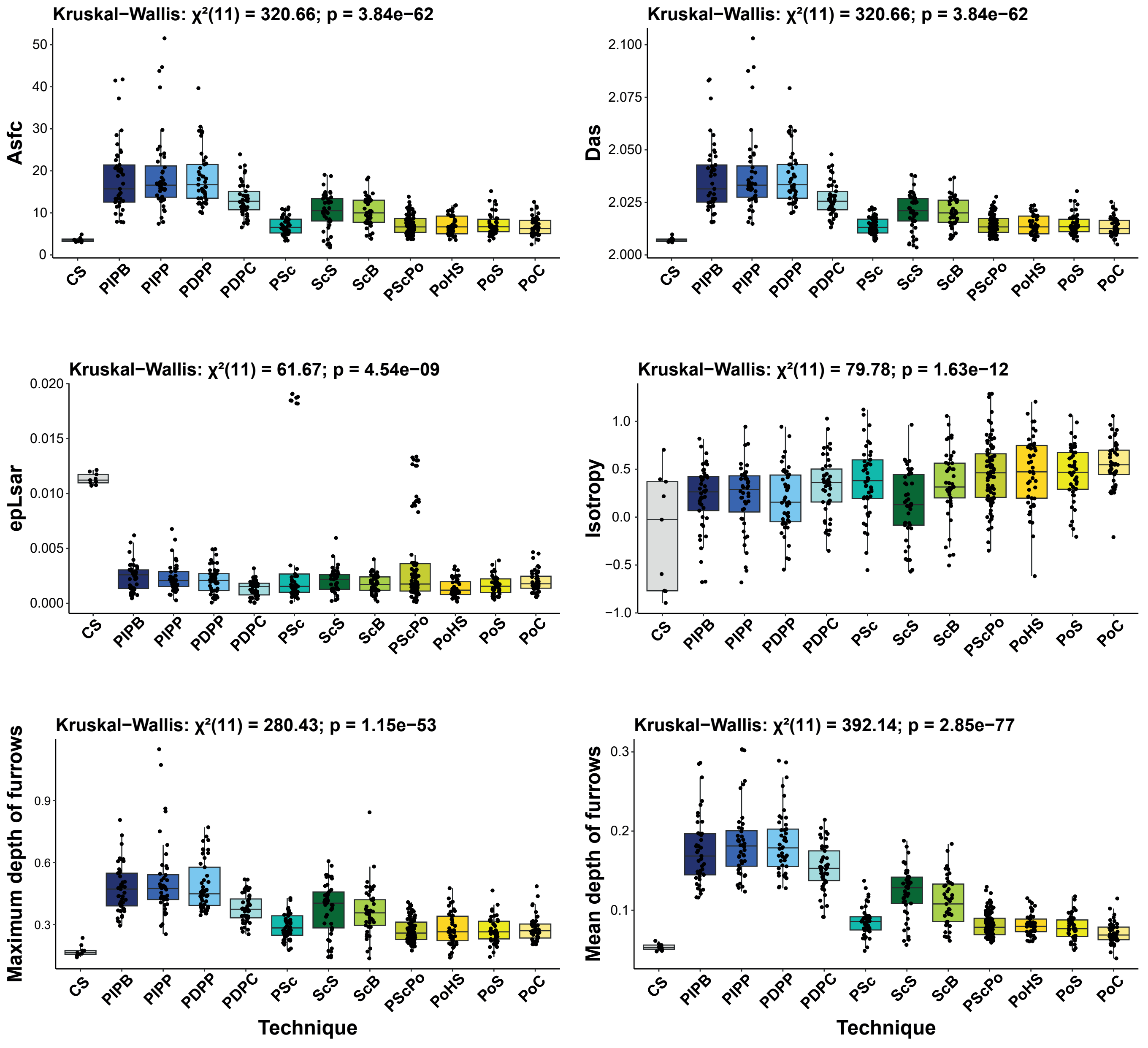

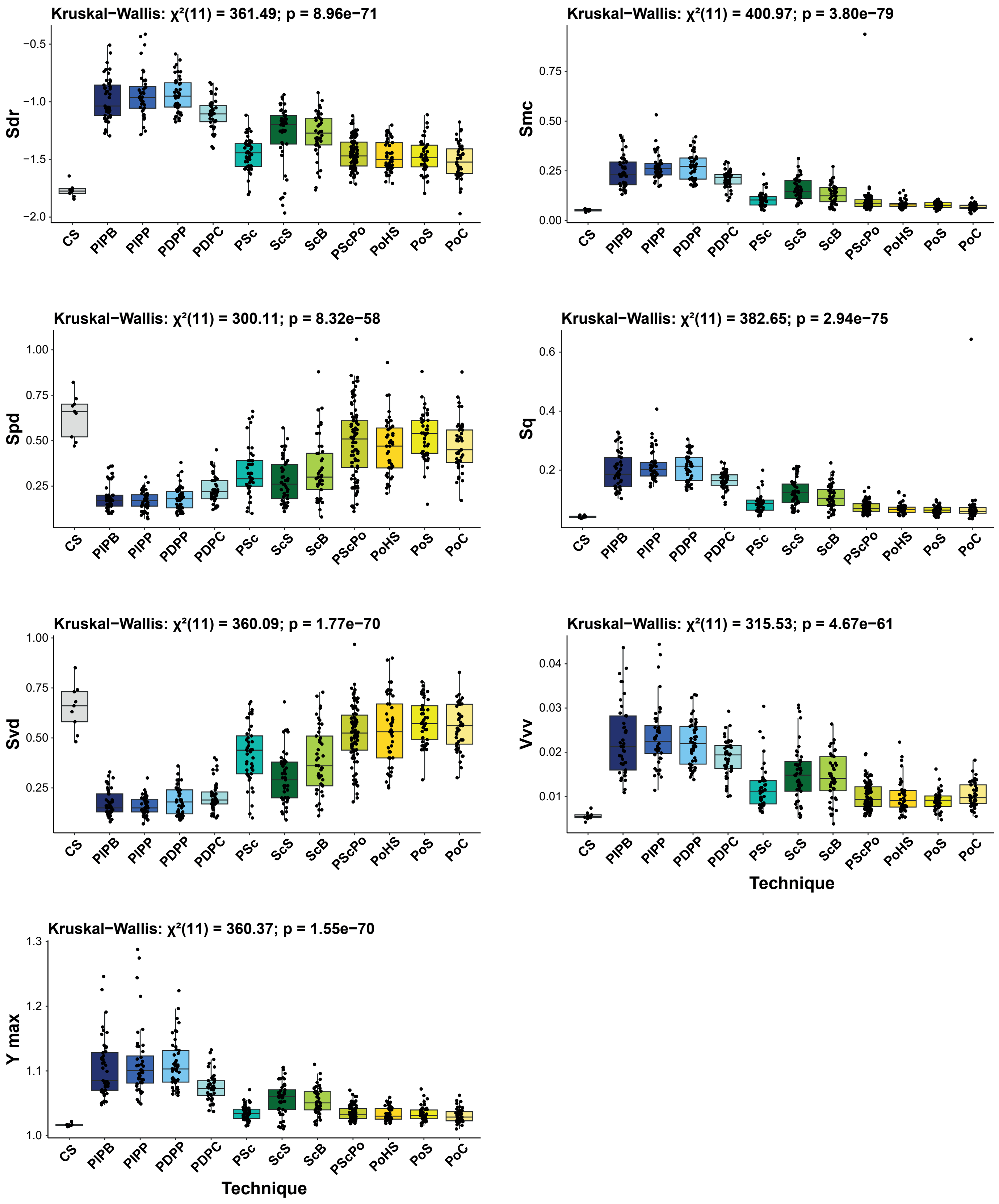
Kruskal−Wallis: χ²(11) = 361.49; p = 8.96e−71
Sdr
Kruskal−Wallis: χ²(11) = 400.97; p = 3.80e−79
Smc
Kruskal−Wallis: χ²(11) = 300.11; p = 8.32e−58
Spd
Kruskal−Wallis: χ²(11) = 382.65; p = 2.94e−75
Sq
Kruskal−Wallis: χ²(11) = 360.09; p = 1.77e−70
Svd
Kruskal−Wallis: χ²(11) = 315.53; p = 4.67e−61
Vvv
Kruskal−Wallis: χ²(11) = 360.37; p = 1.55e−70
Y max
CS PIPB PIPP PDPP PDPC PSc ScS ScB PScPo PoHS PoS PoC
Technique

S5 Fig. Rose diagrams of non-significant circular parameters in the technique analysis.

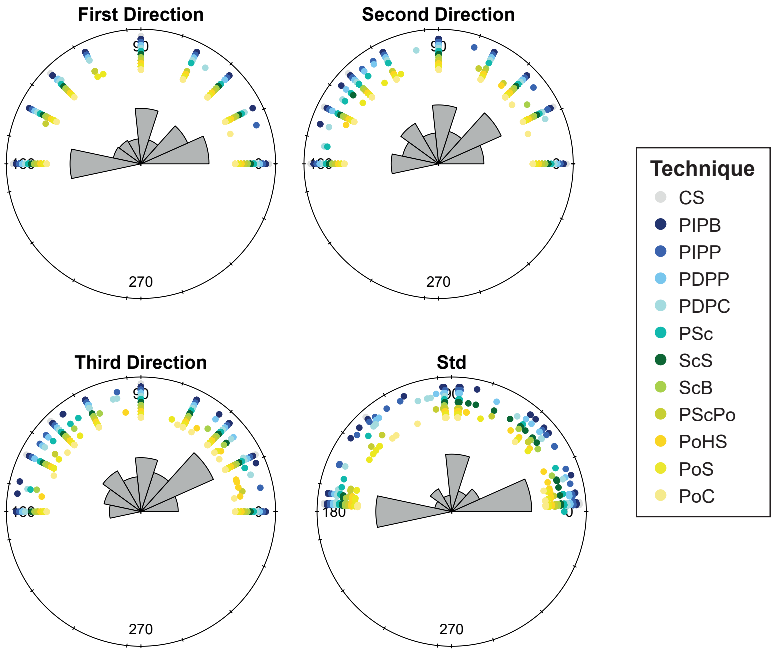

S6 Fig. CVA plots for the technique analysis, with overall data and by skill level.

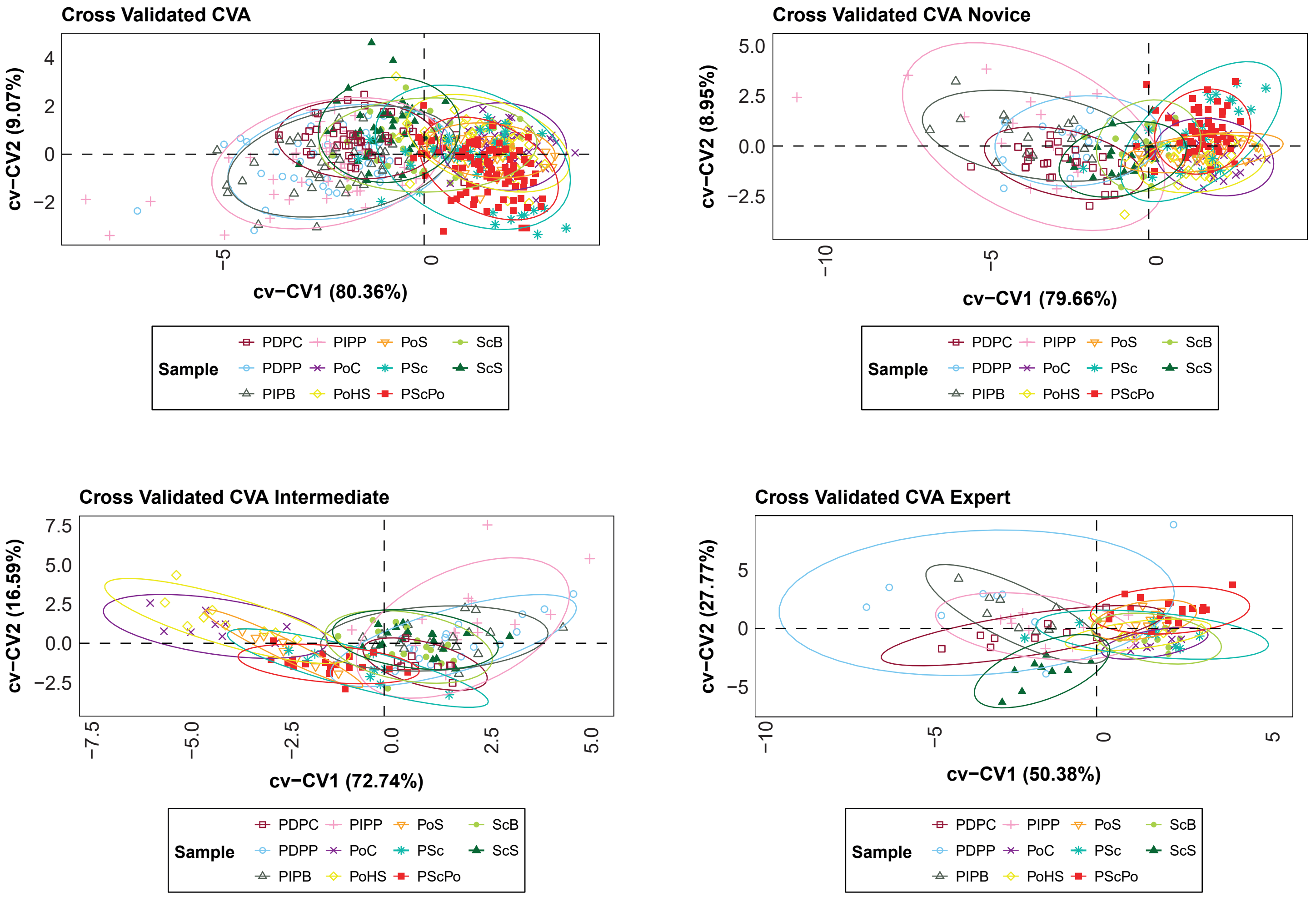

S7 Fig. Representative engraving profiles for each set.

Set	Engraving type	Technique description	Engraving stroke count	Engraving tool (active part)
Set A	Superficial	Engraving	Multiple strokes	Burin (point)
Set B				Flake (unretouched edge)
Set C				Blade (unretouched edge)
Set D		Pecking + Scraping + Polishing + Engraving	Single stroke	Blade (unretouched edge)
Set E			Multiple strokes	
Set F		Pecking + Scraping + Engraving + Polishing	Single stroke	
Set G			Multiple strokes	
Set H	Deep	Pecking + Engraving	Multiple strokes	Burin (point)
Set I				Pick (point)

Set A

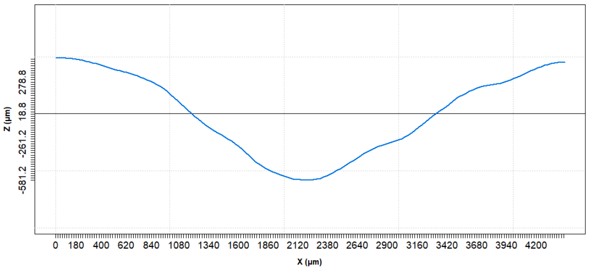

Set B

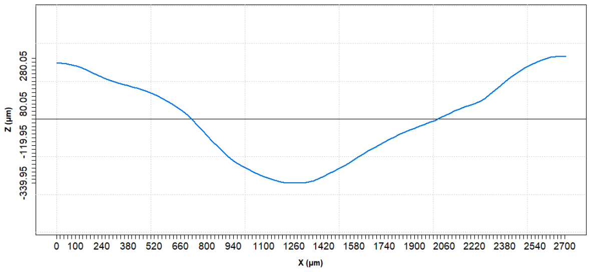

Set C

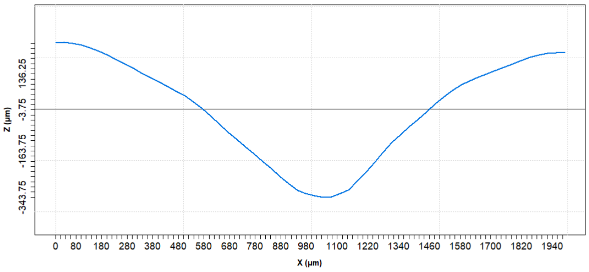

Set D

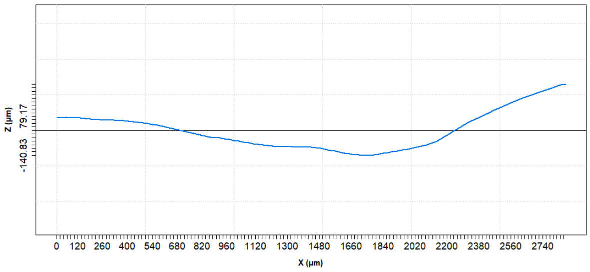

Set E

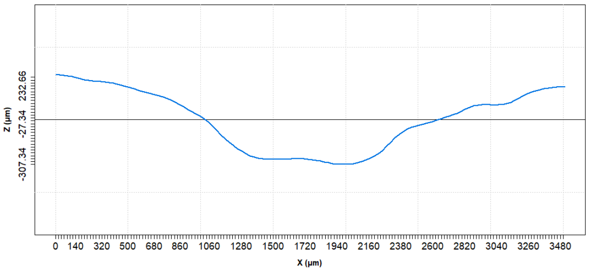

Set F

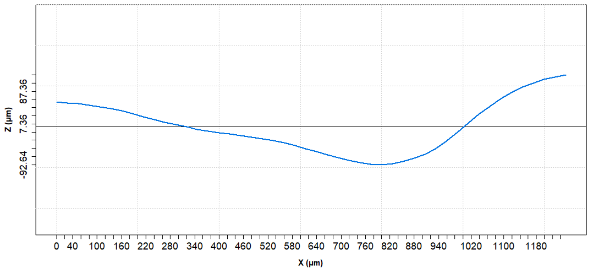

Set G

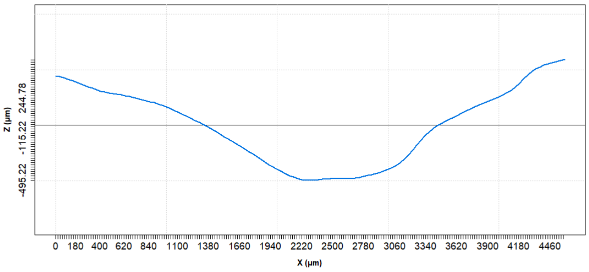

Set H

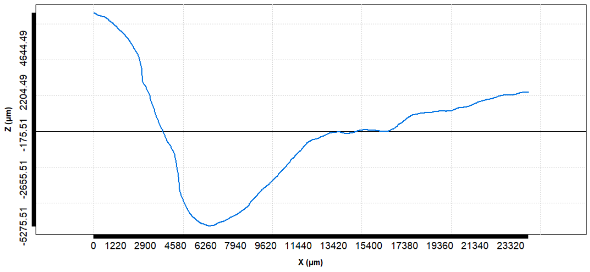

Set I

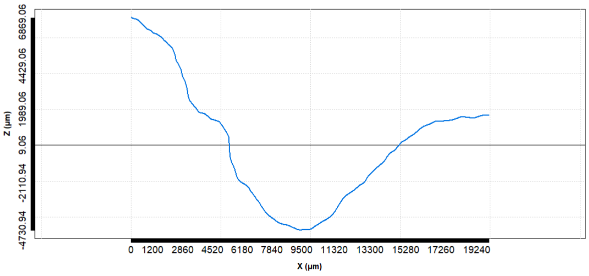

S8 Fig. Distribution of opening angles by engraved set.

Superficial engravings

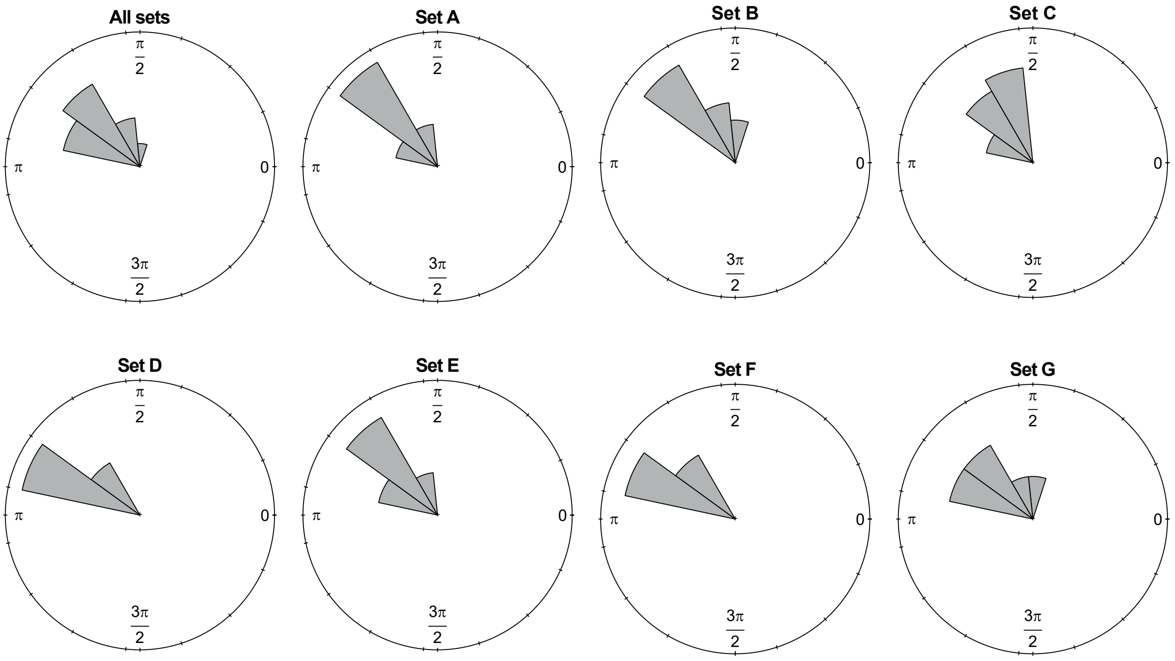

Deep engravings

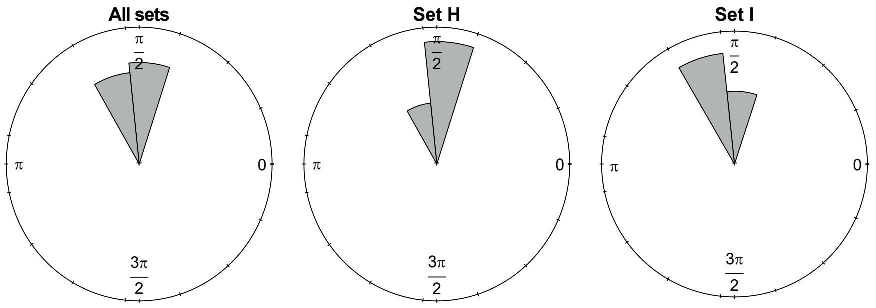

S9 Fig. Distribution of Depth, WIS, and A in the deep engraving analysis.

Set H: Pecking + Engravings produced with a burin using multiple stroke
Set I: Pecking + Engravings produced with a pick using multiple strokes

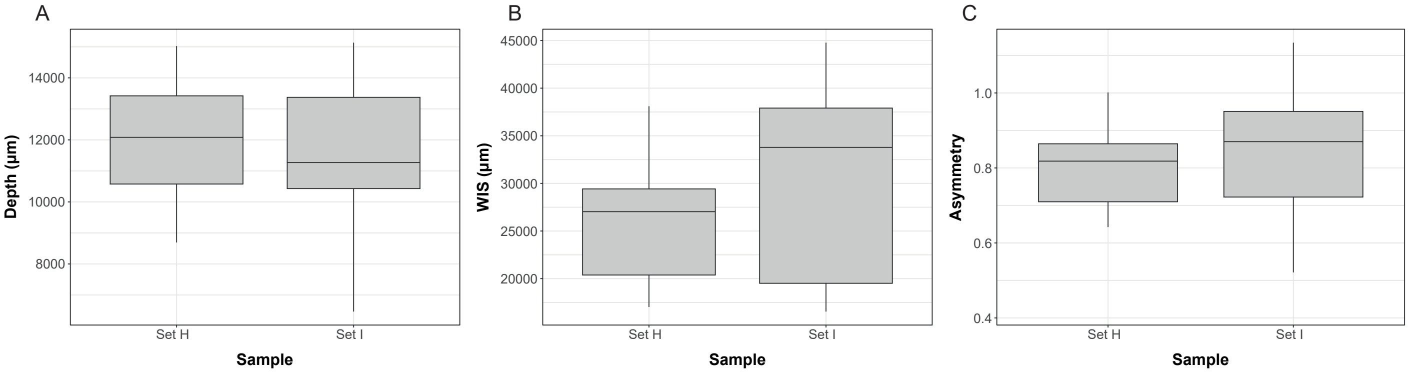

S10 Fig. Boxplots of non-significant and highly correlated linear parameters in the expertise analysis.

Non-significant parameters

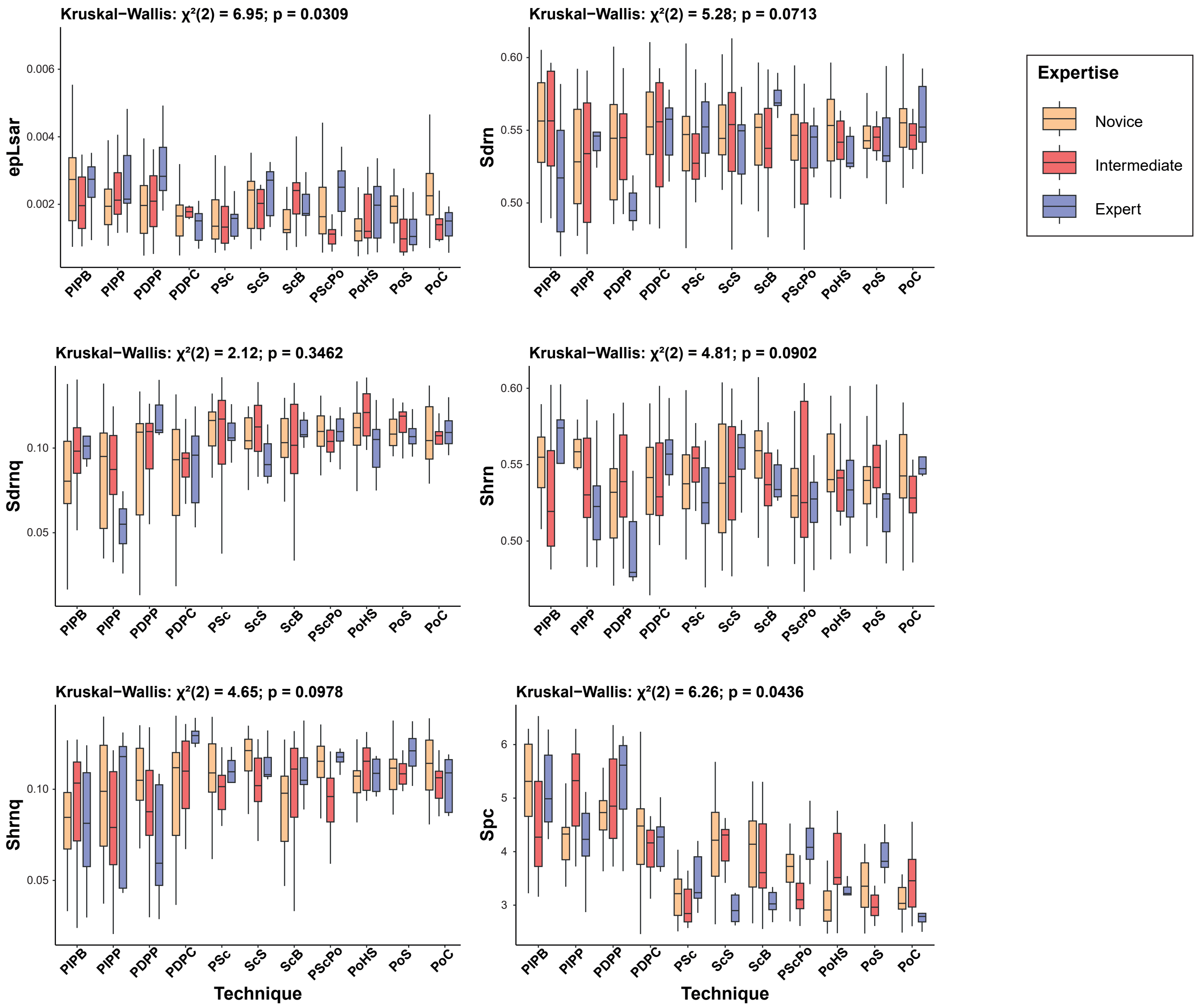

Highly-correlated parameters

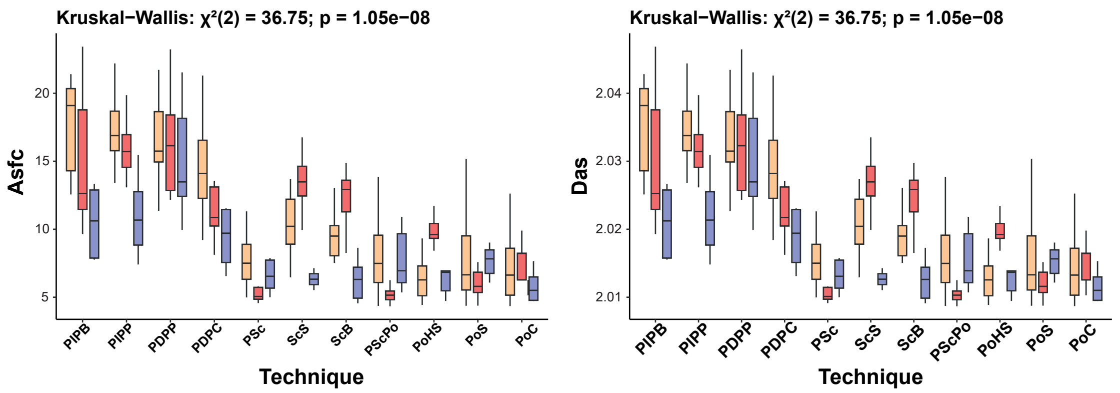

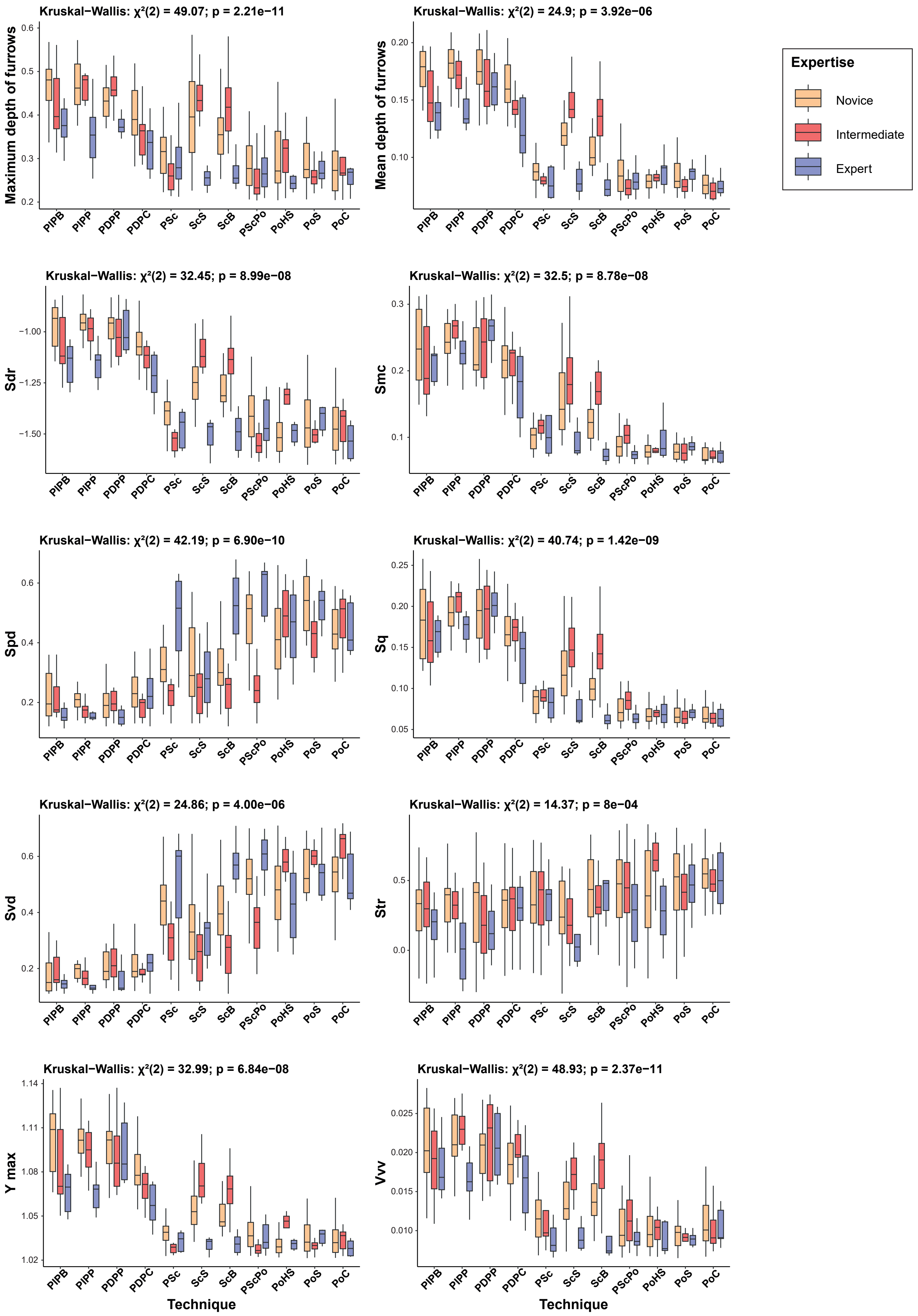
Kruskal−Wallis: χ²(2) = 49.07; p = 2.21e−11
Maximum depth of furrows
Kruskal−Wallis: χ²(2) = 24.9; p = 3.92e−06
Mean depth of furrows
Expertise
Novice
Intermediate
Expert
Kruskal−Wallis: χ²(2) = 32.45; p = 8.99e−08
Sdr
Kruskal−Wallis: χ²(2) = 32.5; p = 8.78e−08
Smc
Kruskal−Wallis: χ²(2) = 42.19; p = 6.90e−10
Spd
Kruskal−Wallis: χ²(2) = 40.74; p = 1.42e−09
Sq
Kruskal−Wallis: χ²(2) = 24.86; p = 4.00e−06
Svd
Kruskal−Wallis: χ²(2) = 14.37; p = 8e−04
Str
Kruskal−Wallis: χ²(2) = 32.99; p = 6.84e−08
Y max
Kruskal−Wallis: χ²(2) = 48.93; p = 2.37e−11
Vvv
PIPB PIPP PDPP PDPC PSc ScS ScB PScPo PoHS PoS PoC
Technique

S11 Fig. Rose diagram of non-significant circular parameters in the expertise analysis.

Second Direction

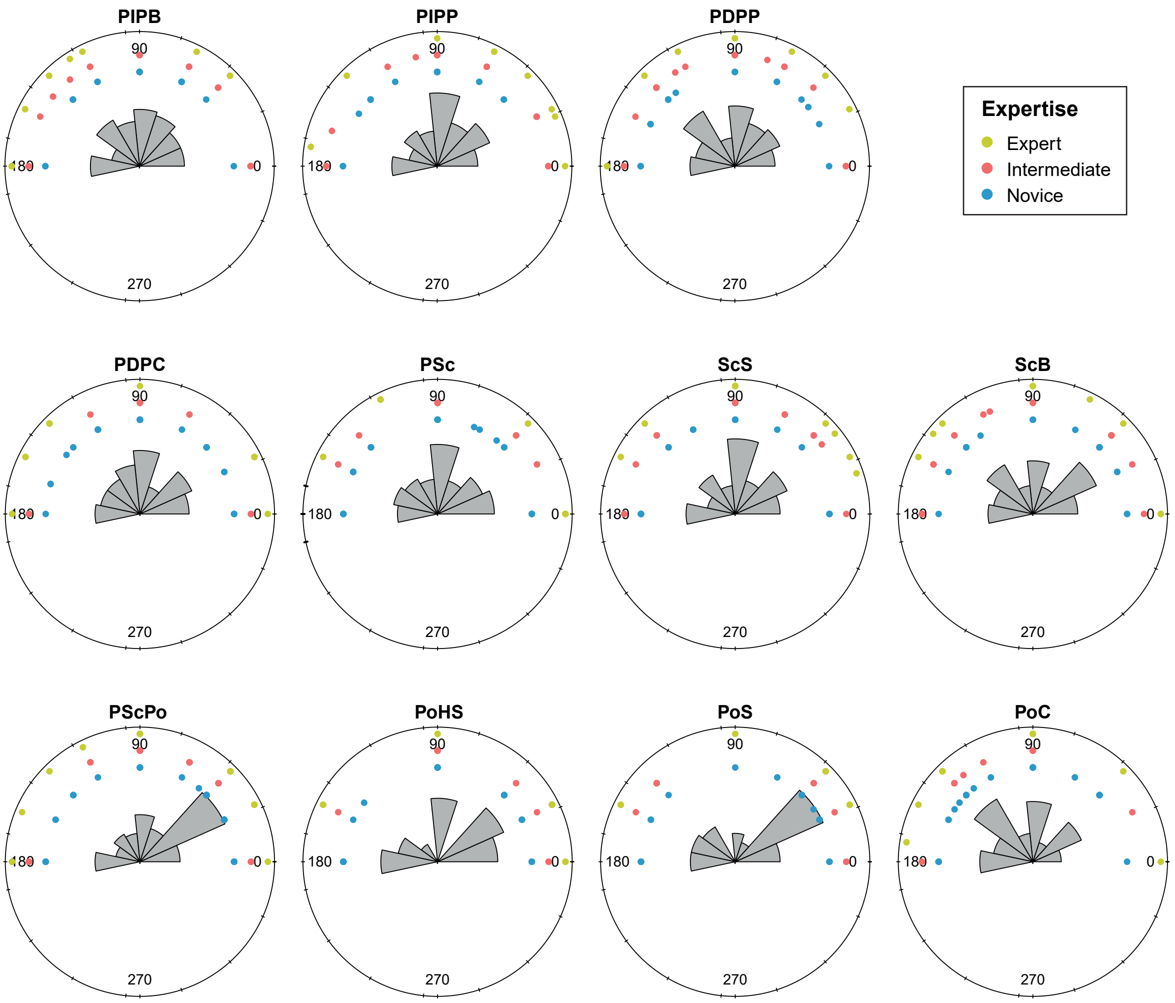

Third Direction

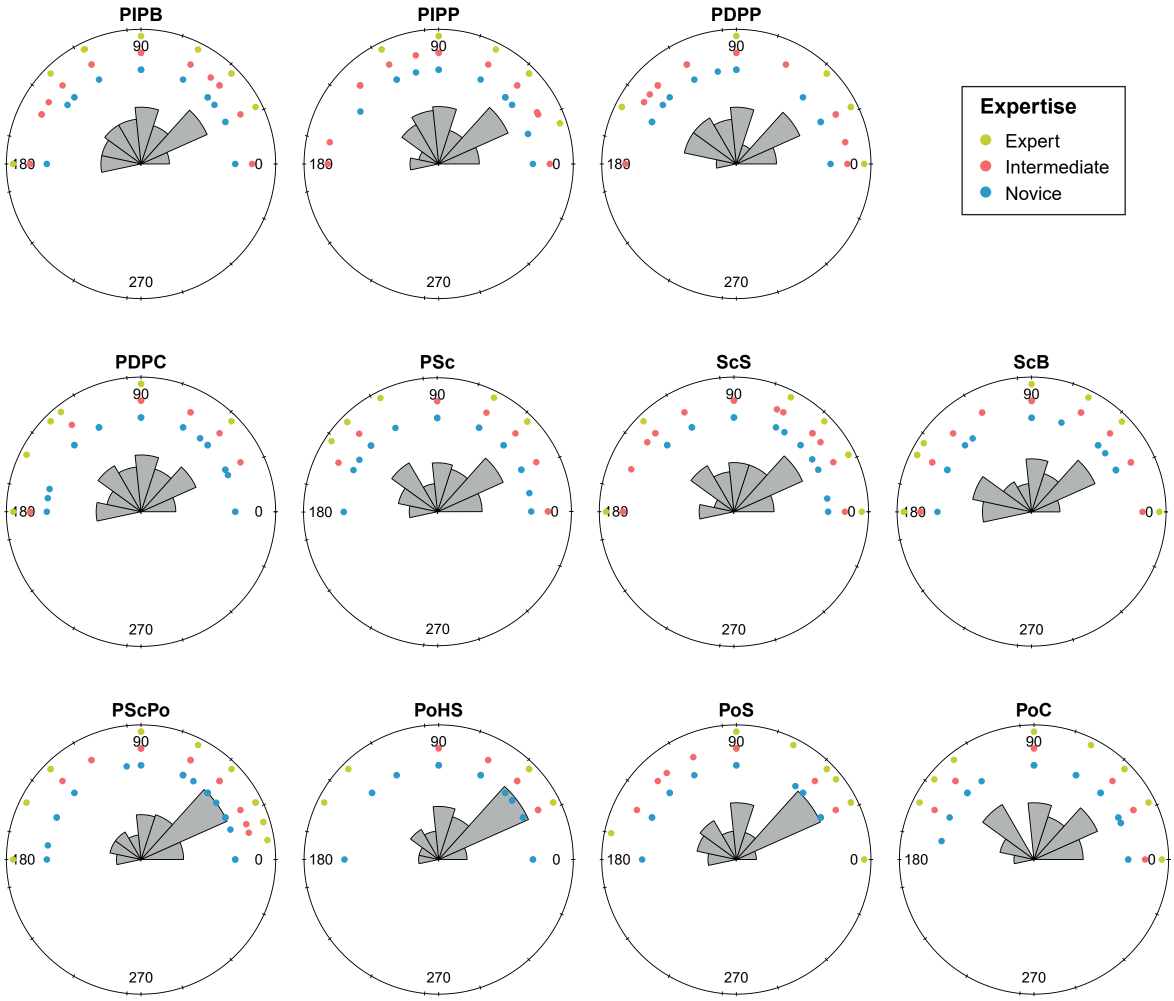

Std

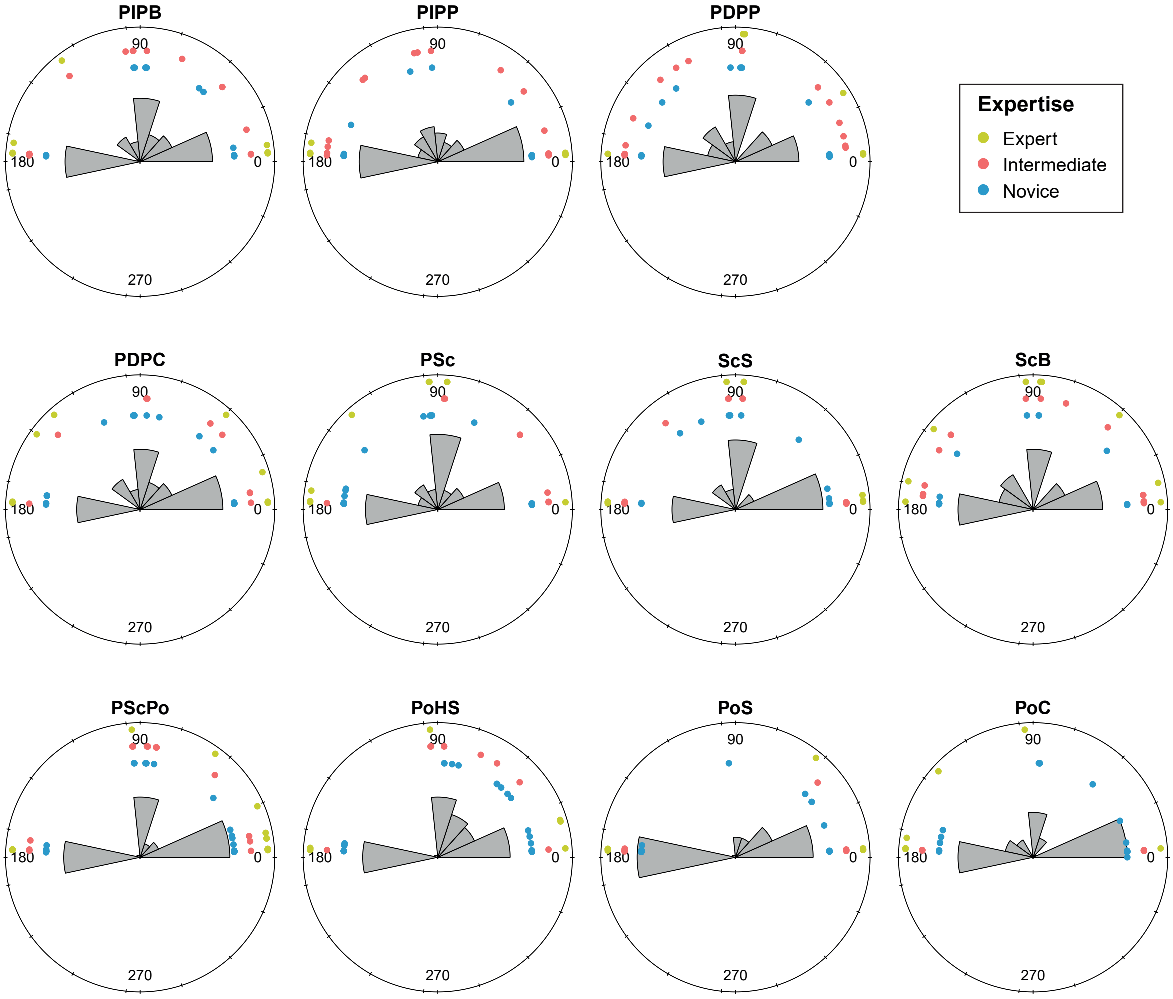
PIPB
PIPP
PDPP
PDPC
PSc
ScS
ScB
PScPo
PoHS
PoS
PoC
90
180
0
270
Expertise
Expert
Intermediate
Novice

S12 Fig. CVA plots by technique for the expertise analysis.

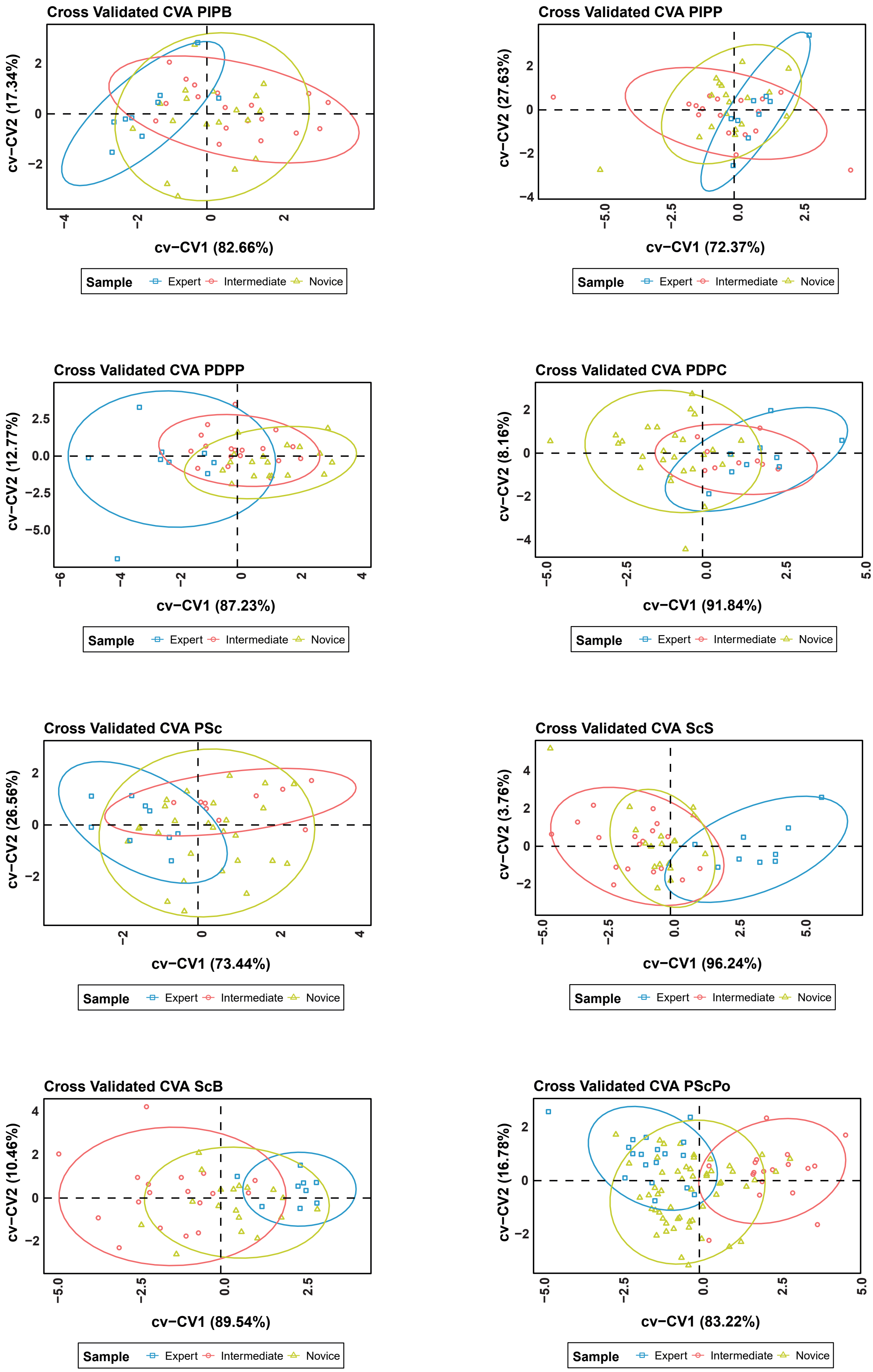

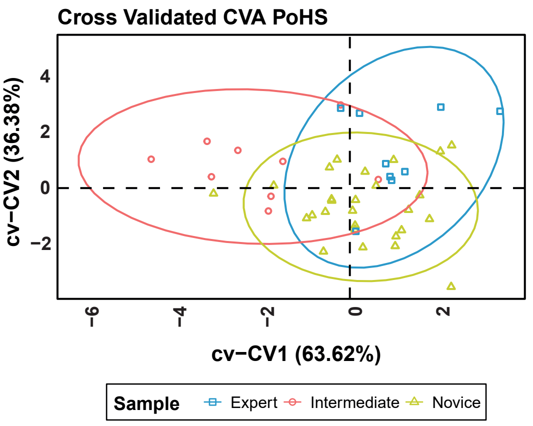
Cross Validated CVA PoHS
cv−CV2 (36.38%)
cv−CV1 (63.62%)
Sample
Expert
Intermediate
Novice

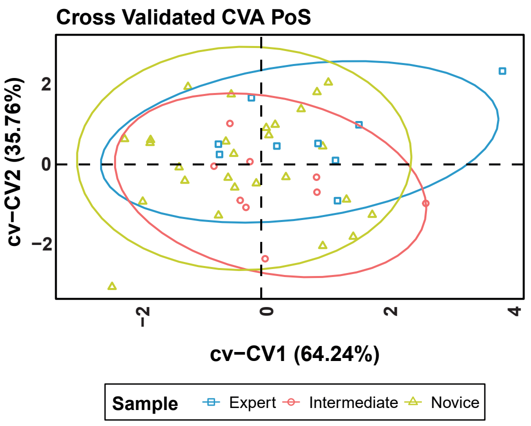
Cross Validated CVA PoS
cv−CV2 (35.76%)
cv−CV1 (64.24%)
Sample
Expert
Intermediate
Novice

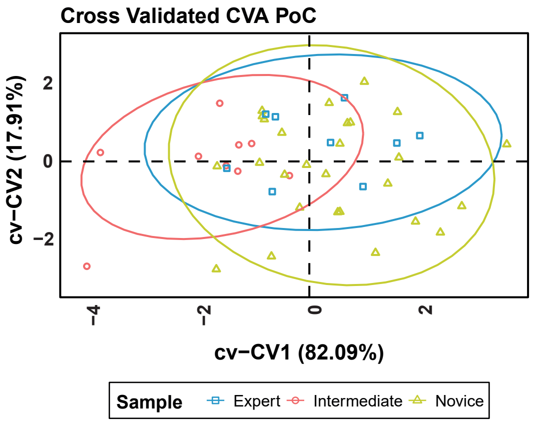
Cross Validated CVA PoC
cv−CV2 (17.91%)
cv−CV1 (82.09%)
Sample
Expert
Intermediate
Novice
