# Supplementary material for: Carved in stone: Experimental criteria for identifying Paleolithic bas-relief production techniques and sculptors’ expertise
Source: PLoS One. 2026 Apr 1;21(4):e0346099. doi: 10.1371/journal.pone.0346099 (PMC13042861; doi:10.1371/journal.pone.0346099)
Supplement: S2 File — (PDF) [file pone.0346099.s002.pdf]

Supplementary File 2: Tables

Contents

S1 Table. Normality and group comparison tests for technique-based analysis. 2

S2 Table. Angular data statistics for technique analysis. 2

S3 Table. Correlation matrix for technique-related analysis. 3

S4 Table. Descriptive statistics for linear roughness variables used in the technique analysis. 5

S5 Table. Summary of LDA statistics by class for the technique analysis: overall data and by skill level. 11

S6 Table. LDA confusion matrices for the technique analysis: overall data and by skill level. 13

S7 Table. Normality and group comparison tests for the analysis of engravings. 14

S8 Table. Summary of linear statistical parameters (Depth, WIS, A) in the engraving analysis. 14

S9 Table. Summary of circular statistics and orientation parameters for the engraved sets. 16

S10 Table. MANOVA based on PC1 and PC2 from the metric PCA to assess intergroup variation among superficial engravings. 17

S11 Table. MANOVA based on PC1 and PC2 from the EFA PCA to assess intergroup variation among superficial engravings. 17

S12 Table. Normality and group comparison tests for the analysis of expertise. 18

S13 Table. Statistical results of angular data for the analysis of expertise. 18

S14 Table. Correlation matrix values for expertise analysis. 19

S15 Table. Descriptive statistics for linear roughness variables used in the expertise analysis. 21

S16 Table. Descriptive statistics for circular roughness variables used in the expertise analysis. 29

S17 Table. Summary of LDA statistics by class within technique for the expertise analysis. 31

S18 Table. LDA confusion matrices by technique for the expertise analysis. 33

S1 Table. Normality and group comparison tests for technique-based analysis.

**Variable**	**Test**	**Shapiro *p***	**Shapiro *W***	**Df**	**Test statistic**	***p*-value**
Sq	kruskal	1.47E-21	8.59E-01	10	368.93	3.81E-73
Smc	kruskal	5.04E-23	8.38E-01	10	388.16	3.11E-77
Sal	kruskal	1.84E-06	9.81E-01	10	220.21	9.65E-42
Str norm	kruskal	9.91E-04	9.90E-01	10	74.63	5.62E-12
Sdr norm	kruskal	2.03E-05	9.85E-01	10	348.04	1.04E-68
Vvv	kruskal	1.59E-16	9.18E-01	10	300.67	1.12E-58
Spd	kruskal	1.95E-14	9.36E-01	10	288.73	3.74E-56
Spc	kruskal	9.78E-19	8.95E-01	10	258.46	9.03E-50
Svd	kruskal	5.09E-13	9.47E-01	10	351.48	1.93E-69
Shrn	kruskal	3.21E-10	9.64E-01	10	15.02	1.31E-01
Shrnq	kruskal	4.77E-20	8.79E-01	10	53.80	5.28E-08
Sdrn	kruskal	8.38E-15	9.33E-01	10	8.10	6.19E-01
Sdrnq	kruskal	3.15E-20	8.77E-01	10	50.39	2.26E-07
Y max	kruskal	7.01E-24	8.24E-01	10	346.86	1.85E-68
Asfc	kruskal	8.38E-24	8.25E-01	10	306.36	7.03E-60
Das	kruskal	8.38E-24	8.25E-01	10	306.36	7.03E-60
Smfc	kruskal	9.54E-35	5.56E-01	10	285.64	1.68E-55
HAsfc	kruskal	9.36E-32	6.50E-01	10	174.23	3.69E-32
MedianAsfc	kruskal	1.00E-24	8.09E-01	10	271.71	1.46E-52
epLsar	kruskal	2.91E-35	5.38E-01	10	39.71	1.91E-05
NewEplsar	kruskal	3.33E-31	6.65E-01	10	105.79	3.76E-18
Isotropy norm	kruskal	9.91E-04	9.90E-01	10	74.63	5.62E-12
Maximum depth of furrows	kruskal	6.66E-18	9.04E-01	10	265.34	3.21E-51
Mean depth of furrows	kruskal	3.44E-18	9.01E-01	10	378.66	3.26E-75
Mean density of furrows	ANOVA	2.04E-01	9.96E-01	10	52.97	2.94E-73

S2 Table. Angular data statistics for technique analysis. *Wg and Pg are the test statistics for the corresponding p-values (p).*

**Variable**	**Test**	**Df**	***Wg***	***p*-value**	**Test 2**	***Pg***	***p*-value**
Std	Mardia-Watson-Wheeler	2	3.36	0.19	Fisher's Non-Parametric	1.11	0.29
First Direction	Mardia-Watson-Wheeler	2	5.10	0.08	Fisher's Non-Parametric	1.36	0.24
Second Direction	Mardia-Watson-Wheeler	2	4.60	0.10	Fisher's Non-Parametric	2.18	0.14
Third Direction	Mardia-Watson-Wheeler	2	1.93	0.38	Fisher's Non-Parametric	1.11	0.29

S3 Table. Correlation matrix for technique-related analysis.

	Sq	Smc	Sal	Str	Sdr	Vvv	Spd	Spc	Svd	Shrnq	Sdrnq	Y max
**Sq**	1.00	0.86	0.63	-0.25	0.87	0.87	-0.69	0.65	-0.76	-0.42	-0.34	0.87
**Smc**	0.86	1.00	0.63	-0.28	0.84	0.83	-0.64	0.65	-0.74	-0.40	-0.33	0.84
**Sal**	0.63	0.63	1.00	-0.13	0.51	0.64	-0.77	0.32	-0.79	-0.31	-0.30	0.45
**Str**	-0.25	-0.28	-0.13	1.00	-0.21	-0.21	0.28	-0.16	0.31	0.18	0.10	-0.20
**Sdr**	0.87	0.84	0.51	-0.21	1.00	0.89	-0.61	0.75	-0.68	-0.34	-0.28	0.94
**Vvv**	0.87	0.83	0.64	-0.21	0.89	1.00	-0.72	0.61	-0.77	-0.41	-0.33	0.88
**Spd**	-0.69	-0.64	-0.77	0.28	-0.61	-0.72	1.00	-0.32	0.91	0.39	0.31	-0.56
**Spc**	0.65	0.65	0.32	-0.16	0.75	0.61	-0.32	1.00	-0.44	-0.27	-0.23	0.76
**Svd**	-0.76	-0.74	-0.79	0.31	-0.68	-0.77	0.91	-0.44	1.00	0.38	0.36	-0.64
**Shrnq**	-0.42	-0.40	-0.31	0.18	-0.34	-0.41	0.39	-0.27	0.38	1.00	0.27	-0.37
**Sdrnq**	-0.34	-0.33	-0.30	0.10	-0.28	-0.33	0.31	-0.23	0.36	0.27	1.00	-0.29
**Y max**	0.87	0.84	0.45	-0.20	0.94	0.88	-0.56	0.76	-0.64	-0.37	-0.29	1.00
**Asfc**	0.82	0.79	0.37	-0.17	0.92	0.84	-0.49	0.74	-0.56	-0.35	-0.26	0.98
**Das**	0.82	0.79	0.37	-0.17	0.92	0.84	-0.49	0.74	-0.56	-0.35	-0.26	0.98
**Smfc**	0.57	0.56	0.41	-0.19	0.48	0.53	-0.47	0.31	-0.51	-0.36	-0.24	0.49
**HAsfc**	0.49	0.45	0.34	-0.19	0.42	0.53	-0.47	0.33	-0.50	-0.33	-0.29	0.47
**MedianAsfc**	0.75	0.75	0.35	-0.14	0.85	0.74	-0.43	0.69	-0.51	-0.30	-0.20	0.89
**epLsar**	-0.01	-0.01	-0.10	-0.05	0.05	0.01	0.04	-0.02	0.03	0.03	0.01	0.02
**NewEplsar**	-0.09	-0.10	-0.14	0.13	-0.01	-0.05	0.16	-0.06	0.17	0.10	0.07	-0.04
**Isotropy**	-0.25	-0.28	-0.13	1.00	-0.21	-0.21	0.28	-0.16	0.31	0.18	0.10	-0.20
**Maximum depth of furrows**	0.78	0.74	0.42	-0.18	0.88	0.85	-0.59	0.65	-0.62	-0.38	-0.26	0.89
**Mean depth of furrows**	0.88	0.87	0.54	-0.24	0.95	0.88	-0.64	0.74	-0.74	-0.35	-0.31	0.96
**Mean density of furrows**	-0.63	-0.62	-0.54	0.22	-0.61	-0.64	0.71	-0.28	0.78	0.22	0.28	-0.59

	Asfc	Das	Smfc	HAsfc	MedianAsfc	epLsar	NewEplsar	Isotropy	Maximum depth of furrows	Mean depth of furrows	Mean density of furrows
**Sq**	0.82	0.82	0.57	0.49	0.75	-0.01	-0.09	-0.25	0.78	0.88	-0.63
**Smc**	0.79	0.79	0.56	0.45	0.75	-0.01	-0.10	-0.28	0.74	0.87	-0.62
**Sal**	0.37	0.37	0.41	0.34	0.35	-0.10	-0.14	-0.13	0.42	0.54	-0.54
**Str**	-0.17	-0.17	-0.19	-0.19	-0.14	-0.05	0.13	1.00	-0.18	-0.24	0.22
**Sdr**	0.92	0.92	0.48	0.42	0.85	0.05	-0.01	-0.21	0.88	0.95	-0.61
**Vvv**	0.84	0.84	0.53	0.53	0.74	0.01	-0.05	-0.21	0.85	0.88	-0.64
**Spd**	-0.49	-0.49	-0.47	-0.47	-0.43	0.04	0.16	0.28	-0.59	-0.64	0.71
**Spc**	0.74	0.74	0.31	0.33	0.69	-0.02	-0.06	-0.16	0.65	0.74	-0.28
**Svd**	-0.56	-0.56	-0.51	-0.50	-0.51	0.03	0.17	0.31	-0.62	-0.74	0.78
**Shrnq**	-0.35	-0.35	-0.36	-0.33	-0.30	0.03	0.10	0.18	-0.38	-0.35	0.22
**Sdrnq**	-0.26	-0.26	-0.24	-0.29	-0.20	0.01	0.07	0.10	-0.26	-0.31	0.28
**Y max**	0.98	0.98	0.49	0.47	0.89	0.02	-0.04	-0.20	0.89	0.96	-0.59
**Asfc**	1.00	1.00	0.46	0.46	0.90	0.05	0.00	-0.17	0.88	0.93	-0.56
**Das**	1.00	1.00	0.46	0.46	0.90	0.05	0.00	-0.17	0.88	0.93	-0.56
**Smfc**	0.46	0.46	1.00	0.48	0.47	0.04	-0.08	-0.19	0.45	0.50	-0.45
**HAsfc**	0.46	0.46	0.48	1.00	0.21	0.03	-0.04	-0.19	0.51	0.45	-0.38
**MedianAsfc**	0.90	0.90	0.47	0.21	1.00	0.05	0.00	-0.14	0.76	0.86	-0.52
**epLsar**	0.05	0.05	0.04	0.03	0.05	1.00	0.87	-0.05	0.03	0.00	-0.07
**NewEplsar**	0.00	0.00	-0.08	-0.04	0.00	0.87	1.00	0.13	-0.03	-0.08	0.03
**Isotropy**	-0.17	-0.17	-0.19	-0.19	-0.14	-0.05	0.13	1.00	-0.18	-0.24	0.22
**Maximum depth of furrows**	0.88	0.88	0.45	0.51	0.76	0.03	-0.03	-0.18	1.00	0.86	-0.56
**Mean depth of furrows**	0.93	0.93	0.50	0.45	0.86	0.00	-0.08	-0.24	0.86	1.00	-0.71
**Mean density of furrows**	-0.56	-0.56	-0.45	-0.38	-0.52	-0.07	0.03	0.22	-0.56	-0.71	1.00

S4 Table. Descriptive statistics for linear roughness variables used in the technique analysis.

Measurement units of the variables: Sal (mm), Str (unitless), Spc (1/mm), Shrnq (unitless), *Sdrnq (unitless), Smfc (µm²), HAsfc (unitless), MedianAsfc (unitless), NewEplsar (unitless), Mean density of furrows (cm/cm²).*

Techniques:
CS: Control Surface;
PIPB: Pecking in Indirect Percussion with a Blade;
PIPP: Pecking in Indirect Percussion with a Pick;
PDPP: Pecking in Direct Percussion with a Pick;
PDPC: Pecking in Direct Percussion with a Cobble;
PSc: Pecking in direct percussion with a broken blade and Scraping with an endScraper;
ScS: Scraping with an endScraper;
ScB: Scraping with a Blade;
PScPo: Pecking in direct percussion with a broken blade, Scraping with an endscraper and Polishing with Skin;
PoHS: Polishing with Humid Sand;
PoS: Polishing with Skin;
PoC: Polishing with a Cobble.

Statistical descriptor: *Min = Minimum, CI = Confidence Interval (calculated using Quantiles), Max = Maximum, Q = Quartile, SD = Standard Deviation, NMAD = Normalized Median Absolute Deviation, sqrt BWMV = Square root of the Biweight Midvariance. CIs were calculated using 95% confidence intervals.*

Variable	Technique	Min	CI lower	Q1	Median	Mean	SD	NMAD	sqrt BWMV	Q3	CI upper	Max
Sal	CS	0.51	0.52	0.59	0.60	0.61	0.05	0.05	0.05	0.64	0.67	0.68
	PIPB	0.74	0.75	0.94	1.03	1.02	0.14	0.11	0.14	1.10	1.35	1.37
	PIPP	0.70	0.89	1.03	1.08	1.09	0.13	0.09	0.11	1.15	1.40	1.48
	PDPP	0.72	0.86	0.96	1.07	1.07	0.14	0.14	0.14	1.14	1.40	1.41
	PDPC	0.81	0.88	0.98	1.09	1.08	0.13	0.13	0.13	1.17	1.30	1.34
	PSc	0.56	0.58	0.76	0.93	0.93	0.23	0.26	0.23	1.05	1.45	1.51
	ScS	0.60	0.66	0.77	0.91	0.93	0.19	0.23	0.19	1.08	1.31	1.35
	ScB	0.52	0.56	0.74	0.89	0.88	0.18	0.22	0.18	1.01	1.14	1.28
	PScPo	0.55	0.57	0.70	0.80	0.84	0.18	0.17	0.18	0.97	1.22	1.38
	PoHS	0.50	0.54	0.69	0.76	0.77	0.14	0.11	0.14	0.84	1.06	1.16
	PoS	0.51	0.59	0.68	0.72	0.74	0.12	0.09	0.11	0.80	1.03	1.11
	PoC	0.50	0.55	0.62	0.70	0.71	0.12	0.13	0.12	0.79	1.03	1.06
Str	CS	-0.90	-0.87	-0.77	-0.03	-0.15	0.61	0.84	0.61	0.37	0.64	0.70
	PIPB	-0.68	-0.65	0.07	0.26	0.21	0.34	0.25	0.32	0.42	0.73	0.82
	PIPP	-0.68	-0.56	0.05	0.29	0.21	0.35	0.23	0.33	0.43	0.76	0.94
	PDPP	-0.55	-0.44	-0.05	0.16	0.19	0.33	0.35	0.34	0.44	0.83	0.94
	PDPC	-0.35	-0.18	0.16	0.36	0.33	0.31	0.28	0.31	0.50	0.91	1.03
	PSc	-0.55	-0.35	0.20	0.38	0.38	0.36	0.30	0.36	0.60	1.06	1.12
	ScS	-0.57	-0.54	-0.08	0.13	0.12	0.36	0.37	0.37	0.45	0.60	0.96
	ScB	-0.50	-0.40	0.20	0.31	0.35	0.35	0.28	0.35	0.56	0.95	1.06
	PScPo	-0.35	-0.12	0.21	0.46	0.46	0.33	0.34	0.33	0.66	1.22	1.29
	PoHS	-0.62	-0.19	0.20	0.47	0.47	0.38	0.41	0.37	0.75	1.07	1.20
	PoS	-0.21	-0.12	0.29	0.47	0.46	0.29	0.30	0.29	0.67	0.98	1.06
	PoC	-0.21	0.25	0.44	0.55	0.55	0.24	0.23	0.23	0.70	0.96	1.06

Variable	Technique	Min	CI lower	Q1	Median	Mean	SD	NMAD	sqrt BWMV	Q3	CI upper	Max
Spc	CS	2.07	2.09	2.29	2.38	2.44	0.25	0.33	0.25	2.66	2.77	2.78
	PIPB	3.16	3.25	4.34	5.14	5.29	1.29	1.35	1.28	6.14	8.36	8.64
	PIPP	2.87	3.37	4.30	5.22	5.69	1.91	1.66	1.78	6.87	10.54	10.88
	PDPP	3.13	3.63	4.41	4.96	5.22	1.15	1.01	1.13	5.88	7.15	8.74
	PDPC	2.46	2.76	3.71	4.28	4.19	0.75	0.73	0.74	4.68	5.21	6.24
	PSc	1.99	2.22	2.71	3.07	3.08	0.55	0.56	0.56	3.45	4.03	4.20
	ScS	1.93	2.28	3.23	3.99	3.89	0.96	0.85	1.00	4.42	5.72	5.97
	ScB	2.40	2.55	3.06	3.53	3.75	0.85	0.90	0.90	4.42	5.30	5.31
	PScPo	2.03	2.62	3.37	3.68	3.67	0.57	0.48	0.57	4.03	4.62	4.99
	PoHS	2.07	2.12	2.72	3.13	3.16	0.70	0.57	0.60	3.39	4.72	5.77
	PoS	2.47	2.61	2.96	3.41	3.41	0.52	0.65	0.53	3.81	4.33	4.51
	PoC	1.99	2.04	2.55	2.90	2.95	0.59	0.54	0.58	3.27	4.28	4.56
Shrnq	CS	0.09	0.09	0.11	0.12	0.11	0.01	0.01	0.01	0.12	0.13	0.13
	PIPB	0.00	0.00	0.05	0.09	0.08	0.04	0.04	0.04	0.11	0.15	0.15
	PIPP	0.00	0.00	0.03	0.07	0.07	0.05	0.07	0.05	0.12	0.14	0.14
	PDPP	0.00	0.00	0.06	0.09	0.08	0.05	0.04	0.05	0.11	0.15	0.17
	PDPC	0.03	0.04	0.08	0.11	0.11	0.03	0.03	0.03	0.13	0.16	0.17
	PSc	0.01	0.02	0.09	0.11	0.10	0.03	0.02	0.02	0.12	0.14	0.15
	ScS	0.03	0.05	0.10	0.12	0.11	0.03	0.02	0.02	0.12	0.15	0.17
	ScB	0.00	0.02	0.09	0.10	0.10	0.03	0.03	0.03	0.12	0.15	0.16
	PScPo	0.01	0.05	0.11	0.12	0.11	0.02	0.01	0.01	0.12	0.14	0.18
	PoHS	0.08	0.08	0.10	0.11	0.11	0.02	0.01	0.02	0.12	0.14	0.14
	PoS	0.00	0.09	0.10	0.11	0.11	0.02	0.01	0.01	0.12	0.14	0.14
	PoC	0.08	0.08	0.10	0.11	0.11	0.02	0.02	0.02	0.12	0.14	0.15

Variable	Technique	Min	CI lower	Q1	Median	Mean	SD	NMAD	sqrt BWMV	Q3	CI upper	Max
Sdrnq	CS	0.10	0.10	0.10	0.11	0.11	0.01	0.02	0.01	0.12	0.13	0.13
	PIPB	0.00	0.00	0.06	0.09	0.08	0.05	0.03	0.05	0.11	0.16	0.19
	PIPP	0.00	0.00	0.05	0.08	0.08	0.05	0.05	0.05	0.11	0.17	0.19
	PDPP	0.00	0.00	0.05	0.11	0.09	0.05	0.04	0.06	0.12	0.18	0.18
	PDPC	0.00	0.00	0.06	0.09	0.08	0.04	0.04	0.04	0.11	0.15	0.18
	PSc	0.04	0.06	0.10	0.12	0.11	0.02	0.02	0.02	0.13	0.15	0.15
	ScS	0.00	0.00	0.09	0.11	0.10	0.03	0.02	0.02	0.12	0.15	0.19
	ScB	0.00	0.03	0.09	0.11	0.10	0.03	0.02	0.02	0.12	0.15	0.16
	PScPo	0.04	0.08	0.10	0.11	0.11	0.01	0.01	0.01	0.12	0.13	0.15
	PoHS	0.07	0.08	0.10	0.11	0.11	0.02	0.02	0.02	0.12	0.14	0.14
	PoS	0.07	0.09	0.10	0.11	0.11	0.01	0.01	0.01	0.12	0.13	0.14
	PoC	0.08	0.08	0.10	0.11	0.11	0.02	0.02	0.02	0.12	0.14	0.14
Smfc	CS	96190	100818	119331	119331	135195	38230	0	NA	148039	211876	227835
	PIPB	132263	134623	239402	459755	560875	446978	376755	326508	760654	1798582	2286651
	PIPP	130388	150923	360810	487665	722461	686221	288102	267802	757029	2795564	3425720
	PDPP	109507	139655	211601	380200	518107	555742	186733	220343	506150	1480973	3623009
	PDPC	89037	171349	262077	378934	455991	350600	175862	162381	505317	1488473	1878589
	PSc	107075	109701	155090	208586	237937	172583	79313	77860	237837	509581	1195944
	ScS	130847	135498	206187	265283	442550	410227	157780	188857	501798	1472798	2324431
	ScB	105970	106062	133148	198389	225101	138739	91059	92049	256468	587307	768758
	PScPo	67004	83636	104273	120009	147227	76121	32048	38904	169397	316040	630386
	PoHS	54359	69409	111225	138326	147155	60016	49970	54737	174381	269968	335587
	PoS	70748	72666	109546	133867	132354	38332	35221	34689	140106	209896	261245
	PoC	68951	106979	133254	143000	157197	39371	41343	43028	177641	220018	220673

Variable	Technique	Min	CI lower	Q1	Median	Mean	SD	NMAD	sqrt BWMV	Q3	CI upper	Max
HAsfc	CS	0.18	0.19	0.22	0.23	0.24	0.04	0.02	0.03	0.26	0.30	0.31
	PIPB	0.10	0.19	0.29	0.50	0.51	0.24	0.27	0.25	0.67	0.98	1.10
	PIPP	0.17	0.19	0.38	0.54	0.69	0.60	0.28	0.28	0.78	2.78	3.36
	PDPP	0.18	0.21	0.31	0.45	0.49	0.24	0.22	0.22	0.63	1.06	1.18
	PDPC	0.13	0.17	0.27	0.32	0.36	0.16	0.10	0.11	0.41	0.81	0.84
	PSc	0.14	0.16	0.26	0.32	0.34	0.14	0.14	0.13	0.42	0.71	0.74
	ScS	0.13	0.19	0.26	0.35	0.44	0.33	0.15	0.14	0.47	1.35	1.90
	ScB	0.11	0.13	0.25	0.32	0.37	0.18	0.14	0.17	0.43	0.74	0.81
	PScPo	0.10	0.11	0.17	0.21	0.23	0.08	0.08	0.08	0.27	0.42	0.49
	PoHS	0.10	0.14	0.21	0.25	0.29	0.13	0.08	0.09	0.34	0.62	0.84
	PoS	0.09	0.12	0.17	0.21	0.22	0.08	0.07	0.07	0.26	0.41	0.44
	PoC	0.17	0.17	0.24	0.27	0.32	0.15	0.10	0.11	0.38	0.54	1.02
MedianAsfc	CS	7.04	7.09	7.48	7.84	8.15	1.00	0.82	0.96	8.92	9.90	10.11
	PIPB	13.23	13.42	27.38	36.50	38.62	18.41	13.52	16.99	45.32	85.20	92.88
	PIPP	5.31	15.12	29.45	34.36	38.50	20.33	9.93	13.09	41.86	95.52	121.92
	PDPP	19.90	22.67	29.25	36.18	41.17	18.74	12.59	12.62	49.08	93.58	116.04
	PDPC	12.96	17.08	21.86	26.90	29.36	9.84	8.08	9.45	33.04	49.37	57.31
	PSc	7.57	9.06	11.21	13.80	14.92	4.43	4.74	4.58	18.75	24.04	24.23
	ScS	3.06	4.40	17.80	23.22	22.36	10.02	8.03	10.24	28.12	42.43	44.52
	ScB	8.34	8.89	18.19	22.47	22.49	7.57	6.68	7.38	26.97	34.20	45.40
	PScPo	7.22	8.94	12.33	14.98	16.41	5.64	5.25	5.54	19.41	28.54	35.87
	PoHS	7.21	8.34	11.47	14.63	15.29	4.61	4.67	4.67	17.75	24.58	25.35
	PoS	8.22	9.03	12.50	15.50	16.09	5.52	5.17	5.12	19.09	29.10	32.04
	PoC	3.81	7.13	11.42	13.02	15.04	5.59	4.40	5.15	17.88	25.92	33.63

Variable	Technique	Min	CI lower	Q1	Median	Mean	SD	NMAD	sqrt BWMV	Q3	CI upper	Max
NewEplsar	CS	0.0227	0.0227	0.0228	0.0228	0.0229	0.0002	0.0001	0.0002	0.0230	0.0231	0.0231
	PIPB	0.0161	0.0162	0.0173	0.0178	0.0179	0.0009	0.0010	0.0009	0.0186	0.0198	0.0204
	PIPP	0.0165	0.0165	0.0173	0.0176	0.0177	0.0008	0.0007	0.0007	0.0183	0.0197	0.0202
	PDPP	0.0158	0.0162	0.0173	0.0178	0.0178	0.0008	0.0010	0.0008	0.0184	0.0190	0.0196
	PDPC	0.0167	0.0169	0.0174	0.0176	0.0177	0.0004	0.0004	0.0005	0.0180	0.0183	0.0188
	PSc	0.0167	0.0168	0.0174	0.0177	0.0191	0.0032	0.0008	0.0005	0.0183	0.0254	0.0254
	ScS	0.0154	0.0159	0.0168	0.0172	0.0171	0.0006	0.0006	0.0006	0.0176	0.0181	0.0182
	ScB	0.0167	0.0168	0.0171	0.0174	0.0175	0.0005	0.0004	0.0005	0.0178	0.0183	0.0187
	PScPo	0.0170	0.0172	0.0177	0.0180	0.0189	0.0020	0.0007	0.0007	0.0189	0.0235	0.0236
	PoHS	0.0169	0.0173	0.0175	0.0179	0.0179	0.0005	0.0006	0.0005	0.0182	0.0188	0.0190
	PoS	0.0172	0.0175	0.0179	0.0180	0.0181	0.0004	0.0003	0.0004	0.0184	0.0187	0.0191
	PoC	0.0172	0.0173	0.0176	0.0182	0.0182	0.0007	0.0007	0.0007	0.0185	0.0198	0.0199
Mean density of furrows	CS	16.81	16.87	17.17	17.30	17.44	0.49	0.31	0.45	17.51	18.28	18.32
	PIPB	13.90	14.17	15.23	15.85	15.74	0.73	0.72	0.73	16.17	16.99	17.04
	PIPP	14.02	14.13	15.22	15.63	15.70	0.81	0.80	0.84	16.43	16.99	17.29
	PDPP	14.04	14.33	15.36	15.85	15.87	0.77	0.81	0.76	16.42	17.46	17.55
	PDPC	14.18	14.20	14.89	15.32	15.40	0.80	0.70	0.74	15.81	17.26	17.86
	PSc	15.02	15.09	16.48	17.01	16.97	0.84	1.03	0.84	17.71	18.22	18.33
	ScS	13.40	14.28	15.56	16.06	15.96	0.81	0.73	0.78	16.54	17.16	17.33
	ScB	15.12	15.33	16.07	16.62	16.72	0.85	0.99	0.88	17.45	18.30	18.33
	PScPo	15.70	15.92	16.95	17.65	17.60	0.90	0.96	0.92	18.28	19.20	19.92
	PoHS	14.45	15.60	16.70	17.19	17.31	1.13	1.11	1.11	18.16	19.77	20.01
	PoS	16.27	16.39	17.07	17.45	17.56	0.68	0.69	0.71	18.11	18.66	19.01
	PoC	16.28	16.38	16.96	17.44	17.58	0.83	0.82	0.82	18.12	19.56	19.78

S5 Table. Summary of LDA statistics by class for the technique analysis: overall data and by skill level. *Pos Pred Value = Positive Predictive Value*, *Neg Pred Value = Negative Predictive Value.*

	**Technique**	**Sensitivity**	**Specificity**	**Pos Pred Value**	**Neg Pred Value**	**Precision**	**Recall**	**F1**	**Prevalence**	**Detection Rate**	**Detection Prevalence**	**Balanced Accuracy**
Overall data	PDPC	0.34	0.95	0.47	0.92	0.47	0.34	0.40	0.11	0.04	0.08	0.65
	PDPP	0.14	0.92	0.13	0.93	0.13	0.14	0.14	0.08	0.01	0.08	0.53
	PIPB	0.24	0.93	0.16	0.96	0.16	0.24	0.19	0.05	0.01	0.08	0.58
	PIPP	0.33	0.94	0.30	0.95	0.30	0.33	0.31	0.07	0.02	0.08	0.64
	PoC	0.33	0.95	0.42	0.92	0.42	0.33	0.37	0.11	0.04	0.08	0.64
	PoHS	0.23	0.93	0.16	0.95	0.16	0.23	0.18	0.06	0.01	0.08	0.58
	PoS	0.24	0.92	0.09	0.97	0.09	0.24	0.13	0.03	0.01	0.08	0.58
	PSc	0.30	0.93	0.20	0.96	0.20	0.30	0.24	0.06	0.02	0.08	0.61
	PScPo	0.44	0.92	0.66	0.84	0.66	0.44	0.53	0.25	0.11	0.17	0.68
	ScB	0.27	0.93	0.24	0.94	0.24	0.27	0.26	0.08	0.02	0.08	0.60
	ScS	0.36	0.95	0.47	0.93	0.47	0.36	0.41	0.11	0.04	0.08	0.66
Novice	PDPC	0.51	0.97	0.70	0.93	0.70	0.51	0.59	0.13	0.07	0.10	0.74
	PDPP	0.15	0.94	0.11	0.96	0.11	0.15	0.13	0.05	0.01	0.06	0.55
	PIPB	0.33	0.95	0.22	0.97	0.22	0.33	0.27	0.04	0.01	0.06	0.64
	PIPP	0.33	0.95	0.28	0.96	0.28	0.33	0.30	0.05	0.02	0.06	0.64
	PoC	0.48	0.93	0.37	0.96	0.37	0.48	0.42	0.08	0.04	0.10	0.71
	PoHS	0.38	0.93	0.37	0.94	0.37	0.38	0.38	0.09	0.04	0.10	0.66
	PoS	0.19	0.91	0.19	0.92	0.19	0.19	0.19	0.09	0.02	0.10	0.55
	PSc	0.56	0.93	0.37	0.97	0.37	0.56	0.44	0.06	0.04	0.10	0.75
	PScPo	0.53	0.91	0.67	0.86	0.67	0.53	0.59	0.24	0.13	0.19	0.72
	ScB	0.33	0.95	0.33	0.95	0.33	0.33	0.33	0.06	0.02	0.06	0.64
	ScS	0.24	0.95	0.33	0.93	0.33	0.24	0.28	0.09	0.02	0.06	0.60

	Technique	Sensitivity	Specificity	Pos Pred Value	Neg Pred Value	Precision	Recall	F1	Prevalence	Detection Rate	Detection Prevalence	Balanced Accuracy
Intermediate	PDPC	0.31	0.96	0.44	0.94	0.44	0.31	0.36	0.09	0.03	0.06	0.64
	PDPP	0.32	0.91	0.33	0.90	0.33	0.32	0.32	0.13	0.04	0.12	0.61
	PIPB	0.22	0.89	0.11	0.95	0.11	0.22	0.15	0.06	0.01	0.12	0.56
	PIPP	0.50	0.92	0.35	0.96	0.35	0.50	0.41	0.08	0.04	0.11	0.71
	PoC	0.56	0.97	0.56	0.97	0.56	0.56	0.56	0.06	0.03	0.06	0.76
	PoHS	0.63	0.97	0.56	0.98	0.56	0.63	0.59	0.05	0.03	0.06	0.80
	PoS	0.50	0.97	0.56	0.97	0.56	0.50	0.53	0.07	0.03	0.06	0.74
	PSc	0.50	0.95	0.22	0.99	0.22	0.50	0.31	0.03	0.01	0.06	0.73
	PScPo	0.68	0.98	0.83	0.95	0.83	0.68	0.75	0.14	0.10	0.12	0.83
	ScB	0.19	0.90	0.28	0.84	0.28	0.19	0.23	0.17	0.03	0.12	0.54
	ScS	0.10	0.88	0.11	0.87	0.11	0.10	0.11	0.13	0.01	0.12	0.49
Expert	PDPC	0.67	0.97	0.67	0.97	0.67	0.67	0.67	0.08	0.06	0.08	0.82
	PDPP	0.67	0.95	0.44	0.98	0.44	0.67	0.53	0.06	0.04	0.08	0.81
	PIPB	0.57	0.95	0.44	0.97	0.44	0.57	0.50	0.06	0.04	0.08	0.76
	PIPP	0.50	0.96	0.56	0.95	0.56	0.50	0.53	0.09	0.05	0.08	0.73
	PoC	0.45	0.96	0.56	0.94	0.56	0.45	0.50	0.10	0.05	0.08	0.71
	PoHS	0.50	0.97	0.67	0.94	0.67	0.50	0.57	0.11	0.06	0.08	0.73
	PoS	0.44	0.95	0.44	0.95	0.44	0.44	0.44	0.08	0.04	0.08	0.70
	PSc	0.33	0.93	0.22	0.96	0.22	0.33	0.27	0.06	0.02	0.08	0.63
	PScPo	0.68	0.94	0.72	0.93	0.72	0.68	0.70	0.18	0.12	0.17	0.81
	ScB	0.22	0.93	0.22	0.93	0.22	0.22	0.22	0.08	0.02	0.08	0.58
	ScS	0.90	1.00	1.00	0.99	1.00	0.90	0.95	0.09	0.08	0.08	0.95

S6 Table. LDA Confusion matrices for the technique analysis: overall data and by skill level.

		PDPC	PDPP	PIPB	PIPP	PoC	PoHS	PoS	PSc	PScPo	ScB	ScS
**Overall data**	**PDPC**	21	6	6	1	0	0	0	0	2	1	8
	**PDPP**	12	6	8	11	0	0	0	0	0	2	6
	**PIPB**	6	12	7	11	0	0	0	1	0	4	4
	**PIPP**	7	14	5	13	0	0	0	2	0	1	2
	**PoC**	0	0	0	0	19	10	0	1	15	0	0
	**PoHS**	0	0	0	0	14	7	1	2	13	3	5
	**PoS**	0	0	0	0	14	5	4	1	18	2	1
	**PSc**	2	0	0	1	5	5	2	9	18	2	1
	**PScPo**	3	0	0	0	2	2	8	12	59	3	1
	**ScB**	3	1	3	0	4	2	2	2	8	11	9
	**ScS**	7	3	0	2	0	0	0	0	0	12	21
**Novice**	**PDPC**	19	2	1	2	0	0	0	0	0	0	3
	**PDPP**	6	2	2	2	0	0	0	0	0	1	5
	**PIPB**	1	6	4	6	0	0	0	0	0	1	0
	**PIPP**	5	2	5	5	0	0	0	0	0	0	1
	**PoC**	0	0	0	0	10	7	2	0	8	0	0
	**PoHS**	0	0	0	0	4	10	5	1	3	0	4
	**PoS**	0	0	0	0	5	3	5	0	11	2	1
	**PSc**	1	0	0	0	1	2	3	10	7	2	1
	**PScPo**	0	0	0	0	1	2	6	6	36	2	1
	**ScB**	1	1	0	0	0	2	2	0	3	6	3
	**ScS**	4	0	0	0	0	0	3	1	0	4	6
**Intermediate**	**PDPC**	4	0	1	0	0	0	0	1	0	2	1
	**PDPP**	1	6	3	3	0	0	0	0	1	2	2
	**PIPB**	1	6	2	0	0	0	0	0	0	5	4
	**PIPP**	2	5	0	6	0	0	0	0	1	1	2
	**PoC**	0	0	0	0	5	3	1	0	0	0	0
	**PoHS**	0	0	0	0	2	5	2	0	0	0	0
	**PoS**	0	0	0	0	2	0	5	1	1	0	0
	**PSc**	1	0	0	0	0	0	1	2	3	2	0
	**PScPo**	2	0	0	0	0	0	1	0	15	0	0
	**ScB**	1	0	2	0	0	0	0	0	1	5	9
	**ScS**	1	2	1	3	0	0	0	0	0	9	2
**Expert**	**PDPC**	6	0	1	0	0	0	2	0	0	0	0
	**PDPP**	1	4	1	1	0	0	0	0	1	0	1
	**PIPB**	1	2	4	2	0	0	0	0	0	0	0
	**PIPP**	1	0	1	5	0	1	0	1	0	0	0
	**PoC**	0	0	0	0	5	1	0	1	0	2	0
	**PoHS**	0	0	0	0	0	6	0	1	0	2	0
	**PoS**	0	0	0	0	0	0	4	0	3	2	0
	**PSc**	0	0	0	2	1	2	0	2	1	1	0
	**PScPo**	0	0	0	0	0	1	3	1	13	0	0
	**ScB**	0	0	0	0	5	1	0	0	1	2	0
	**ScS**	0	0	0	0	0	0	0	0	0	0	9

S7 Table. Normality and group comparison tests for the analysis of engravings.

Type of engravings	Parameter	Test	Shapiro *p*	Shapiro *W*	FRP (%)	Df	Test statistic	*p*-value
Superficial	Depth	kruskal	9.6E-09	0.78	4.83E-05	6	30.46	3.21E-05
	WIS	kruskal	8.6E-04	0.93	1.63	6	29.37	5.17E-05
	A	kruskal	1.3E-05	0.88	0.04	6	30.36	3.36E-05
Deep	Depth	ANOVA	0.43	0.95	/	1	0.26	0.62
	WIS	ANOVA	0.17	0.93	/	1	1.01	0.33
	A	ANOVA	0.86	0.98	/	1	0.50	0.49

S8 Table. Summary of linear statistical parameters (Depth, WIS, A) in the engraving analysis.

WIS: Width in surface; A: Asymmetry

All dimensions are in µm.

Types of engravings:

Set A: Engravings produced with a burin using multiple strokes;
Set B: Engravings produced with a flake using multiple strokes;
Set C: Engravings produced with a blade using multiple strokes;
Set D: Pecking + Scraping + Polishing + Engravings produced with a blade using single stroke;
Set E: Pecking + Scraping + Polishing + Engravings produced with a blade using multiple strokes;
Set F: Pecking + Scraping + Engravings produced with a blade using single stroke + Polishing;
Set G: Pecking + Scraping + Engravings produced with a blade using multiple stroke + Polishing.

Statistical descriptor: *Min = Minimum, CI = Confidence Interval (calculated using Quantiles), Max = Maximum, Q = Quartile, SD = Standard Deviation, NMAD = Normalized Median Absolute Deviation, sqrt BWMV = Square root of the Biweight Midvariance. CIs were calculated using 95% confidence intervals.*

Type of engravings	Variable	Set	Shapiro *W*	Shapiro *p*	Min	CI lower	Q1	Median	Mean	SD	Q3	CI upper	Max
Superficial	Depth	Set A	0.97	0.90	568.82	815.52	767.19	966.07	978.72	298.84	1147.02	1146.57	1562.09
		Set B	0.79	0.01	575.50	658.47	639.07	688.85	797.64	261.08	874.19	968.82	1408.82
		Set C	0.89	0.22	382.95	506.39	515.86	584.91	635.48	225.85	698.52	790.36	1113.73
		Set D	0.90	0.24	178.91	285.26	249.74	336.53	373.27	155.25	508.66	469.60	602.50
		Set E	0.80	0.01	270.63	456.21	360.52	568.76	825.34	680.12	1032.09	1251.51	2272.19
		Set F	0.84	0.05	73.67	185.63	169.10	200.84	300.24	205.02	451.18	422.28	643.65
		Set G	0.76	0.01	276.06	565.69	489.27	751.61	1070.47	987.19	1080.59	1723.26	3500.31
	WIS	Set A	0.93	0.43	3526.78	4178.26	4078.18	4523.05	4604.93	732.84	5211.05	5005.68	5530.87
		Set B	0.94	0.53	2383.37	2697.36	2632.01	2889.90	2953.58	415.94	3263.03	3209.79	3572.72
		Set C	0.97	0.88	1988.92	2283.72	2319.71	2438.32	2505.03	356.24	2705.11	2734.87	3162.22
		Set D	0.89	0.16	1537.60	2340.73	2496.17	2868.92	2909.04	993.86	2963.38	3510.03	4607.10
		Set E	0.88	0.12	1875.89	2931.07	2727.81	3235.58	3985.78	1916.07	4917.51	5107.79	8142.29
		Set F	0.85	0.06	1172.79	1638.25	1395.93	1995.35	2213.85	1059.68	2397.63	2890.60	4238.45
		Set G	0.93	0.44	2177.73	3410.08	3255.24	4478.79	4316.31	1617.54	4575.93	5252.48	7425.15
	A	Set A	0.99	1.00	1.23	1.78	1.75	2.13	2.14	0.56	2.43	2.45	3.10
		Set B	0.92	0.32	1.03	1.46	1.38	1.85	1.75	0.45	2.04	2.00	2.31
		Set C	0.86	0.11	1.27	1.47	1.40	1.56	1.81	0.58	2.09	2.22	2.94
		Set D	0.91	0.27	2.51	3.04	2.73	3.44	3.60	0.98	4.30	4.20	4.20
		Set E	0.83	0.03	1.13	1.96	1.95	2.30	2.65	1.28	2.69	3.45	5.32
		Set F	0.90	0.21	2.05	2.95	2.63	3.28	3.91	1.74	4.77	4.95	7.49
		Set G	0.97	0.87	0.73	1.60	1.66	2.16	2.12	0.90	2.67	2.66	3.61
Deep	Depth	Set H	0.94	0.54	8692.97	10720.98	10576.56	12080.6	12004.39	2176.39	13419.90	13287.51	15024.11
		Set I	0.97	0.86	6463.91	9904.04	10429.75	11268.44	11462.00	2586.25	13370.09	12946.31	15133.71
	WIS	Set H	0.94	0.55	17018.70	21937.72	20380.70	27033.5	26025.89	6682.19	29422.54	29874.19	38104.89
		Set I	0.88	0.13	16539.11	23876.76	19507.69	33779.76	29967.00	10433.84	37906.64	35762.56	44777.94
	A	Set H	0.93	0.44	0.42	0.67	0.71	0.8181026	0.77	0.16	0.86	0.86	1.00
		Set I	0.95	0.65	0.52	0.71	0.72	0.8701153	0.83	0.20	0.95	0.94	1.13

S9 Table. Summary of circular statistics for the variable OA (opening angle) for the engraved sets.

**Type of engravings**	**Set**	**Rayleight *Z***	**Rayleigh *p***	**Skewness**	**Kurtosis**	**Circular Variance**	**Circular Dispersion**	**Central Orientation (rad)**	**Central Orientation (°)**	**Circular Median**
Superficial	All sets	0.962	<2.2E-16	1.548	0.387	0.038	0.077	2.378	136.255	136.424
	Set A	0.989	<2.2E-16	-0.429	-0.776	0.011	0.023	2.322	133.026	131.848
	Set B	0.977	<2.2E-16	3.167	0.668	0.023	0.046	2.151	123.245	128.151
	Set C	0.979	<2.2E-16	-1.305	-1.953	0.021	0.043	2.188	125.351	121.330
	Set D	0.994	<2.2E-16	0.579	-2.149	0.006	0.012	2.620	150.143	150.278
	Set E	0.969	<2.2E-16	1.983	1.434	0.031	0.062	2.406	137.856	136.976
	Set F	0.984	<2.2E-16	1.214	-0.673	0.016	0.033	2.619	150.068	149.075
	Set G	0.949	<2.2E-16	2.652	-0.032	0.051	0.105	2.297	131.619	136.348
Deep	All sets	0.985	<2.2E-16	0.628	-1.285	0.015	0.031	1.641	93.997	95.687
	Set H	0.994	<2.2E-16	1.509	-2.385	0.007	0.013	1.580	90.527	92.806
	Set I	0.980	<2.2E-16	2.244	-0.788	0.020	0.041	1.702	97.515	99.613

S10 Table. MANOVA based on PC1 and PC2 from the metric PCA to assess intergroup variation among superficial engravings.

	Set A	Set B	Set C	Set D	Set E	Set F
**Set B**	0.003	-	-	-	-	-
**Set C**	0.003	0.113	-	-	-	-
**Set D**	0.003	0.003	0.005	-	-	-
**Set E**	0.709	0.048	0.028	0.168	-	-
**Set F**	0.003	0.003	0.013	0.340	0.042	-
**Set G**	0.375	0.007	0.003	0.105	0.823	0.011

S11 Table. MANOVA based on PC1 and PC2 from the EFA PCA to assess intergroup variation among superficial engravings.

	Set A	Set B	Set C	Set D	Set E	Set F
**Set B**	0.541	-	-	-	-	-
**Set C**	0.102	0.976	-	-	-	-
**Set D**	0.006	0.004	0.004	-	-	-
**Set E**	0.913	0.432	0.014	0.388	-	-
**Set F**	0.008	0.004	0.004	0.976	0.188	-
**Set G**	0.040	0.006	0.004	0.188	0.092	0.432

S12 Table. Normality and group comparison tests for the analysis of expertise

**Variable**	**Test**	**Shapiro *p***	**Shapiro *W***	**Df**	**Test statistic**	***p*-value**
Sq	kruskal	1.5E-21	8.6E-01	2	40.74	1.42E-09
Smc	kruskal	5.0E-23	8.4E-01	2	32.50	8.78E-08
Sal	kruskal	1.8E-06	9.8E-01	2	31.26	1.63E-07
Strn norm	kruskal	9.9E-04	9.9E-01	2	14.37	0.0008
Sdr norm	kruskal	2.0E-05	9.8E-01	2	32.45	8.99E-08
Vvv	kruskal	1.6E-16	9.2E-01	2	48.93	2.37E-11
Spd	kruskal	1.9E-14	9.4E-01	2	42.19	6.90E-10
Spc	kruskal	9.8E-19	9.0E-01	2	6.26	0.0436
Svd	kruskal	5.1E-13	9.5E-01	2	24.86	4.00E-06
Shrn	kruskal	3.2E-10	9.6E-01	2	4.81	0.0902
Shrnq	kruskal	4.8E-20	8.8E-01	2	4.65	0.0978
Sdrn	kruskal	8.4E-15	9.3E-01	2	5.28	0.0713
Sdrnq	kruskal	3.2E-20	8.8E-01	2	2.12	0.3462
Y max	kruskal	7.0E-24	8.2E-01	2	32.99	6.84E-08
Asfc	kruskal	8.4E-24	8.3E-01	2	36.75	1.05E-08
Das	kruskal	8.4E-24	8.3E-01	2	36.75	1.05E-08
Smfc	kruskal	9.5E-35	5.6E-01	2	15.64	0.0004
HAsfc	kruskal	9.4E-32	6.5E-01	2	19.77	5.08E-05
MedianAsfc	kruskal	1.0E-24	8.1E-01	2	39.23	3.03E-09
epLsar	kruskal	2.9E-35	5.4E-01	2	6.95	0.0309
NewEplsar	kruskal	3.3E-31	6.7E-01	2	49.19	2.09E-11
Isotropy norm	kruskal	9.9E-04	9.9E-01	2	14.37	0.0008
Maximum depth of furrows	kruskal	6.7E-18	9.0E-01	2	49.07	2.21E-11
Mean depth of furrows	kruskal	3.4E-18	9.0E-01	2	24.90	3.92E-06
Mean density of furrows	ANOVA	2.0E-01	1.0E+00	2	9.52	8.69E-05

S13 Table. Statistical results of angular data for the analysis of expertise. *Wg and Pg are the test statistics for the corresponding p-values (p).*

**Variable**	**Test**	**Df**	***Wg***	***p*-value**	**Test 2**	***Pg***	***p*-value**
Std	Mardia-Watson-Wheeler	2	7.00	0.03	Fisher's Non-Parametric	0.18	0.67
First Direction	Mardia-Watson-Wheeler	2	16.45	0.00	Fisher's Non-Parametric	3.17	0.07
Second Direction	Mardia-Watson-Wheeler	2	1.64	0.44	Fisher's Non-Parametric	0.00	0.97
Third Direction	Mardia-Watson-Wheeler	2	4.02	0.13	Fisher's Non-Parametric	2.28	0.13

S14 Table. Correlation matrix values for expertise analysis.

	Sq	Smc	Sal	Str	Sdr	Vvv	Spd	Svd	Y max	Asfc	Das
**Sq**	1.00	0.86	0.63	-0.25	0.87	0.87	-0.69	-0.76	0.87	0.82	0.82
**Smc**	0.86	1.00	0.63	-0.28	0.84	0.83	-0.64	-0.74	0.84	0.79	0.79
**Sal**	0.63	0.63	1.00	-0.13	0.51	0.64	-0.77	-0.79	0.45	0.37	0.37
**Str**	-0.25	-0.28	-0.13	1.00	-0.21	-0.21	0.28	0.31	-0.20	-0.17	-0.17
**Sdr**	0.87	0.84	0.51	-0.21	1.00	0.89	-0.61	-0.68	0.94	0.92	0.92
**Vvv**	0.87	0.83	0.64	-0.21	0.89	1.00	-0.72	-0.77	0.88	0.84	0.84
**Spd**	-0.69	-0.64	-0.77	0.28	-0.61	-0.72	1.00	0.91	-0.56	-0.49	-0.49
**Svd**	-0.76	-0.74	-0.79	0.31	-0.68	-0.77	0.91	1.00	-0.64	-0.56	-0.56
**Y max**	0.87	0.84	0.45	-0.20	0.94	0.88	-0.56	-0.64	1.00	0.98	0.98
**Asfc**	0.82	0.79	0.37	-0.17	0.92	0.84	-0.49	-0.56	0.98	1.00	1.00
**Das**	0.82	0.79	0.37	-0.17	0.92	0.84	-0.49	-0.56	0.98	1.00	1.00
**Smfc**	0.57	0.56	0.41	-0.19	0.48	0.53	-0.47	-0.51	0.49	0.46	0.46
**HAsfc**	0.49	0.45	0.34	-0.19	0.42	0.53	-0.47	-0.50	0.47	0.46	0.46
**MedianAsfc**	0.75	0.75	0.35	-0.14	0.85	0.74	-0.43	-0.51	0.89	0.90	0.90
**NewEplsar**	-0.09	-0.10	-0.14	0.13	-0.01	-0.05	0.16	0.17	-0.04	0.00	0.00
**Isotropy**	-0.25	-0.28	-0.13	1.00	-0.21	-0.21	0.28	0.31	-0.20	-0.17	-0.17
**Maximum depth of furrows**	0.78	0.74	0.42	-0.18	0.88	0.85	-0.59	-0.62	0.89	0.88	0.88
**Mean depth of furrows**	0.88	0.87	0.54	-0.24	0.95	0.88	-0.64	-0.74	0.96	0.93	0.93
**Mean density of furrows**	-0.63	-0.62	-0.54	0.22	-0.61	-0.64	0.71	0.78	-0.59	-0.56	-0.56
**First Direction**	0.01	0.01	0.00	0.00	0.02	0.01	0.01	0.01	0.01	0.01	0.01

	Smfc	HAsfc	MedianAsfc	NewEplsar	Isotropy	Maximum depth of furrows	Mean depth of furrows	Mean density of furrows	First Direction
**Sq**	0.57	0.49	0.75	-0.09	-0.25	0.78	0.88	-0.63	0.01
**Smc**	0.56	0.45	0.75	-0.10	-0.28	0.74	0.87	-0.62	0.01
**Sal**	0.41	0.34	0.35	-0.14	-0.13	0.42	0.54	-0.54	0.00
**Str**	-0.19	-0.19	-0.14	0.13	1.00	-0.18	-0.24	0.22	0.00
**Sdr**	0.48	0.42	0.85	-0.01	-0.21	0.88	0.95	-0.61	0.02
**Vvv**	0.53	0.53	0.74	-0.05	-0.21	0.85	0.88	-0.64	0.01
**Spd**	-0.47	-0.47	-0.43	0.16	0.28	-0.59	-0.64	0.71	0.01
**Svd**	-0.51	-0.50	-0.51	0.17	0.31	-0.62	-0.74	0.78	0.01
**Y max**	0.49	0.47	0.89	-0.04	-0.20	0.89	0.96	-0.59	0.01
**Asfc**	0.46	0.46	0.90	0.00	-0.17	0.88	0.93	-0.56	0.01
**Das**	0.46	0.46	0.90	0.00	-0.17	0.88	0.93	-0.56	0.01
**Smfc**	1.00	0.48	0.47	-0.08	-0.19	0.45	0.50	-0.45	0.01
**HAsfc**	0.48	1.00	0.21	-0.04	-0.19	0.51	0.45	-0.38	0.00
**MedianAsfc**	0.47	0.21	1.00	0.00	-0.14	0.76	0.86	-0.52	0.01
**NewEplsar**	-0.08	-0.04	0.00	1.00	0.13	-0.03	-0.08	0.03	0.03
**Isotropy**	-0.19	-0.19	-0.14	0.13	1.00	-0.18	-0.24	0.22	0.00
**Maximum depth of furrows**	0.45	0.51	0.76	-0.03	-0.18	1.00	0.86	-0.56	0.02
**Mean depth of furrows**	0.50	0.45	0.86	-0.08	-0.24	0.86	1.00	-0.71	0.02
**Mean density of furrows**	-0.45	-0.38	-0.52	0.03	0.22	-0.56	-0.71	1.00	0.01
**First Direction**	0.01	0.00	0.01	0.03	0.00	0.02	0.02	0.01	1.00

S15 Table. Descriptive statistics for linear roughness variables used in the expertise analysis.

Measurement units of the variables: Sal (mm), *Smfc (µm²), HAsfc (unitless), MedianAsfc (unitless), NewEplsar (unitless), Isotropy (unitless), Mean density of furrows (cm/cm²).*

Statistical descriptor: *Min = Minimum, CI = Confidence Interval (calculated using Quantiles), Max = Maximum, Q = Quartile, SD = Standard Deviation, NMAD = Normalized Median Absolute Deviation, sqrt BWMV = Square root of the Biweight Midvariance. CIs were calculated using 95% confidence intervals.*

Variable	Technique	Expertise	Min	CI lower	Q1	Median	Mean	SD	NMAD	sqrtBWMV	Q3	CI upper	Max
Sal	PIPB	Novice	0.74	0.77	0.95	1.06	1.02	0.14	0.08	0.13	1.09	1.28	1.36
		Intermediate	0.75	0.79	0.92	0.97	0.99	0.12	0.11	0.12	1.06	1.22	1.26
		Expert	0.92	0.92	1.02	1.04	1.10	0.15	0.16	0.16	1.20	1.35	1.37
	PIPP	Novice	0.89	0.93	1.04	1.09	1.10	0.12	0.08	0.09	1.15	1.36	1.48
		Intermediate	0.70	0.80	0.96	1.04	1.05	0.16	0.08	0.12	1.09	1.36	1.41
		Expert	1.01	1.02	1.12	1.13	1.13	0.07	0.04	0.06	1.16	1.24	1.26
	PDPP	Novice	0.72	0.79	0.95	1.05	1.04	0.16	0.14	0.16	1.10	1.37	1.41
		Intermediate	0.86	0.88	0.96	1.05	1.06	0.13	0.13	0.12	1.12	1.33	1.41
		Expert	0.95	0.97	1.08	1.11	1.15	0.12	0.14	0.12	1.25	1.30	1.31
	PDPC	Novice	0.89	0.91	0.95	1.06	1.05	0.11	0.13	0.11	1.10	1.25	1.30
		Intermediate	1.03	1.03	1.10	1.13	1.13	0.08	0.06	0.08	1.17	1.28	1.30
		Expert	0.81	0.83	1.01	1.12	1.11	0.19	0.23	0.19	1.27	1.33	1.34
	PSc	Novice	0.61	0.61	0.74	0.93	0.90	0.19	0.20	0.20	1.01	1.25	1.30
		Intermediate	0.81	0.84	1.02	1.07	1.10	0.20	0.17	0.18	1.18	1.45	1.51
		Expert	0.56	0.56	0.73	0.76	0.86	0.31	0.14	0.20	0.86	1.44	1.47
	ScS	Novice	0.60	0.63	0.75	0.92	0.92	0.21	0.23	0.21	1.04	1.34	1.35
		Intermediate	0.71	0.72	0.83	0.97	0.96	0.17	0.22	0.17	1.12	1.16	1.18
		Expert	0.65	0.67	0.78	0.86	0.92	0.19	0.26	0.19	1.08	1.17	1.19
	ScB	Novice	0.58	0.62	0.75	0.92	0.88	0.16	0.19	0.16	0.96	1.13	1.14
		Intermediate	0.67	0.71	0.87	0.98	0.97	0.16	0.16	0.16	1.09	1.22	1.28
		Expert	0.52	0.53	0.66	0.68	0.70	0.12	0.08	0.12	0.74	0.89	0.89
	PScPo	Novice	0.57	0.59	0.72	0.79	0.80	0.13	0.13	0.13	0.88	1.07	1.13
		Intermediate	0.85	0.90	1.00	1.08	1.10	0.13	0.14	0.13	1.20	1.34	1.38
		Expert	0.55	0.56	0.64	0.69	0.71	0.12	0.10	0.10	0.77	0.97	1.05
	PoHS	Novice	0.54	0.54	0.70	0.75	0.76	0.13	0.12	0.14	0.84	1.00	1.01
		Intermediate	0.50	0.53	0.68	0.72	0.70	0.09	0.09	0.08	0.78	0.79	0.79
		Expert	0.66	0.66	0.76	0.81	0.86	0.18	0.20	0.18	1.01	1.14	1.16
	PoS	Novice	0.51	0.56	0.65	0.69	0.70	0.09	0.10	0.09	0.77	0.85	0.87
		Intermediate	0.70	0.70	0.72	0.78	0.83	0.15	0.08	0.12	0.87	1.09	1.11
		Expert	0.63	0.64	0.73	0.82	0.79	0.12	0.12	0.12	0.83	0.97	0.99
	PoC	Novice	0.50	0.54	0.61	0.70	0.70	0.12	0.13	0.12	0.78	0.94	1.04
		Intermediate	0.56	0.56	0.66	0.68	0.71	0.10	0.14	0.10	0.79	0.86	0.87
		Expert	0.54	0.55	0.66	0.70	0.75	0.16	0.19	0.16	0.84	1.02	1.06

Variable	Technique	Expertise	Min	CI lower	Q1	Median	Mean	SD	NMAD	sqrtBWMV	Q3	CI upper	Max
Smfc	PIPB	Novice	153393	169644	239402	478267	705661	616708	418667	497129	760654	2094630	2286651
		Intermediate	132263	146074	330811	367752	413427	180509	161969	179871	494243	766964	766964
		Expert	132538	139048	205637	616604	566202	353963	224528	389883	768046	1106931	1191652
	PIPP	Novice	150063	185994	298185	486209	731427	892865	217615	181990	573428	3177447	3425720
		Intermediate	161527	216721	376844	486551	765595	597767	277305	295752	756278	2050845	2228411
		Expert	130388	136813	488779	759284	623056	327280	222517	346730	759284	1095451	1179493
	PDPP	Novice	195446	195446	227069	385087	446295	243551	211241	236813	486196	934150	942195
		Intermediate	170063	170063	224522	331550	354769	173955	112764	123293	407608	761796	899408
		Expert	109507	114861	506150	506150	988406	1089889	548376	547938	1213909	3200537	3623009
	PDPC	Novice	209129	209129	267453	403347	463230	356278	193507	172203	503695	1445741	1878589
		Intermediate	243140	255219	303536	378934	443115	272636	111785	74410	378934	1013835	1149029
		Expert	89037	105499	171349	329758	447148	434448	267517	232991	510196	1317334	1519118
	PSc	Novice	134587	134587	167550	208586	225338	89047	60841	69185	237837	441309	505945
		Intermediate	137569	137569	171143	212911	249039	116512	77039	90277	264874	473892	509986
		Expert	107075	107075	133340	166049	264634	350708	48494	40144	166049	998111	1195944
	ScS	Novice	132902	147762	206187	256819	439394	502028	115608	161021	472982	1667026	2324431
		Intermediate	130847	142753	247202	280795	467560	409050	167388	186607	487501	1472798	1472798
		Expert	171413	190187	265283	330021	398840	175688	235153	184394	510749	635390	635390
	ScB	Novice	105970	105970	133264	167453	173857	48999	54539	49417	210246	263774	263774
		Intermediate	132733	146583	205912	256468	325981	168362	110620	144073	394667	696692	768758
		Expert	106895	106895	106895	106895	125830	25255	0	NA	133148	165848	165848
	PScPo	Novice	87379	87379	108913	137672	148496	57322	42870	49235	170793	280134	326464
		Intermediate	109654	109654	110811	142002	187656	126543	47959	50130	176444	506330	630386
		Expert	67004	74031	104149	104273	102992	16455	183	95	104273	129847	129847
	PoHS	Novice	90464	90678	112859	140287	168694	63877	50548	63344	216135	292934	335587
		Intermediate	54359	57035	84417	84417	99079	38964	24724	34646	105199	163369	163369
		Expert	89433	93792	111225	138326	130616	23612	0	NA	138326	165289	172030
	PoS	Novice	70748	81984	109546	136312	137294	42317	39684	38455	140106	227868	261245
		Intermediate	86341	90575	107509	133867	134563	35621	39078	33339	133867	199380	207554
		Expert	71146	74619	110111	110111	115325	24054	39842	23833	136984	136984	136984
	PoC	Novice	111280	111280	136913	143000	159370	35907	41343	39449	174897	220673	220673
		Intermediate	68951	76556	106979	133254	135710	40319	38955	38947	165983	198596	206749
		Expert	110322	110322	137266	170790	172166	43844	61841	42930	212501	212501	212501

Variable	Technique	Expertise	Min	CI lower	Q1	Median	Mean	SD	NMAD	sqrtBWMV	Q3	CI upper	Max
HAsfc	PIPB	Novice	0.10	0.15	0.26	0.50	0.53	0.30	0.36	0.30	0.75	1.01	1.10
		Intermediate	0.18	0.19	0.29	0.42	0.45	0.20	0.21	0.20	0.59	0.80	0.81
		Expert	0.33	0.34	0.50	0.62	0.59	0.19	0.13	0.19	0.67	0.92	0.98
	PIPP	Novice	0.17	0.21	0.35	0.39	0.49	0.27	0.11	0.16	0.55	1.13	1.31
		Intermediate	0.19	0.21	0.41	0.66	0.78	0.71	0.36	0.27	0.82	2.43	3.36
		Expert	0.37	0.38	0.54	0.68	0.95	0.78	0.38	0.30	0.93	2.57	2.90
	PDPP	Novice	0.18	0.19	0.30	0.38	0.44	0.21	0.17	0.17	0.52	0.89	1.07
		Intermediate	0.26	0.26	0.31	0.46	0.47	0.17	0.24	0.17	0.63	0.75	0.77
		Expert	0.24	0.25	0.34	0.46	0.64	0.36	0.33	0.40	0.94	1.13	1.18
	PDPC	Novice	0.17	0.17	0.28	0.32	0.34	0.13	0.09	0.08	0.40	0.60	0.84
		Intermediate	0.27	0.27	0.32	0.35	0.44	0.18	0.09	0.15	0.55	0.77	0.82
		Expert	0.13	0.14	0.19	0.27	0.35	0.22	0.13	0.19	0.39	0.71	0.71
	PSc	Novice	0.14	0.15	0.23	0.32	0.33	0.12	0.15	0.12	0.42	0.55	0.58
		Intermediate	0.18	0.19	0.28	0.32	0.32	0.09	0.05	0.09	0.33	0.45	0.45
		Expert	0.22	0.23	0.32	0.33	0.43	0.19	0.07	0.12	0.53	0.74	0.74
	ScS	Novice	0.13	0.16	0.23	0.29	0.43	0.41	0.13	0.16	0.49	1.41	1.90
		Intermediate	0.31	0.31	0.33	0.39	0.45	0.23	0.10	0.09	0.46	1.00	1.31
		Expert	0.19	0.19	0.21	0.26	0.42	0.37	0.11	0.14	0.43	1.19	1.36
	ScB	Novice	0.12	0.16	0.27	0.32	0.39	0.19	0.13	0.18	0.53	0.76	0.81
		Intermediate	0.16	0.18	0.32	0.37	0.42	0.17	0.10	0.16	0.47	0.73	0.75
		Expert	0.11	0.12	0.17	0.23	0.22	0.07	0.09	0.07	0.26	0.31	0.32
	PScPo	Novice	0.10	0.11	0.17	0.19	0.22	0.08	0.06	0.08	0.26	0.40	0.49
		Intermediate	0.14	0.15	0.22	0.29	0.29	0.09	0.09	0.09	0.33	0.45	0.48
		Expert	0.12	0.12	0.16	0.20	0.20	0.05	0.06	0.05	0.24	0.27	0.28
	PoHS	Novice	0.14	0.16	0.21	0.25	0.30	0.15	0.08	0.08	0.32	0.70	0.84
		Intermediate	0.10	0.11	0.16	0.25	0.24	0.09	0.14	0.09	0.34	0.35	0.35
		Expert	0.20	0.20	0.24	0.28	0.32	0.11	0.11	0.11	0.38	0.52	0.55
	PoS	Novice	0.14	0.15	0.17	0.23	0.24	0.08	0.08	0.08	0.29	0.38	0.44
		Intermediate	0.17	0.17	0.20	0.22	0.24	0.07	0.04	0.05	0.24	0.39	0.42
		Expert	0.09	0.10	0.14	0.16	0.17	0.05	0.05	0.05	0.20	0.25	0.26
	PoC	Novice	0.17	0.18	0.23	0.28	0.34	0.17	0.12	0.12	0.43	0.71	1.02
		Intermediate	0.17	0.17	0.24	0.25	0.28	0.10	0.08	0.09	0.32	0.47	0.49
		Expert	0.20	0.21	0.26	0.27	0.30	0.08	0.03	0.06	0.34	0.45	0.46

Variable	Technique	Expertise	Min	CI lower	Q1	Median	Mean	SD	NMAD	sqrtBWMV	Q3	CI upper	Max
MedianAsfc	PIPB	Novice	20.44	21.96	30.53	39.29	42.34	16.74	13.77	14.93	48.65	78.67	86.93
		Intermediate	20.29	20.49	29.25	38.58	42.37	19.36	15.33	17.46	48.81	83.00	92.88
		Expert	13.23	13.26	13.76	18.45	23.66	12.54	7.52	10.11	28.25	47.23	49.80
	PIPP	Novice	28.01	28.78	32.59	39.53	41.20	15.98	9.59	7.73	42.38	81.05	98.14
		Intermediate	21.24	23.52	31.49	35.78	44.97	23.49	11.04	14.16	54.76	98.46	121.92
		Expert	5.31	7.27	15.25	17.20	20.89	10.98	3.77	6.36	22.98	38.76	39.04
	PDPP	Novice	22.64	22.83	28.53	35.95	36.08	9.42	12.39	9.52	43.61	50.09	50.21
		Intermediate	26.22	26.84	31.96	42.63	47.43	22.30	18.22	16.81	55.60	100.29	116.04
		Expert	19.90	20.50	23.18	31.89	38.85	23.08	13.31	12.18	42.87	85.67	95.20
	PDPC	Novice	21.86	22.74	27.24	30.41	34.42	9.24	7.81	9.64	41.19	52.25	57.31
		Intermediate	18.03	18.17	18.92	21.07	22.77	4.20	4.51	4.48	26.07	28.78	29.06
		Expert	12.96	13.77	17.82	21.21	20.75	4.38	5.03	4.27	24.30	26.33	26.54
	PSc	Novice	9.74	9.84	13.55	16.11	16.49	4.35	4.01	4.56	18.97	24.15	24.23
		Intermediate	9.04	9.25	10.61	11.03	12.46	3.61	2.31	2.22	12.82	19.73	21.10
		Expert	7.57	7.89	10.57	12.62	12.69	3.53	3.04	3.43	13.49	18.61	19.15
	ScS	Novice	9.69	12.63	18.77	22.25	22.14	5.60	5.04	5.13	24.50	32.56	35.85
		Intermediate	18.86	19.05	23.60	29.47	29.57	7.67	8.52	7.68	32.00	43.82	44.52
		Expert	3.06	3.32	4.92	6.82	8.39	4.90	3.67	4.17	10.25	17.62	19.08
	ScB	Novice	11.89	13.20	18.30	21.71	21.85	5.22	5.09	5.10	25.01	31.25	33.79
		Intermediate	18.42	19.34	23.09	26.86	27.60	6.37	5.51	5.63	30.22	40.66	45.40
		Expert	8.34	8.43	10.01	11.46	13.54	4.67	4.60	5.00	17.47	20.66	21.09
	PScPo	Novice	9.23	9.62	13.71	16.63	17.82	5.69	5.02	5.51	21.92	29.22	35.87
		Intermediate	7.22	7.38	9.28	10.73	11.15	2.72	2.46	2.35	12.22	16.79	18.95
		Expert	12.15	12.18	13.94	16.02	17.41	4.63	4.65	4.79	20.73	25.40	25.51
	PoHS	Novice	10.22	10.27	11.55	14.11	14.48	3.79	3.69	3.41	16.03	24.54	24.58
		Intermediate	11.43	12.87	18.78	21.73	20.49	3.98	2.28	2.74	22.17	24.93	25.35
		Expert	7.21	7.39	11.05	12.19	12.52	3.59	6.02	3.47	16.36	16.94	17.07
	PoS	Novice	8.22	8.73	12.06	14.96	16.67	6.64	6.24	6.52	20.76	30.16	32.04
		Intermediate	9.27	9.41	11.29	13.78	13.18	2.64	3.47	2.63	14.13	16.93	17.13
		Expert	12.45	13.18	16.68	17.07	17.28	2.21	2.84	2.10	19.09	19.24	19.25
	PoC	Novice	3.81	5.87	11.53	12.76	15.07	6.50	5.04	5.92	18.37	28.67	33.63
		Intermediate	10.62	11.06	15.14	17.71	17.25	4.08	3.82	4.08	19.44	22.74	22.82
		Expert	10.05	10.18	11.08	11.87	12.74	2.54	1.68	2.30	14.21	17.34	17.88

Variable	Technique	Expertise	Min	CI lower	Q1	Median	Mean	SD	NMAD	sqrtBWMV	Q3	CI upper	Max
NewEplsar	PIPB	Novice	0.0161	0.0165	0.0175	0.0184	0.0183	0.0011	0.0012	0.0010	0.0189	0.0201	0.0204
		Intermediate	0.0162	0.0163	0.0170	0.0173	0.0174	0.0006	0.0007	0.0006	0.0178	0.0186	0.0186
		Expert	0.0175	0.0175	0.0178	0.0183	0.0183	0.0006	0.0008	0.0006	0.0188	0.0191	0.0191
	PIPP	Novice	0.0165	0.0168	0.0174	0.0177	0.0178	0.0006	0.0005	0.0006	0.0183	0.0187	0.0187
		Intermediate	0.0165	0.0165	0.0169	0.0173	0.0175	0.0009	0.0006	0.0007	0.0180	0.0195	0.0202
		Expert	0.0174	0.0174	0.0178	0.0179	0.0181	0.0007	0.0003	0.0004	0.0183	0.0195	0.0198
	PDPP	Novice	0.0173	0.0175	0.0180	0.0183	0.0183	0.0005	0.0004	0.0005	0.0185	0.0193	0.0196
		Intermediate	0.0165	0.0166	0.0171	0.0176	0.0176	0.0007	0.0005	0.0007	0.0179	0.0188	0.0189
		Expert	0.0158	0.0159	0.0166	0.0168	0.0170	0.0008	0.0009	0.0008	0.0176	0.0184	0.0186
	PDPC	Novice	0.0167	0.0168	0.0174	0.0176	0.0177	0.0005	0.0004	0.0005	0.0180	0.0185	0.0188
		Intermediate	0.0174	0.0174	0.0176	0.0178	0.0178	0.0003	0.0004	0.0004	0.0182	0.0183	0.0183
		Expert	0.0169	0.0170	0.0171	0.0175	0.0175	0.0004	0.0005	0.0004	0.0178	0.0179	0.0180
	PSc	Novice	0.0167	0.0167	0.0175	0.0181	0.0202	0.0037	0.0011	0.0008	0.0252	0.0254	0.0254
		Intermediate	0.0171	0.0171	0.0174	0.0176	0.0175	0.0003	0.0002	0.0003	0.0177	0.0180	0.0181
		Expert	0.0170	0.0170	0.0171	0.0174	0.0175	0.0005	0.0004	0.0005	0.0178	0.0184	0.0186
	ScS	Novice	0.0161	0.0163	0.0169	0.0174	0.0173	0.0005	0.0006	0.0005	0.0177	0.0179	0.0179
		Intermediate	0.0159	0.0162	0.0169	0.0171	0.0172	0.0006	0.0005	0.0005	0.0176	0.0182	0.0182
		Expert	0.0154	0.0155	0.0164	0.0165	0.0166	0.0006	0.0008	0.0006	0.0171	0.0171	0.0172
	ScB	Novice	0.0168	0.0168	0.0174	0.0177	0.0176	0.0004	0.0004	0.0004	0.0180	0.0181	0.0181
		Intermediate	0.0167	0.0167	0.0170	0.0172	0.0173	0.0005	0.0004	0.0004	0.0173	0.0183	0.0187
		Expert	0.0171	0.0172	0.0173	0.0176	0.0176	0.0004	0.0005	0.0004	0.0179	0.0183	0.0183
	PScPo	Novice	0.0170	0.0171	0.0178	0.0181	0.0196	0.0024	0.0009	0.0010	0.0221	0.0236	0.0236
		Intermediate	0.0173	0.0173	0.0174	0.0177	0.0177	0.0003	0.0003	0.0003	0.0179	0.0180	0.0181
		Expert	0.0171	0.0172	0.0177	0.0184	0.0183	0.0007	0.0008	0.0007	0.0188	0.0192	0.0192
	PoHS	Novice	0.0169	0.0172	0.0175	0.0179	0.0178	0.0004	0.0005	0.0004	0.0180	0.0186	0.0187
		Intermediate	0.0173	0.0173	0.0174	0.0175	0.0177	0.0004	0.0003	0.0005	0.0181	0.0183	0.0183
		Expert	0.0179	0.0179	0.0180	0.0184	0.0184	0.0004	0.0005	0.0004	0.0186	0.0189	0.0190
	PoS	Novice	0.0172	0.0175	0.0179	0.0181	0.0182	0.0004	0.0004	0.0004	0.0185	0.0188	0.0191
		Intermediate	0.0175	0.0175	0.0178	0.0179	0.0180	0.0004	0.0005	0.0004	0.0183	0.0186	0.0186
		Expert	0.0177	0.0178	0.0180	0.0180	0.0181	0.0002	0.0001	0.0001	0.0182	0.0184	0.0185
	PoC	Novice	0.0175	0.0176	0.0178	0.0183	0.0185	0.0008	0.0010	0.0008	0.0192	0.0198	0.0199
		Intermediate	0.0172	0.0173	0.0178	0.0181	0.0180	0.0004	0.0003	0.0004	0.0183	0.0184	0.0185
		Expert	0.0173	0.0173	0.0174	0.0176	0.0178	0.0004	0.0005	0.0004	0.0180	0.0185	0.0185

Variable	Technique	Expertise	Min	CI lower	Q1	Median	Mean	SD	NMAD	sqrtBWMV	Q3	CI upper	Max
Isotropy	PIPB	Novice	-0.47	-0.41	0.03	0.30	0.22	0.32	0.24	0.31	0.42	0.66	0.74
		Intermediate	-0.67	-0.49	0.11	0.28	0.25	0.33	0.29	0.29	0.47	0.63	0.67
		Expert	-0.68	-0.58	-0.09	0.20	0.12	0.42	0.31	0.39	0.25	0.74	0.82
	PIPP	Novice	-0.56	-0.33	0.20	0.35	0.31	0.29	0.19	0.22	0.44	0.76	0.76
		Intermediate	-0.52	-0.46	0.08	0.32	0.24	0.36	0.22	0.32	0.43	0.79	0.94
		Expert	-0.68	-0.60	-0.21	-0.07	-0.05	0.35	0.31	0.35	0.14	0.43	0.45
	PDPP	Novice	-0.55	-0.50	-0.07	0.23	0.17	0.41	0.41	0.42	0.47	0.78	0.85
		Intermediate	-0.21	-0.19	-0.01	0.19	0.23	0.31	0.34	0.31	0.43	0.81	0.94
		Expert	-0.11	-0.10	0.01	0.12	0.13	0.19	0.24	0.18	0.28	0.44	0.47
	PDPC	Novice	-0.35	-0.24	0.12	0.36	0.32	0.32	0.35	0.32	0.52	0.82	0.92
		Intermediate	-0.14	-0.13	0.14	0.37	0.31	0.28	0.25	0.28	0.45	0.69	0.73
		Expert	-0.14	-0.08	0.23	0.33	0.37	0.32	0.25	0.28	0.50	0.93	1.03
	PSc	Novice	-0.37	-0.24	0.19	0.33	0.37	0.35	0.38	0.35	0.59	0.98	1.12
		Intermediate	-0.18	-0.12	0.21	0.50	0.44	0.35	0.40	0.34	0.65	0.92	0.96
		Expert	-0.55	-0.44	0.21	0.40	0.32	0.44	0.29	0.39	0.46	0.98	1.07
	ScS	Novice	-0.57	-0.51	-0.17	0.19	0.14	0.38	0.45	0.39	0.49	0.59	0.60
		Intermediate	-0.45	-0.31	-0.02	0.18	0.22	0.32	0.35	0.31	0.40	0.80	0.96
		Expert	-0.55	-0.53	-0.45	-0.08	-0.11	0.34	0.30	0.33	0.11	0.43	0.50
	ScB	Novice	-0.32	-0.17	0.21	0.44	0.43	0.33	0.35	0.33	0.67	0.91	0.97
		Intermediate	-0.40	-0.40	0.15	0.30	0.25	0.34	0.23	0.33	0.41	0.76	0.85
		Expert	-0.50	-0.37	0.26	0.48	0.36	0.41	0.26	0.33	0.50	0.94	1.06
	PScPo	Novice	-0.11	-0.04	0.29	0.52	0.50	0.31	0.31	0.31	0.70	1.20	1.29
		Intermediate	-0.26	-0.08	0.27	0.45	0.44	0.29	0.28	0.27	0.63	0.89	0.91
		Expert	-0.35	-0.26	0.04	0.36	0.37	0.42	0.46	0.41	0.59	1.17	1.29
	PoHS	Novice	-0.20	-0.10	0.20	0.58	0.52	0.38	0.51	0.39	0.75	1.12	1.20
		Intermediate	0.28	0.30	0.57	0.65	0.63	0.19	0.18	0.19	0.77	0.84	0.84
		Expert	-0.62	-0.50	0.05	0.21	0.17	0.36	0.36	0.32	0.46	0.51	0.52
	PoS	Novice	-0.21	-0.15	0.29	0.53	0.49	0.33	0.30	0.33	0.73	1.01	1.06
		Intermediate	-0.05	-0.02	0.29	0.42	0.38	0.25	0.19	0.26	0.55	0.73	0.76
		Expert	0.16	0.17	0.34	0.47	0.45	0.20	0.21	0.20	0.61	0.74	0.77
	PoC	Novice	-0.21	0.09	0.45	0.55	0.56	0.25	0.18	0.23	0.69	0.93	0.96
		Intermediate	0.26	0.27	0.46	0.48	0.57	0.27	0.28	0.26	0.69	1.03	1.06
		Expert	0.26	0.26	0.33	0.50	0.52	0.20	0.30	0.19	0.70	0.77	0.77

Variable	Technique	Expertise	Min	CI lower	Q1	Median	Mean	SD	NMAD	sqrtBWMV	Q3	CI upper	Max
Mean density of furrows	PIPB	Novice	13.90	14.00	14.93	15.63	15.47	0.80	0.79	0.81	16.05	16.52	16.60
		Intermediate	14.71	14.78	15.26	15.90	15.75	0.58	0.61	0.61	16.15	16.53	16.62
		Expert	15.35	15.43	15.78	16.12	16.24	0.62	0.58	0.64	16.97	17.03	17.04
	PIPP	Novice	14.12	14.23	15.02	15.51	15.57	0.84	0.77	0.86	16.03	16.98	17.00
		Intermediate	14.02	14.15	15.13	15.48	15.59	0.85	0.52	0.87	15.83	17.10	17.29
		Expert	15.29	15.36	15.76	16.10	16.17	0.55	0.52	0.56	16.45	16.92	16.94
	PDPP	Novice	14.04	14.14	15.33	15.78	15.72	0.77	0.76	0.75	16.37	16.77	16.85
		Intermediate	14.73	14.79	15.23	15.64	15.66	0.55	0.62	0.55	16.06	16.56	16.63
		Expert	15.54	15.57	16.01	16.47	16.60	0.78	1.14	0.78	17.25	17.53	17.55
	PDPC	Novice	14.18	14.19	14.62	14.92	15.00	0.52	0.52	0.55	15.33	15.89	16.03
		Intermediate	15.11	15.13	15.49	15.59	15.63	0.39	0.18	0.34	15.71	16.34	16.45
		Expert	14.85	15.04	15.82	16.06	16.35	0.92	0.88	0.94	16.95	17.75	17.86
	PSc	Novice	15.02	15.03	16.18	16.79	16.79	0.85	0.93	0.85	17.34	18.15	18.21
		Intermediate	15.54	15.72	16.48	16.62	16.69	0.59	0.32	0.53	16.93	17.60	17.71
		Expert	17.09	17.13	17.58	17.88	17.81	0.42	0.44	0.42	18.09	18.31	18.33
	ScS	Novice	15.11	15.15	15.68	16.44	16.23	0.67	0.62	0.71	16.64	17.20	17.33
		Intermediate	14.59	14.63	15.72	16.14	16.06	0.70	0.65	0.68	16.58	17.12	17.17
		Expert	13.40	13.57	15.11	15.49	15.23	0.93	0.56	0.83	15.61	16.43	16.55
	ScB	Novice	15.64	15.66	16.23	16.89	16.91	0.84	1.02	0.85	17.60	18.22	18.31
		Intermediate	15.12	15.20	15.88	16.10	16.17	0.59	0.57	0.58	16.57	17.25	17.47
		Expert	16.58	16.60	17.24	17.45	17.47	0.60	0.50	0.61	17.79	18.31	18.33
	PScPo	Novice	15.70	15.94	16.70	17.55	17.38	0.84	1.06	0.88	17.99	18.91	19.06
		Intermediate	15.71	15.95	17.10	17.34	17.26	0.62	0.48	0.57	17.67	18.12	18.16
		Expert	17.45	17.56	18.23	18.55	18.59	0.60	0.57	0.59	18.98	19.63	19.92
	PoHS	Novice	14.45	15.19	16.38	16.97	16.89	0.86	0.81	0.82	17.48	18.26	18.29
		Intermediate	17.38	17.55	18.31	18.68	18.82	0.83	0.65	0.86	19.31	19.97	20.01
		Expert	15.75	15.85	16.70	17.08	17.07	0.83	0.57	0.78	17.43	18.44	18.68
	PoS	Novice	16.27	16.35	16.99	17.23	17.45	0.73	0.88	0.77	18.12	18.60	18.67
		Intermediate	16.57	16.67	17.20	17.55	17.52	0.56	0.52	0.55	17.74	18.39	18.47
		Expert	17.13	17.18	17.55	17.91	17.94	0.56	0.54	0.54	18.16	18.89	19.01
	PoC	Novice	16.28	16.45	16.93	17.12	17.34	0.61	0.77	0.66	17.88	18.36	18.36
		Intermediate	18.10	18.12	18.42	18.52	18.74	0.60	0.46	0.57	18.90	19.75	19.78
		Expert	16.36	16.41	16.89	17.05	17.12	0.50	0.58	0.49	17.44	17.89	17.93

S16 Table. Descriptive statistics for circular roughness variables used in the expertise analysis.

Measurement in degrees.

Statistical descriptor: *Min = Minimum, CI = Confidence Interval (calculated using Quantiles), Max = Maximum, Q = Quartile, v = Circular variance, shat = Circular skewness, khat = Circular kurtosis. CIs were calculated using 95% confidence intervals.*

Variable	Technique	Expertise	Min	CI lower	Q1	Median	Mean	*v*	shat	khat	Q3	CI upper	Max
First Direction	PDPC	Expert	0.0009	0.0009	45.0066	153.5112	131.4152	0.6148	1.1612	-0.5407	179.9963	179.9987	179.9987
		Intermediate	44.9775	44.9775	89.9987	116.4802	110.0466	0.2173	0.1236	-0.9550	135.0299	179.9873	179.9873
		Novice	0.0011	0.0011	44.9879	90.0032	99.4624	0.5496	0.1355	-0.6690	179.9879	179.9975	179.9975
	PDPP	Expert	0.0034	0.0034	90.0033	116.4304	127.5738	0.3913	0.6240	0.0376	179.9824	179.9977	179.9977
		Intermediate	0.0005	0.0005	18.5031	63.5076	67.8743	0.4654	-0.6920	-0.3879	116.5004	179.9983	179.9983
		Novice	0.0017	0.0017	90.0025	135.0033	135.5561	0.4490	0.9917	-0.0428	179.9868	179.9989	179.9989
	PIPB	Expert	0.0119	0.0119	116.5180	135.0172	148.7625	0.3116	1.0749	3.3320	179.9983	179.9995	179.9995
		Intermediate	0.0020	0.0020	45.0010	90.0007	100.0033	0.4428	-0.1125	-0.8546	153.5341	179.9972	179.9972
		Novice	0.0011	0.0011	0.0106	89.9992	87.6719	0.6904	-0.2802	-0.9978	179.9900	179.9992	179.9992
	PIPP	Expert	0.0002	0.0002	0.0082	179.9786	150.0327	0.8007	1.0549	0.5203	179.9919	179.9988	179.9988
		Intermediate	0.0050	0.0050	89.9932	90.0033	108.2586	0.3836	0.1893	-0.1352	134.7506	179.9993	179.9993
		Novice	0.0007	0.0007	0.0021	13.2356	32.2546	0.8029	-1.0599	0.5615	179.9876	179.9960	179.9960
	PSc	Expert	0.0042	0.0042	89.9966	90.0019	96.7159	0.1823	2.3605	3.6828	116.5116	135.0115	135.0115
		Intermediate	0.0035	0.0035	0.0093	26.4815	28.3193	0.3299	-1.3514	3.1174	45.0077	179.9887	179.9887
		Novice	0.0003	0.0003	0.0122	90.0035	98.5994	0.5764	0.4293	-0.7499	157.1344	179.9998	179.9998
	PScPo	Expert	0.0049	0.0049	26.4698	179.9851	139.2518	0.7026	1.2234	-0.1837	179.9951	179.9991	179.9991
		Intermediate	0.0048	0.0048	44.9870	89.9937	85.0667	0.5117	-0.5897	-0.5046	179.9875	179.9957	179.9957
		Novice	0.0001	0.0007	0.0111	76.7507	84.8151	0.7505	-0.4008	-1.0389	179.9931	179.9993	179.9998
	PoC	Expert	0.0006	0.0006	0.0041	0.0103	30.9303	0.5978	-1.4900	0.6764	134.9971	179.9966	179.9966
		Intermediate	0.0022	0.0022	0.0102	44.9948	45.0129	0.7317	-1.0653	0.1974	179.9865	179.9982	179.9982
		Novice	0.0006	0.0006	0.0047	44.9947	53.2791	0.6808	-1.0200	-0.2399	179.9947	179.9983	179.9983
	PoHS	Expert	0.0004	0.0004	0.0090	153.5007	138.2325	0.8510	1.1536	0.1410	179.9953	179.9986	179.9986
		Intermediate	0.0047	0.0047	26.4952	45.0034	49.2274	0.3026	-1.6228	2.3710	89.9955	180.0000	180.0000
		Novice	0.0011	0.0011	26.4796	44.9980	51.0009	0.2320	-1.0477	1.0897	89.9994	179.9909	179.9909
	PoS	Expert	0.0009	0.0009	0.0026	0.0073	22.0011	0.8676	-0.9006	0.8312	179.9932	179.9981	179.9981
		Intermediate	0.0009	0.0009	0.0038	0.0087	18.7565	0.6013	-1.4430	1.4469	134.9920	179.9919	179.9919
		Novice	0.0016	0.0016	0.0051	45.0130	81.9334	0.7538	-0.4540	-1.0635	179.9921	179.9984	179.9984
	ScB	Expert	0.0072	0.0072	89.9942	90.0023	102.9833	0.4124	-0.1492	-0.5251	153.5239	179.9999	179.9999
		Intermediate	0.0073	0.0073	45.0173	89.9999	83.0961	0.2980	-0.0398	0.1191	90.0109	179.9910	179.9910
		Novice	0.0023	0.0023	26.4793	123.8963	122.7806	0.6717	0.9669	-0.4376	179.9908	179.9988	179.9988
	ScS	Expert	0.0020	0.0020	0.0147	45.0026	40.8430	0.5140	-1.2512	1.2333	90.0030	179.9978	179.9978
		Intermediate	0.0013	0.0013	44.9845	112.5013	121.0008	0.5912	0.8691	-0.3935	179.9910	179.9997	179.9997
		Novice	0.0023	0.0023	18.4786	54.2445	52.0058	0.4142	-1.1811	1.0563	90.0001	179.9995	179.9995

S17 Table. Summary of LDA statistics by class within technique for the expertise analysis. *Pos Pred Value = Positive Predictive Value*, *Neg Pred Value = Negative Predictive Value.*

		**Sensitivity**	**Specificity**	**Pos Pred Value**	**Neg Pred Value**	**Precision**	**Recall**	**F1**	**Prevalence**	**Detection Rate**	**Detection Prevalence**	**Balanced Accuracy**
PIPB	Expert	0.64	0.94	0.78	0.89	0.78	0.64	0.70	0.24	0.16	0.20	0.79
	Intermediate	0.52	0.73	0.67	0.59	0.67	0.52	0.59	0.51	0.27	0.40	0.62
	Novice	0.64	0.68	0.39	0.85	0.39	0.64	0.48	0.24	0.16	0.40	0.66
PIPP	Expert	0.14	0.78	0.11	0.83	0.11	0.14	0.13	0.16	0.02	0.20	0.46
	Intermediate	0.42	0.64	0.47	0.59	0.47	0.42	0.44	0.43	0.18	0.39	0.53
	Novice	0.44	0.62	0.44	0.62	0.44	0.44	0.44	0.41	0.18	0.41	0.53
PDPP	Expert	0.86	0.92	0.67	0.97	0.67	0.86	0.75	0.16	0.13	0.20	0.89
	Intermediate	0.62	0.79	0.72	0.70	0.72	0.62	0.67	0.47	0.29	0.40	0.71
	Novice	0.76	0.82	0.72	0.85	0.72	0.76	0.74	0.38	0.29	0.40	0.79
PDPC	Expert	0.63	0.89	0.56	0.92	0.56	0.63	0.59	0.18	0.11	0.20	0.76
	Intermediate	0.27	0.82	0.33	0.78	0.33	0.27	0.30	0.24	0.07	0.20	0.55
	Novice	0.85	0.74	0.81	0.78	0.81	0.85	0.83	0.58	0.49	0.60	0.79
PSc	Expert	0.67	0.92	0.67	0.92	0.67	0.67	0.67	0.20	0.13	0.20	0.79
	Intermediate	0.43	0.84	0.33	0.89	0.33	0.43	0.38	0.16	0.07	0.20	0.64
	Novice	0.69	0.56	0.74	0.50	0.74	0.69	0.71	0.64	0.44	0.60	0.63
ScS	Expert	1.00	0.95	0.78	1.00	0.78	1.00	0.88	0.16	0.16	0.20	0.97
	Intermediate	0.69	0.76	0.61	0.81	0.61	0.69	0.65	0.36	0.24	0.40	0.72
	Novice	0.59	0.78	0.72	0.67	0.72	0.59	0.65	0.49	0.29	0.40	0.69
ScB	Expert	0.70	0.94	0.78	0.92	0.78	0.70	0.74	0.22	0.16	0.20	0.82
	Intermediate	0.75	0.79	0.67	0.85	0.67	0.75	0.71	0.36	0.27	0.40	0.77
	Novice	0.58	0.73	0.61	0.70	0.61	0.58	0.59	0.42	0.24	0.40	0.65

		Sensitivity	Specificity	Pos Pred Value	Neg Pred Value	Precision	Recall	F1	Prevalence	Detection Rate	Detection Prevalence	Balanced Accuracy
PScPo	Expert	0.67	0.92	0.67	0.92	0.67	0.67	0.67	0.20	0.13	0.20	0.79
	Intermediate	0.80	0.97	0.89	0.94	0.89	0.80	0.84	0.22	0.18	0.20	0.89
	Novice	0.85	0.74	0.81	0.78	0.81	0.85	0.83	0.58	0.49	0.60	0.79
PoHS	Expert	0.50	0.86	0.44	0.89	0.44	0.50	0.47	0.18	0.09	0.20	0.68
	Intermediate	0.78	0.94	0.78	0.94	0.78	0.78	0.78	0.20	0.16	0.20	0.86
	Novice	0.79	0.71	0.81	0.67	0.81	0.79	0.80	0.62	0.49	0.60	0.75
PoS	Expert	0.43	0.84	0.33	0.89	0.33	0.43	0.38	0.16	0.07	0.20	0.64
	Intermediate	0.25	0.81	0.22	0.83	0.22	0.25	0.24	0.18	0.04	0.20	0.53
	Novice	0.63	0.47	0.70	0.39	0.70	0.63	0.67	0.67	0.42	0.60	0.55
PoC	Expert	0.38	0.84	0.33	0.86	0.33	0.38	0.35	0.18	0.07	0.20	0.61
	Intermediate	0.70	0.94	0.78	0.92	0.78	0.70	0.74	0.22	0.16	0.20	0.82
	Novice	0.74	0.61	0.74	0.61	0.74	0.74	0.74	0.60	0.44	0.60	0.68

S18 Table. LDA confusion matrices by technique for the expertise analysis.

		Expert	Intermediate	Novice
**PIPB**	**Expert**	7	2	0
	**Intermediate**	2	12	4
	**Novice**	2	9	7
**PIPP**	**Expert**	1	4	4
	**Intermediate**	3	8	6
	**Novice**	3	7	8
**PDPP**	**Expert**	6	3	0
	**Intermediate**	1	13	4
	**Novice**	0	5	13
**PDPC**	**Expert**	5	4	0
	**Intermediate**	2	3	4
	**Novice**	1	4	22
**PSc**	**Expert**	6	0	3
	**Intermediate**	0	3	6
	**Novice**	3	4	20
**ScS**	**Expert**	7	0	2
	**Intermediate**	0	11	7
	**Novice**	0	5	13
**ScB**	**Expert**	7	0	2
	**Intermediate**	0	12	6
	**Novice**	3	4	11
**PScPo**	**Expert**	12	0	6
	**Intermediate**	0	16	2
	**Novice**	6	4	44
**PoHS**	**Expert**	4	0	5
	**Intermediate**	1	7	1
	**Novice**	3	2	22
**PoHS**	**Expert**	4	0	5
	**Intermediate**	1	7	1
	**Novice**	3	2	22
**PoS**	**Expert**	3	1	5
	**Intermediate**	1	2	6
	**Novice**	3	5	19
**PoC**	**Expert**	3	1	5
	**Intermediate**	0	7	2
	**Novice**	5	2	20
