# Supplementary material for: Carved in stone: Experimental criteria for identifying Paleolithic bas-relief production techniques and sculptors’ expertise
Source: PLoS One. 2026 Apr 1;21(4):e0346099. doi: 10.1371/journal.pone.0346099 (PMC13042861; doi:10.1371/journal.pone.0346099)
Supplement: S3 File — R scripts used for statistical analyses and figure generation are available online at the GitHub repository: https://github.com/EmBrochard/Sculpture-techniques-expertise_Statistics (PDF) [file pone.0346099.s003.pdf]

Supplementary File 3: R scripts

R scripts used for statistical analyses and figure generation are available online at the GitHub repository:

https://github.com/EmBrochard/Sculpture-techniques-expertise_Statistics

Repository: Sculpture-techniques-expertise_Statistics

Author Details

Authors listed in alphabetical order

Author: Émilie Brochard

Email: emilie.brochard@u-bordeaux.fr

ORCID: https://orcid.org/0009-0003-5864-0844

Current Afiliation: University of Bordeaux [CNRS, PACEA UMR5199]

Contributions: Wrote code for "Roughness".

Author: Lloyd A. Courtenay

Email: ladc1995@gmail.com

ORCID: https://orcid.org/0000-0002-4810-2001

Current Afiliation: University of Bordeaux [CNRS, PACEA UMR5199]

Contributions: Wrote code for "Roughness", "Engraving_Analysis", "Engraving_Extract_Data", "EFA Functions", "Profile_Code".

Author: Luc Doyon

Email: luc.doyon@u-bordeaux.fr

ORCID: https://orcid.org/0000-0001-7163-6186

Current Afiliation: University of Bordeaux [CNRS, PACEA UMR5199]

Contributions: Wrote code for "Roughness"

This code has been designed for the open-source free R programming languages.

Repository Details

The present repository contains:

- **Code**
  - **Source**
    - Code in this source folder is for functions that can be used for the analysis of engraving profiles.
    - EFA Functions.R
      - This is source code that provides functions for Elliptic Fourier Analysis (EFA) for outline-based shape analysis, including normalization, harmonic selection, PCA-based visualization of shape variation, and statistical diagnostics.
    - Profile_Code.R
      - This is source code that provides functions for the extraction, normalization, visualization, and quantitative analysis of 2D profile data, including geometric measurements, asymmetry indices, landmark-based shape characterization, and circular–linear statistical analyses.
  - **Engraving_Extract_Data.R**
    - This R script provides functions for generating profile images, extracting geometric measurements, computing landmarks, and exporting data for morphometric analyses, including writing Morphologika-compatible files and CSV tables.
  - **Engraving_Analysis.R**
    - This main R script implements a complete analytical pipeline for 2D profile data, combining geometric measurements, circular and linear statistics, multivariate analyses, and elliptic Fourier–based shape and form analyses to investigate morphological variability and group differences.
  - **Roughness.R**
    - This code provides a complete workflow for analyzing surface parameters: Step 1. Import data; Step 2. Initial statistics (Kruskal-Wallis, circular tests); Step 3. Filter significant and uncorrelated variables; Step 4. PCA; Step 5. LDA /CVA.

Instructions for Engraving Profile Analysis

The engraving study must follow the following procedure:

- First, create a main folder with a name of your choice, and inside it, insert the folders Dataset and Source:

```
├── Folder name
│   ├── Dataset    (name that can be modified)
│   ├── Source     (EFA Functions.R and  Profile_Code.R – DO NOT MODIFY)
```

Dataset will be where all of the profiles will be stored, and the Source folder is for external functions that the code requires in order to work.

- Second, for the code to work properly, the folder structure must be respected and organized as follows:

```
├── Dataset         (name that can be modified)
│   ├── Images       (name not to modify and keep uppercase + leave empty)
│   ├── Profiles     (name not to modify and keep uppercase + leave empty)
│   │   ├── Group A   (name that can be modified)
│   │   ├── Group B   (name that can be modified)
│   │   ├── Group C   (name that can be modified)
│   │   ├── ...
```

Inside the profiles folder create a series of subfolders that contain your sample labels, e.g. degree of expertise, technique for engravings, etc. This will then be used by the code to sort all of the information and organise the dataset with factorial labels that help us identify where the code comes from

- Turn this into an R project: In R → File → New Project → Existing Directory → paste the path to the folder → OK

- Run the “Engraving_Extract_Data” code. Plotted images of the incision profiles are now available in the “Images” folder within each group.

- Run the “Engraving_Analysis” code to continue the study, including statistics.

System Requirements for Deep Learning

Note that here we specify the versions of the libraries that we used for the present study. We are unaware if earlier or later versions of the same libraries will work or present problems, because we have not tested this, the objective here is simply to state how we ran each of the codes presented.

- R - *v.4.4.1*
- The following R libraries
  - GraphGMM - *v.1.0.0*
  - pValueRobust - *v.0.1.0*

- geomorph - *v.4.0.9*
- shapes - *v.1.2.7*
- ggplot2 - *v.3.5.1*
- circular - *v.0.5.1*
- RVAideMemoire - *v.0.9-83-7*
- dplyr - *v.1.1.4*
- ggpubr - *v.0.6.0*
- writexl - *v.1.5.1*
- corrplot - *v.0.95*
- FactoMineR - *v.2.11*
- factoextra - *v.1.0.7*
- caret - *v.7.0-1*
- MASS - *v.7.3-60.2*
- Morpho - *v.2.12*

Repository Citation

Please cite the code of Roughness analysis as:

Brochard É., Courtenay L.A, Doyon L.(2025) Code for quantitative analysis of surface roughness. https://github.com/EmBrochard/Sculpture-techniques-expertise_Statistics

Please cite the code of Engraving analysis as:

Courtenay L.A (2025) Code for engraving analysis using morphometric measurements and EFA. https://github.com/EmBrochard/Sculpture-techniques-expertise_Statistics

---

Comments, questions, doubts, suggestions and corrections can all be directed to É. Brochard at the email provided above.

License

This project is licensed under the GNU Affero General Public License v3.0. See the LICENSE file for details.

Roughness code: Copyright (C) 2025 Emilie Brochard, Lloyd Courtenay, Luc Doyon

Engraving codes: Copyright (C) 2025 Lloyd Courtenay

This program is free software: you can redistribute it and/or modify it under the terms of the GNU Affero General Public License as published by the Free Software Foundation, version 3.
